# Semaphorin 3A—Glycosaminoglycans Interaction as Therapeutic Target for Axonal Regeneration

**DOI:** 10.3390/ph14090906

**Published:** 2021-09-07

**Authors:** Yolanda Pérez, Roman Bonet, Miriam Corredor, Cecilia Domingo, Alejandra Moure, Àngel Messeguer, Jordi Bujons, Ignacio Alfonso

**Affiliations:** 1 NMR Facility, Institute for Advanced Chemistry of Catalonia (IQAC-CSIC), Jordi Girona 18-26, 08034 Barcelona, Spain; 2 Department of Biological Chemistry, Institute for Advanced Chemistry of Catalonia (IQAC-CSIC), Jordi Girona 18-26, 08034 Barcelona, Spain; rbonet79@gmail.com (R.B.); miriamcorredor5@gmail.com (M.C.); ceci.dbravo@gmail.com (C.D.); sandra_mf_79@hotmail.com (A.M.); amesseguer@gmail.com (À.M.); jordi.bujons@iqac.csic.es (J.B.)

**Keywords:** semaphorin 3A, NMR, glycosaminoglycan–protein interaction, peptoids

## Abstract

Semaphorin 3A (Sema3A) is a cell-secreted protein that participates in the axonal guidance pathways. Sema3A acts as a canonical repulsive axon guidance molecule, inhibiting CNS regenerative axonal growth and propagation. Therefore, interfering with Sema3A signaling is proposed as a therapeutic target for achieving functional recovery after CNS injuries. It has been shown that Sema3A adheres to the proteoglycan component of the extracellular matrix (ECM) and selectively binds to heparin and chondroitin sulfate-E (CS-E) glycosaminoglycans (GAGs). We hypothesize that the biologically relevant interaction between Sema3A and GAGs takes place at Sema3A C-terminal polybasic region (SCT). The aims of this study were to characterize the interaction of the whole Sema3A C-terminal polybasic region (Sema3A 725–771) with GAGs and to investigate the disruption of this interaction by small molecules. Recombinant Sema3A basic domain was produced and we used a combination of biophysical techniques (NMR, SPR, and heparin affinity chromatography) to gain insight into the interaction of the Sema3A C-terminal domain with GAGs. The results demonstrate that SCT is an intrinsically disordered region, which confirms that SCT binds to GAGs and helps to identify the specific residues involved in the interaction. NMR studies, supported by molecular dynamics simulations, show that a new peptoid molecule (CSIC02) may disrupt the interaction between SCT and heparin. Our structural study paves the way toward the design of new molecules targeting these protein–GAG interactions with potential therapeutic applications.

## 1. Introduction

**Figure 1:**
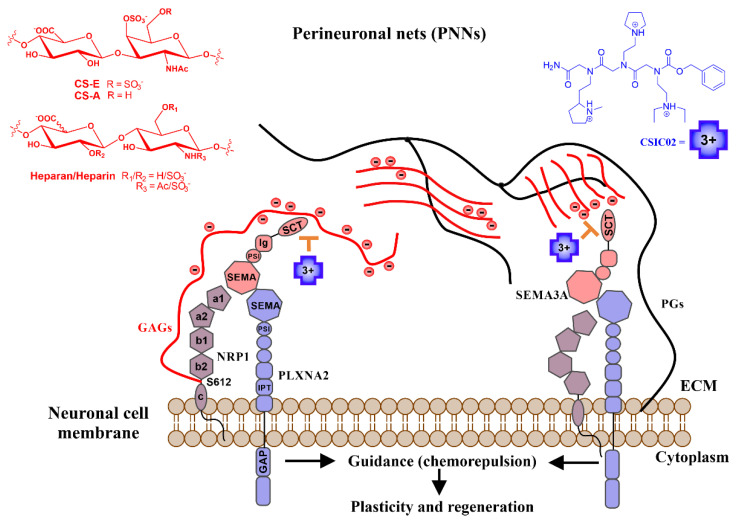
Schematic illustration of the mechanism of action proposed for our small cationic peptidomimetics through the direct inhibition of a biologically relevant Sema3A polybasic C-terminal region (SCT)–GAG interaction. Sema3A forms ternary complexes with its receptor proteins (Nrps-Plxs; Nrp1-PlxnA2 is as example of interaction partners). The figure shows the proposed inhibition mechanism of the Sema3A pathway by interfering the colocalization of secreted Sema3A with its receptor proteins (Nrp1-PlxnA2) due to CSIC02 binding to GAG sites. Long black lines represent the protein part of proteoglycans (PGs), red lines represent negatively charged sugar (GAG) chains. Sema3A/Nrp1/PllnxA2 domains: PSI (plexin-semaphorin-integrin), Ig (immunoglobulin), SCT (basic), IPT (transcription factors), GAP (GTPase-Activating Protein), a1/a2 (CUB), b1/b2 (FV/VIII), and c (MAM).

The extracellular matrix (ECM) is a dynamic three-dimensional network of macromolecules that offers structural support for the cells and tissues and provides a microenvironment that regulates neural cell development and activity [1]. One of the building blocks of these networks are the proteoglycans (PGs). Proteoglycans are constituted by core proteins and one or several attached glycosaminoglycan (GAG) chains. PGs are present in ECM and on the cell membrane surface. GAGs play numerous biological roles mediated by the interaction with a variety of proteins [2,3,4,5]. GAG–protein interactions participate in a variety of human diseases, including cardiovascular diseases, infections, neurodegenerative processes, and tumors [6,7]. Numerous experiments have established that the protein Semaphorin 3A (Sema3A) co-localizes with both cell surface and ECM chondroitin/heparan sulfate proteoglycans (CSPGs/HSPGs) [8,9,10]. Sema3A interacts with CSPGs in perineuronal nets (PNN, one form of ECM in the central nervous system (CNS)), depending, among other features, on the sulfation pattern [10,11,12,13]. Early data showed that heparin enhances Sema3A binding to neuropilin-1-expressing cells and potentiates its growth cone collapsing activity [8]. Sema3A modulates PNN plasticity and the coexistence of both Sema3A and chondroitin sulfate (CS) on the neuronal surface gives stronger plasticity restriction in PNN [13]. These studies support the contribution of CSPGs to the Sema3A-Nrp-Plxn (Nrp = Neuropilin; Plxn = Plexin) signaling complex (Figure 1).

Sema3A is a member of the class-3 secreted Semaphorins. Semaphorins are classified on the basis of the specific structure of their C-terminal domains [14]. Semaphorin class-3 are secreted proteins containing a C-terminal polybasic domain, an Ig-like C2-type (immunoglobulin-like) domain, a PSI domain, and a Sema domain. The Semaphorins receptors, neuropilins and plexins, are expressed in a variety of cell types, including neurons, endothelial cells, and cancer cells. Sema3A was initially characterized as an axon guidance molecule that takes part in regulating the process by which axon growth cones are directed to their appropriate targets during the nervous system growth. In the adult CNS, Sema3A acts as a canonical repulsive axon guidance molecule, inhibiting CNS regenerative axonal growth and sprouting [12]. In addition, Sema3A can also function as chemo-attractive agent, stimulating the growth of apical dendrites [15]. Moreover, class-3 Semaphorins also play a role in many processes outside the nervous system, as tumor progression or suppression [16,17] or in the immune system [18,19] and are attracting much attention as potential therapeutic targets [20,21].

PNNs are involved in memory, plasticity, neuroprotection functions, and abnormal PNNs densities (assembly reduction or degradation) are observed in brain disorders [22]. The enzymatic degradation of CSPGs or destabilization of PNNs has been shown to enhance neuronal activity and plasticity after CNS injury [1]. Class-3 Semaphorins are also present in the neural scar after CNS trauma [23]. As a component of PNNs, Sema3A has a role in ischemic retinopathies and retinal ganglion cells (RGCs) apoptosis in eye diseases [24,25]; thus, Sema3A could be a target for molecular approaches to treat for microvascular disorders in the eye and brain [26,27,28,29,30]. Unlike peripheral nerve cells in other parts of the body, RGCs are part of the body’s CNS, which does not regenerate once damaged. As an example, once RGCs die as a consequence of a pathology as glaucoma, they are not replaced. Because the RGC axon stretches from the retina through the optic nerve to the brain, its projections also become damaged by glaucoma. In addition to the treatments directed at lowering eye pressure, the recovery of retinal ganglion cell dendrites could be an alternative for vision enhancement in glaucoma. Enzymatic digestion of ECM GAGs reduced the effects of elevated pressure on retinal ganglion cell dendritic structure in glaucoma, with moderate dendritic preservation in ocular hypertensive eyes following chondroitinase ABC treatment [31].

As illustrated in Figure 1, class-3 Semaphorins are secreted proteins and their effects are mediated by several receptors (Plexins) and co-receptors (as Neuropilins or GAGs) [32]. Potential applications of Semaphorin/Neuropilin/Plexin targeting as therapeutic approach have been proposed in the literature [20,33]. The antecedents of Sema3A cascade inhibitors are limited, comprising two small-molecule inhibitors, SM-216289 (Xanthofulvin) [34] and SICHI [35], as well as Sema3A-blocking antibodies for inhibition of the signaling pathway [36,37]. Heparin/heparan sulfate–protein interactions play a part in the assembly of multicomponent complexes (as seems the case of Sema3A-Nrp1-Plxn) and are potential targets for therapeutic interventions [38]. It has been suggested that the binding of Sema3A to GAGs on the cell surface or ECM is mediated by the C-terminal polybasic tail [8,39]. In order to confirm and characterize Sema3A–GAG interaction, we explored the binding features of a Sema3A C-terminal construct (725–771, SCT) to GAGs using NMR spectroscopy and surface plasmon resonance (SPR). Our previous findings [35,40], using two peptides from the Sema3A C-terminal region, showed that a small cationic peptidomimetic (SICHI) could interfere with the interaction between the Sema3A C-terminal region and GAGs by displacing Sema3A basic peptides from their interaction with GAGs. We hypothesize that interfering with Sema3A signaling via inhibiting the interaction of Sema3A proteins with GAGs could be an important therapeutic goal for achieving functional recovery after CNS injuries and, in particular, as a neuroprotective approach of the retina against glaucoma [41,42]. For that purpose, we have studied the effect of three new peptoid molecules in the interaction of the whole Sema3A polybasic C-terminal region with GAGs.

## 2. Results

The interest of the Sema3A–GAG interaction as a therapeutic target underscores the importance of the structural characterization of this binding process. The structure of the globular part of Sema3A (Semaphorin and PSI domains) has been previously determined by X-Ray diffraction crystallography [43]. However, to date, there is no experimental structural information with atomic resolution about Sema3A C-terminal polybasic region. Moreover, the development of new small molecule inhibitors of GAG–protein interactions needs a detailed understanding of the role and molecular features of these interactions.

### 2.1. Sema3A C-Terminal Domain Interaction with Glycosaminoglycans (GAGs)

#### 2.1.1. NMR Sequence Specific Assignment and Structural Features

First, sequence specific backbone NMR resonance assignment of the Sema3A polybasic tail (SCT_WT_ 725–771, see sequence in Table S1a) was performed by relying on both proton- and carbon-detected 3D NMR experiments. Although the protein sequence analyzed by NMR is relatively short (47 amino acids), the elevated number of lysine and arginine residues (16 in total) hindered the NMR chemical shift assignment. For this reason, we assigned the backbone resonances of the Sema3A C-terminal region (SCT_WT_) using both triple resonance ^1^H and ^13^C direct detected experiments (Table S2). To acquire the experiments at high resolution without compromising experimental time, we recorded 3D spectra with 25–50% non-uniform sampling (NUS). Side-chain assignments of ^1^H nuclei from Gln, Arg, Lys, Pro, Ile, and Leu residues were obtained by analysis of 3D NMR spectra HCCHCOSY and H(CCCO)NH. The 2D [^13^C, ^15^N]-CON (Figure S2) and 3D ^13^C detected NMR spectra allowed the identification and assignment of the three prolines and the confirmation of specific basic residues chemical shifts. ^13^Cβ Chemical shifts of proline residues are 32.09, 32.14, and 31.99 ppm (Pro14, 18 and 45, respectively), in agreement with a predicted *trans* conformation for prolines [44].

**Figure 2:**
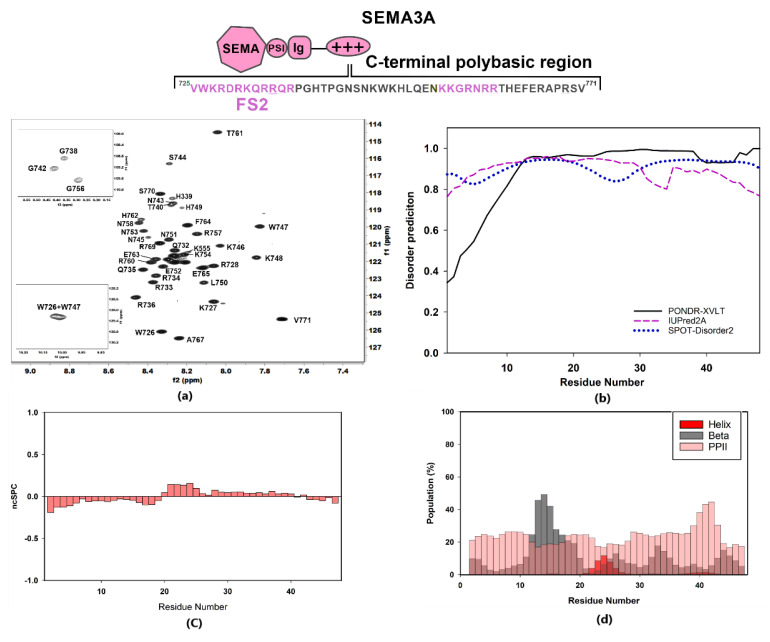
Structural characterization of Sema3A C-terminal domain. The scheme shows the amino acid sequence for Sema3A C-terminal region (SCT_WT_) and the peptides studied in our previous work (labeled in dark rose) [40]. For these Sema3A peptides, we used the terms FS2 and (N)FS3 (for Furin processing sites 2 and 3). (**a**) [^1^H,^15^N]-HSQC spectrum of ^13^C,^15^N-labeled Sema3A C-terminal region at pH 5 (10 mM of acetate and 50 mM of NaCl, 298 K) with the assignment for backbone amides. (**b**) Bioinformatics analysis of the intrinsic disorder predisposition of the Sema3A C-terminal region obtained using IUPred2A, PONDR^®^ VLXT, and SPOT-Disorder2. Disordered segments are indicated by values higher than the default cut-off (0.5), lower values predict structured regions. (**c**) Structural propensity plot using ncSPC (neighbor connected structural propensity plot). (**d**) Estimation of secondary structure populations using δ2D. Red bars indicate helical population estimate, grey bars indicate beta population estimates, dark rose bars indicate PPII population estimates, and remaining white is % of random coil. Chemical shift values for HN, N, C’, Cα, and Cβ nuclei were used for (**c**,**d**) graph calculations.

**Figure 3:**
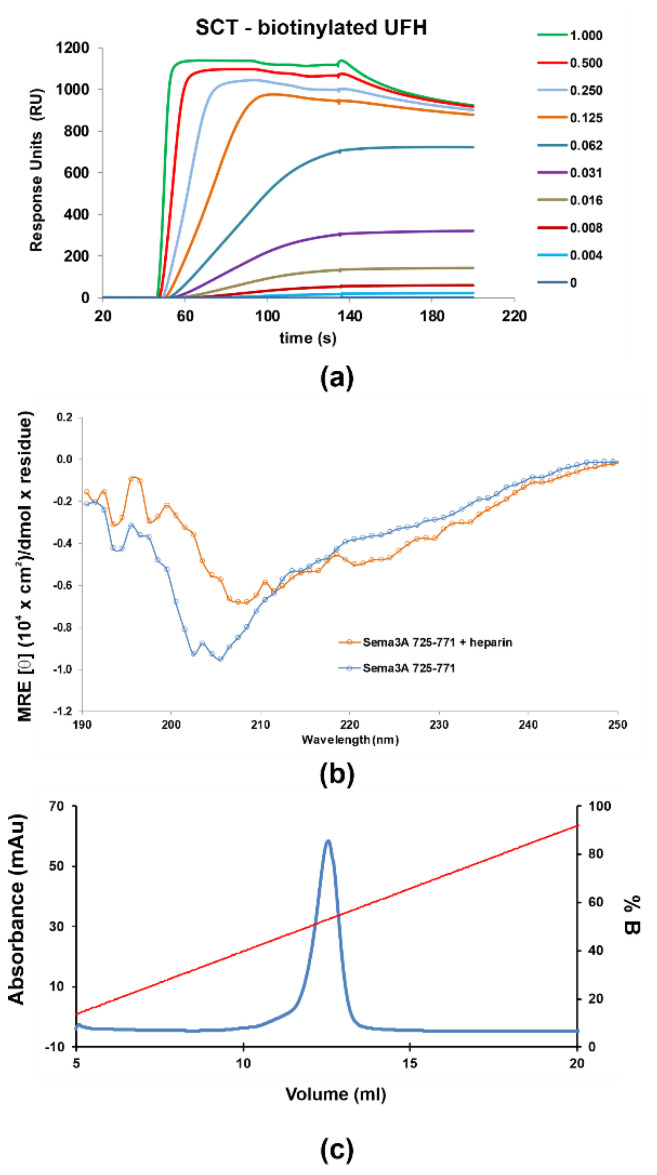
SPR sensorgrams of increasing amounts (0 to 1 μM) of the SCT_WT_ construct flown over (HBS-T buffer) (**a**) immobilized biotinylated heparin (350 RU) (**b**) CD Spectra of 5 μM Sema3A C-terminal region in the absence (blue) and the presence of 1 μM UFH (orange), measured at pH 5.5 in 10 mM NaP. (**c**) Heparin sepharose affinity chromatography profile of SCT_WT_, with a single peak eluting in ~1.06 M NaCl. SCT_WT_ was loaded onto a HiTrap Heparin column equilibrated with 15 mM Tris.HCl (pH 7.5), and eluted with a linear gradient to 2 M NaCl. Left ordinate axis, absorbance; righ ordinate axis, % NaCl (% B).

The ^1^H-^15^N HSQC spectrum of SCT_WT_ at pH 5 and 25 °C showed a narrow amide chemical shift range typical of a disordered region, with ^1^H amide backbone resonances clustering between 7.7 and 8.6 ppm (Figure 2a). Chemical shift NMR assignments were used as input for software ncSPC and secondary structure propensity (SSP) plot calculations [45] (Figure 2c) and software δ2D [46] for population estimates for helical, beta, PPII, and coil (Figure 2d). The data obtained confirmed the disordered nature of Sema3A C-terminal tail. These calculations based on experimental chemical shifts agree with the bioinformatics analysis reported in Figure 2b, which predict that the whole region is fully disordered using three different predictors. Inspection of Figure S3 reveals that the C-terminal part (722–771) of full-length Sema3A is the longest amino acid sequence with a disorder probability of >50%. Additionally, the recently available neural network model, AlphaFold [47], predicts that Sema3A C-terminal tail is disordered, albeit with a low per-residue confidence score. Another experiment confirmed that the Sema3A basic tail is a disordered region near the total disappearance of amide resonances after increasing pH from 4.6 to 6.6 at 298 K in 90:10 *v/v* H_2_O:D_2_O aqueous buffer (Figure S4). Circular dichroism (CD) analysis also confirmed that SCT_WT_ is basically unstructured in solution (Figure 3b). The analysis of SCT_WT_ CD spectrum using DichroWeb server assigns an overall 15% helical content (Figure S5). Although all results indicate that SCT_WT_ is mainly a disordered region, PONDR-XVLT and AlphaFold predictions point to a small amount of helical content in the N-terminal side of the C-terminal polybasic region, in agreement with CD observations. This is consistent with previous studies that reported the presence of a helical motif [48] in Sema3F and Sema3A C-terminal tails, around the cysteine residue (Cys722 for Sema3A). As reported previously, some Semaphorin class-3 proteins (Sema3A, Sema3D, and Sema3F) could form a disulfide bond dimer through the cysteine residue located between the Ig domain and the basic tail (Figure S6) [48,49,50], in addition to non-covalent dimerization of N-terminal Semaphorin domain observed by X-ray diffraction [43]. A preliminary attempt to express and purify a recombinant Sema3A C-terminal construct incorporating the cysteine (LCT_WT_, Table S1a) failed due to the post-translational modification of the N-terminus (Table S1b). Further attempts to express this construct were not explored. It should be noted that, in the ^1^H-^15^N NMR spectra of this construct acquired without and with reducing agent TCEP, an increase in number of resonances and peak dispersion was observed, consistent with the presence of two species (monomer and dimer) in solution (Figure S7).

#### 2.1.2. Characterization of the Interaction between GAGs and Sema3A Basic Tail

The interaction between Sema3A basic tail and GAGs was analyzed using several methods: chromatographic elution as a function of NaCl concentration based on affinity to a heparin sepharose column, surface plasmon resonance (SPR) [51], NMR [52], and molecular dynamics simulations. Firstly, we observed that SCT_WT_ binds to a heparin-Sepharose column and the protein is only released at about 1M NaCl (>50% of buffer B, Figure 3c), similar to the widely reported antithrombin–heparin interaction [53], indicating a SCT_WT_-heparin high-affinity, in the 10^−7^–10^−9^ M range [54]. Next, SPR measurements were performed to characterize protein–heparin binding kinetics. Different dilutions of protein samples were injected in the HBS-EP buffer over a streptavidin chip functionalized with biotinylated heparin or CS-A. The sensorgrams as a function of protein concentration were globally fitted to a site-two stages binding (binding and conformational change) model of the kinetic *k*_on_ and *k*_off_ rates, or a steady-state non-linear fitting analysis was performed using the RU_max_ values for *K*_d(app)_ calculation. The SPR experiments were carried out in duplicate. The sensorgrams approximately fitted to the chosen binding model (Figure S8), but a large disagreement between the two fitting procedures were observed for the protein constructs analyzed (Table S3). Both fitting approaches (kinetics and steady-state) assume an oversimplification of the actual binding; therefore, the obtained apparent *K*_d(app)_ must be used just for comparison purposes. The SPR shapes suggest a complex multi-equilibria model with a fast binding and a slow dissociation processes, which is not well reflected in either of the fitting approaches. Nevertheless, these results may be used as qualitative approximation of SCT–GAG interaction. Our data indicate that SCT_WT_ binds with higher affinity to heparin than to CS-A (Figure 3a and Figure S9). Previous studies, using competitive ELISA and GAG microarrays, showed that full-length Sema3A binds preferentially to heparin and CS-E [10]. The *K*_d(app)_ for SCT_WT_–heparin interaction is in the high-affinity range and matches the affinity assessment from heparin affinity chromatography. Moreover, the other SCT variants analyzed (the mutant SCT_RR/QH_ and the constructs corresponding to the processing of Furin sites 3 and 4 (SCT_FS3_ and SCT_FS4_) show similar affinities for heparin, suggesting low influence of the removed or mutated amino acids. It is evident from these results that the measured affinities are in agreement with the values in the literature for GAG binding proteins [55,56]. On the other hand, when heparin is added into the SCT_WT_ solution, the CD spectrum (Figure 3b) shows a shifting of the minimum and the curve flattening, suggesting some slight conformational change upon heparin binding.

**Figure 4:**
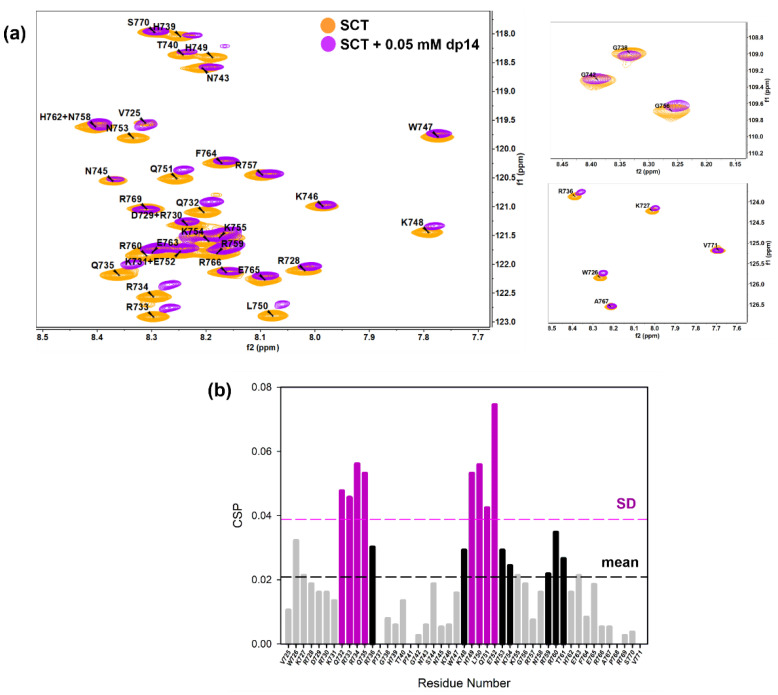
(**a**) Overlay of [^1^H-^15^N]-HSQC spectra of 0.2 mM Sema3A C-terminal region in the absence (orange) and the presence of 0.05 mM (or 0.35 mM in disaccharide units) dp14 heparin oligosaccharide (purple). (**b**) Weighted chemical shift perturbation (CSP) of C-terminal Sema3A region in the presence of dp14 heparin. The absolute values of the chemical shift differences between the presence and absence of dp14 are plotted in ppm and calculated using the weighting factors, described in [57]. Spectra were acquired in 10 mM acetate, 150 mM NaCl, pH 4.5, 10% D_2_O.

Next, NMR chemical shift perturbation (CSP) experiments were carried out to confirm the interaction between SCT_WT_ and GAGs and to identify the corresponding GAG binding sites in Sema3A basic tail (Figure 4). Although both heparin and heparan sulfate are used as equivalents in the literature, only heparan sulfate is naturally present in cells. However, since heparan sulfate led to aggregation with SCT_WT_ (Figure S10), we decided to use heparin dp14 for CSP NMR experiments. We also observed progressive aggregation of SCT_WT_ at higher dp14 heparin concentrations, so we characterized SCT_WT_ CSP upon binding to the GAG at a concentration of dp14 heparin oligosaccharide low enough to avoid sample precipitation.

Figure 4a illustrates the chemical shift perturbation of the backbone amide groups of SCT_WT_ (0.2 mM) in the presence of 0.05 mM (or 0.35 mM in disaccharide units) dp14 heparin oligosaccharide. NMR signals retained low dispersion, showing that the protein remains disordered and flexible in the complex. As shown in Figure 4a, binding to dp14 heparin causes a loss of intensities and significant chemical shift changes in specific regions. Furthermore, we essentially observed a resonance shift upon addition of dp14 heparin, implying a fast exchange on the NMR time scale between the free and the heparin bound states. The chemical shift map (Figure 4b) reveals pronounced chemical shift changes (black line cutoff = CSP mean) for residues in sequences ^732^QRRQR^736^ and ^748^KHLKENKK^755^, while mild changes for residues Trp726 and Arg760. Some of them belong to the typical GAG-binding motif –X_m_BBX_m_- (XBBXBBXBBX, as FS2 peptide ^725^VWKRDRKQRRQR^736^), where B is a basic amino acid and X is a hydrophilic amino acid [58,59]. However, the region corresponding to NFS3 peptide (NKKGRNRR), with the known GAG-binding motif –XBBXBX-, experienced considerably smaller chemical shift variations. This observation is in good agreement with our SPR results using SCT peptides, with a 60-fold stronger affinity to heparin of FS2 peptide compared to NFS3 peptide (Table S3). The second most perturbed site, ^748^KHLKENKK^755^, has a sequence –B_n_-X_2_-B_n_- also commonly observed as a GAG-binding pattern. NMR experiments show that the most perturbed residues are distributed in two adjacent zones and the contribution of the initially predicted GAG-binding region around Furin site 3 ((N)FS3) is smaller. Nevertheless, CSP may not only be triggered by direct binding and other causes, as changes in conformation or chemical environment after heparin binding, could originate the observed changes.

As mentioned above, a SCT construct bearing two mutations, R730Q and R733H (SCT_RR/QH_), the second one lying within one GAG-binding region (FS2), was tested by SPR and heparin affinity chromatography to explore whether these changes affect SCT_RR/QH_-heparin interaction. These mutations have been associated with the Kallman syndrome [60]. Heparin affinity and SPR experiments indicate that the construct still possesses a high capacity to bind heparin, resulting in a slightly earlier elution of the mutated protein whereas the binding to CS-A is completely abrogated (Figures S11 and S12). The truncated constructs of SCT_,_ named SCT_FS3_ and SCT_FS4_ (as they correspond to Furin-processing sites 3 and 4, respectively), were also tested by SPR. Only minor differences in affinity were detected in the interaction of SCT_FS3_ and SCT_FS4_ with heparin compared to the SCT_WT_ (Table S3), which is reasonable given the fact that the positively charged regions on the SCT construct with substantial NMR CSP in presence of dp14 heparin remain intact.

Taken together, our results suggest that both regions identified by NMR may constitute the hotspot for binding to dp14 heparin and the positively charged amino acids around FS3 and FS4 are less important. Conversely, we did not observe any measurable indication of binding between SCT_RR/QH_ and CS-A. Our SPR results show a correlation between the grade of GAG sulfation and the affinity to SCT with tighter binding occurring for higher GAG sulfation. We provide evidence that SCT_WT_–GAG interaction specificity is similar to those found for full-length Sema3A protein, with a preference to bind highly sulfated heparin [10]. In addition, the affinity of the polybasic C-tail for less sulfated CS-A is lower, as demonstrated by Dick et al. [10] for full Sema3A and in contrast with recent studies [61]. SPR and NMR experiments show that the C-terminal domain of Sema3A is a potent heparin binder, allowing us to identify the amino acids responsible for the interaction and hence reinforce the hypothesis that the association of Sema3A to GAGs occurs through the C-terminal polybasic domain.

**Figure 5:**
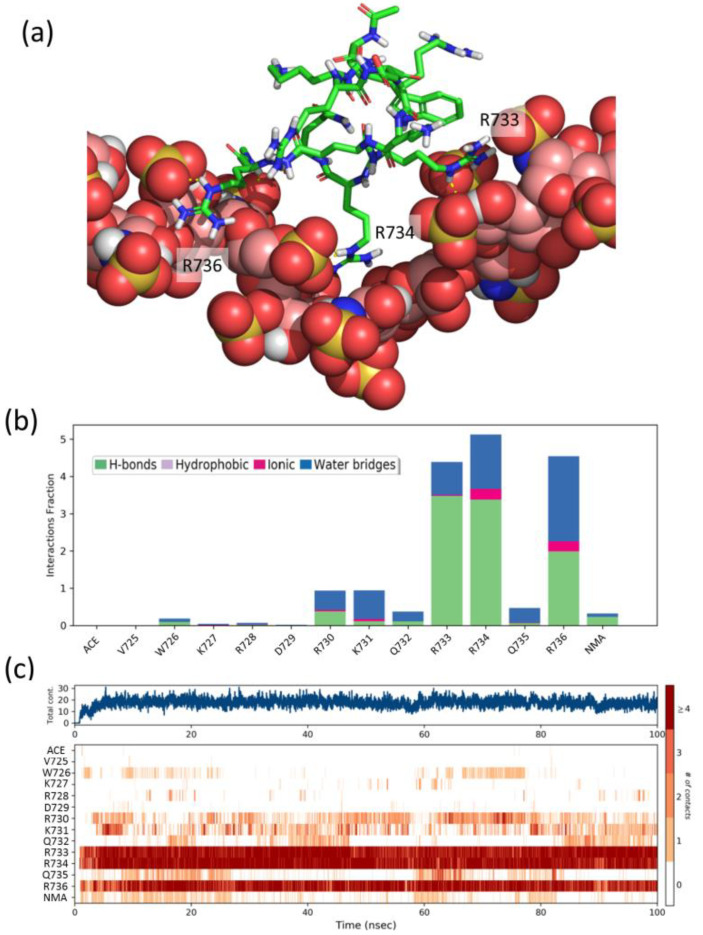
(**a**) Representative snapshot of the heparin/FS2 simulation. Heparin is shown as CPK balls and FS2 as sticks. (**b**) Interactions fraction per FS2 residue. The stacked bar charts are normalized over the course of the trajectory, such that a value of 1.0 suggest that the interaction is maintained over 100% of the simulation time. Values over 1.0 are possible as some residues may establish multiple contacts of same subtype. (**c**) Time dependence of the total number of interactions and of interactions between each residue of peptide FS2 and heparin. Numbering of peptide residues according to Sema3A sequence, ACE, and NMA correspond to acetyl and N-methylamide capping groups of the N- and C-termini.

To gain further insight into the structural details of the SCT-heparin interaction, we performed molecular dynamics simulations of the association of peptides FS2 (^725^VWKRDRKQRRQR^736^), FS3 (^754^KKGRNRR^760^), and Pep1 (^747^WKHLQENKKGRNRRT^761^) with a heparin model. We also performed analogous simulations with two peptides, Pep2 (^738^GHTPGNSNKW^747^) and Pep3 (^762^HEFERAPRSV^771^), which display a CSP below average (Figure 4b) and, thus, were considered as negative controls. Figure 5a shows a representative snapshot of the heparin/FS2 simulation. Analysis of the time dependence of the interactions per residue (Figure 5c) shows that R733, R734, and R736 are the main residues of the peptide-establishing interactions with heparin, during most of the simulation. These interactions are mainly direct hydrogen bonds, although there is also some contribution of water-mediated hydrogen-bonding and ionic interactions (Figure 5b). These residues are among the ones in the SCT_WT_ showing higher CSP values. Similarly, residues K754, K755, R759, and R760 on peptide FS3, and residues K748, Q751, R759, and R760 on Pep1, are the ones that display a higher number of perdurable interactions with the heparin molecule during the simulations (Figures S24 and S25). Some of these are also among those showing CSP values above average. In contrast, residues H749, L750, and E752, which show high CSP, are not found to interact with heparin in our simulations. That may suggest that the observed CSP is more related to effects such as conformational changes or restricted mobility between the bound and free forms of the protein. On the contrary and as expected, peptides Pep2 and Pep3 display a smaller number of contacts and less perdurance of their interactions with heparin (Figures S26 and S27). Therefore, the residues identified in the simulations as more important for the peptides–heparin interaction agree reasonably well with those identified by NMR, thus supporting the importance of these fragments for the interaction between Sema3A SCT_WT_ and heparin.

### 2.2. Analysis of Peptoids Interaction with GAGs

**Figure 6:**
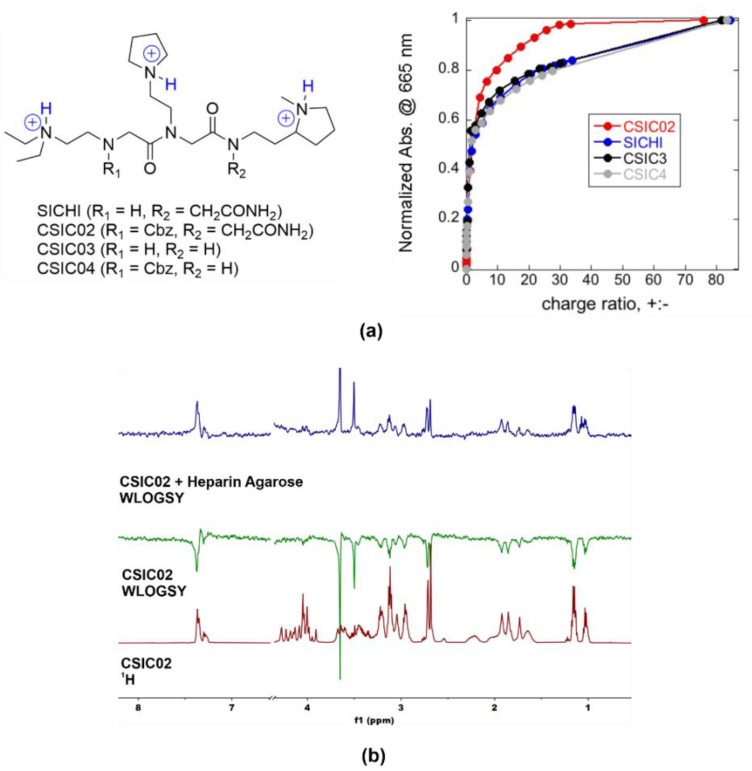
(**a**) Chemical structures of triprotonated SICHI and CSIC02–04, as the main species in aqueous medium at neutral pH, and relative increase in MB absorbance at 665 nm versus charge ratio after the addition of the peptoids to the preformed MB/heparin complex (4.71 µM Hep repeating units + 9.83 µM MB in 5 mM Tris buffer at pH = 7.5). (**b**) 1D ^1^H WaterLOGSY NMR experiments showing that CSIC02 binds to heparin agarose resin (used as a GAG mimetic).

We also designed and synthesized new small molecules targeting protein–GAG interactions with potential therapeutic applications. The molecule called SICHI (Figure 6a) is a synthetic tricationic peptoid that binds GAGs through ionic and H-bonding interactions with the anionic and polar groups of the biopolymer, as previously described by us [35,40]. Considering these results, three more peptoids were prepared (CSIC02, CSIC03, and CSIC04, Figure 6a). Different substitutions at both the N- and C-terminus were considered, through the presence or absence of a Cbz or a 2-acetamide group, respectively. The potentiometric titrations of SICHI and CSIC02 confirmed that both structures are triprotonated at neutral pH. The structural variations in CSIC03 and CSIC04 do not affect the protonation schemes, meaning that the four peptoids would have identical charge distribution in the conditions of the binding studies (Figure 6a).

As a simple way to characterize the GAG binding behavior, we applied the method previously described by Jiao et al. [62], using the cationic dye known as methylene blue (MB) in a colorimetric competition assay. To compare with our previous results for SICHI, we performed similar experimental design and data analysis, using the charge excess at 50% of MB displacement (CE_50_) as the parameter to compare the respective binding abilities. The four peptoids rendered very similar CE_50_ values (CE_50_ = 1.5–1.8, Figure 6a), reflecting the equivalent charge states. However, CSIC02 reached saturation (complete MB displacement) with a lower number of equivalents, which can be due to its slightly larger molecular size that may cause a more efficient interference with MB binding. Next, we assessed the integrity and solubility of CSIC02 by NMR. To characterize its tendency to aggregate in aqueous solution, NMR spectra at increasing concentrations were acquired which show the same sharp signals under all conditions (Figure S13) [63]. Furthermore, the DOSY NMR spectrum showed a single set of signals for CSIC02, indicating a single species in solution and the absence of higher molecular weight aggregates (Figure S13). Direct binding of CSIC02 to GAGs was qualitatively assessed by specific NMR experiments. Figure 6b shows the results obtained using WaterLOGSY experiments that confirm the binding of CSIC02 to heparin agarose. Furthermore, a 1D ^1^H NMR titration of CSIC02 with increasing amounts of chondroitin sulfate (CS from shark cartilage) produces a progressive shifting of CSIC02 resonances (Figure S14), supporting the CS-CSIC02 binding in fast exchange in the NMR chemical shift timescale.

**Figure 7:**
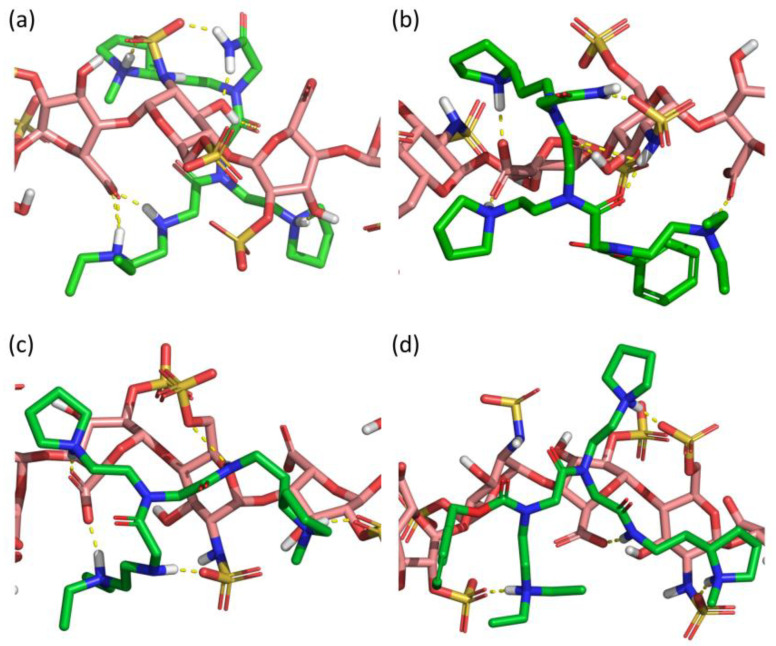
Best docked poses of (**a**) SICHI, (**b**) CSIC02, (**c**) CSIC03 and (**d**) CSIC04 to a dp8 heparin model.

Computational docking of the four peptoids (SICHI and CSIC02 to 04) against a dp8 model of heparin provided a picture of the potential structure of each complex (Figure 7). Similar to the studied peptides, these structures suggest that the four peptoid–heparin complexes are mainly stabilized by hydrogen-bonding and ionic interactions. However, SICHI and CSIC02 can establish a larger number of hydrogen bonds than CSIC03 and CSIC04 due to the presence of their terminal 2-acetamide group. This was also observed for the complexes between the peptoids and chondroitin sulfates A and E (Figures S28 and S29). Further analysis by molecular dynamics supports this observation. Thus, 250-ns simulations of the four peptoid–heparin complexes showed that, despite the complexes being dynamic, the peptoids remain bound to the GAG model during the whole trajectory, browsing the surface of the heparin molecule and establishing/breaking interactions with different groups (Figure S30). The molecular dynamics simulations also suggest that the terminal 2-acetamido group may play a role in the binding of the peptoids to GAGs, since its presence consistently increases the corresponding peptoid–heparin contacts. Accordingly, the binding of SICHI/CSIC02 is more efficient than the interaction with CSIC03–04. However, no large differences were observed between SICHI and CSIC02 (Figure 7 and Figure S30), without notable interactions involving the aromatic ring of the Cbz group of CSIC02. On the other hand, additional steric hindrance in the coverage of the GAG surface and better physicochemical properties make CSIC02 an improved hit for biological applications.

### 2.3. CSIC02 Targets and Modulates the Interaction between Sema3A Basic Tail and Heparin

In our previous work, NMR studies confirmed the interaction between GAGs and peptides FS2/FS3 or SICHI, using a heparin agarose chromatographic resin as a GAG derivative [40]. Furthermore, we implemented competition for NMR experiments between FS2 and FS3 peptides, representing the SCT basic region, and SICHI for binding to the GAG mimetic. In the present work, we have performed similar experiments with the new molecules (CSIC02, CSIC03, and CSIC04) to further investigate the structural determinants in the binding of these peptoids to GAGs.

**Figure 8:**
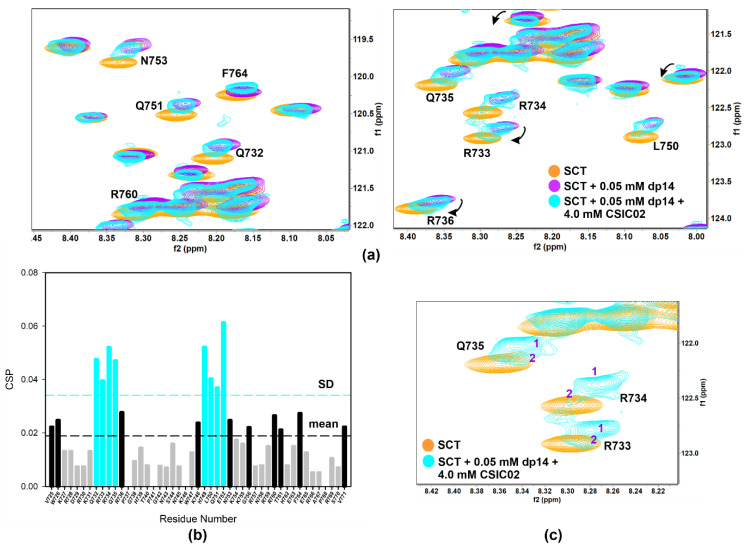
(**a**) Overlay of [^1^H-^15^N]-HSQC spectra of 0.2 mM Sema3A C-terminal region (orange) highlighting the changes of 0.05 mM (or 0.35 mM in disaccharide units) dp14 heparin oligosaccharide binding in the absence (purple) and the presence (turquoise) of 4 mM CSIC02. (**b**) Weighted chemical shift perturbation (CSP) of C-terminal Sema3A region in presence of both dp14 heparin and CSIC02. The absolute values of the chemical shift differences between the presence and absence of dp14 are plotted in ppm and calculated comparing with the chemical shifts of the protein alone. (**c**) Overlay of [^1^H-^15^N]-HSQC spectra of 0.2 mM Sema3A C-terminal region in the absence (orange) and the presence of both 0.05 mM dp14 and 4 mM CSIC02 (turquoise). We observed two species in slow exchange for some peaks (R733, R734, and Q735 in the GAG binding region). Spectra were acquired in 10 mM acetate, 150 mM NaCl, pH 4.5, 10% D_2_O.

NMR competition experiments involved the acquisition of 1D-^1^H WaterLOGSY or spin-lock filtered NMR spectra. Thus, FS2 peptide is displaced from heparin agarose resin by CSIC02 (1:1 peptoid/FS2, both 1 mM), but not by CSIC03 (Figure S15). Moreover, spin-lock filtered 1D-^1^H NMR confirmed that CSIC03 and CSIC04 were incapable to displace FS3 peptide from heparin agarose resin (Figure S16). We reasoned that if CSIC02 was able to interact with GAG and displace FS2/FS3 basic peptides, it may also interfere with SCT_WT_–heparin interaction. To investigate if CSIC02 could displace SCT_WT_ from heparin dp14 and to define the SCT_WT_ residues affected, 2D NMR ^1^H-^15^N HSQC CSP experiments were used. Figure 8a illustrates the chemical shift effects of CSIC02 addition to a sample of SCT_WT_ containing dp14 heparin. To discard direct effects of CSIC02 on the protein chemical shift, a control experiment mixing SCT_WT_ and CSIC02 was acquired which showed that CSIC02 only shifts F764 and V771 amide resonances (Figure S17), and neither of the two residues intervene on the SCT_WT_ binding to GAGs. In Figure 8b, we display the chemical shift perturbation of SCT_WT_ NMR spectra in the presence of dp14 and CSIC02. Thus, as the two CSP plots in Figure 4b and Figure 8b were calculated using as reference SCT_WT_ alone in solution, we can compare them directly. We observed that the addition of a molar excess of CSIC02 caused a modest decrease in dp14-induced perturbations in the HSQC spectrum of SCT_WT_. Moreover, splitting of signals for some residues (R733, R734, and Q735 in the first GAG-binding region) was observed (Figure 8c). This observation is compatible with the presence of different species in slow chemical exchange in solution, with partial detachment of SCT_WT_ from heparin. The amount of CSIC02 used in these experiments can be rationalized considering that the SCT_WT_ has ca. 12 positive charges at neutral pH (Figure S18), while CSIC02 is tricationic. Since the binding is mediated by ionic polar interactions, a large excess of CSIC02 is needed to counterbalance the SCT_WT_-dp14 binding [64].

## 3. Discussion

Our initial attempts focused on identifying the molecular target for the in vitro inhibition of Sema3A chemorepulsive activity exhibited by the small molecule SICHI in growth cone collapse assays showed that neither Sema3A or Nrp1 bind to SICHI [40]. Our findings revealed that SICHI could interfere with the interaction between cationic peptides from Sema3A C-terminal region and GAGs by displacing these Sema3A peptides from the GAGs. This interference may well explain the previously observed SICHI in vitro inhibition of Sema3A pathway [35]. In view of these results, we proposed an alternative therapeutic strategy to target Sema3A signaling complex based on the inhibition of the Sema3A–GAG interaction [40].

Given the biological importance of Sema3A–GAGs interaction, compounds that selectively mimic or inhibit these interactions could be a useful approach to modulate biological processes which are regulated by this interaction, in particular those related to pathogenicity. It should be noted that only class-3 and class-5 Semaphorins bind proteoglycans acting as co-receptors [10,65,66]. Remarkably, class-3 Semaphorins are the only family members with a polybasic domain at the C-terminal end (Figure S1). Several strategies for targeting GAG–protein interactions have been proposed and studied [67,68]. Some of them are more promiscuous (poor tissue selective, no differentiation between normal vs tumor cells), as the enzymatic digestion of GAGs. Others, such as GAGs mimetics, cationic macromolecules (proteins, polymers, dendrimers), or small molecules, could be more selective. Some investigations have reported small molecules as antagonists of heparin or heparan sulfate [69,70]. To effectively design small molecule inhibitor of protein–GAG interactions, structural details about the specific location of the binding/interaction site is highly desirable.

Up to now, there is only indirect evidence of Sema3A C-terminal polybasic region interaction with GAGs, based on which a form of Sema3A (with/without Furin processing) is released after proteolytic digestion of cells ECM GAG chains [8,71]. Clusters of basic amino acids in BBXB and BBBXXB sequences have been proposed as consensus GAG-binding sites in some proteins [72]. Although electrostatic interactions are essential for protein–GAG interactions, polar residues (as asparagine, glutamine, and histidine) may participate in hydrogen bonding with GAGs, and hydrophobic and van der Waals contacts are also important for binding to GAGs. Moreover, the presence of a charged surface or a basic residues cluster does not necessarily mean an efficient GAG-binding epitope. Thus, there was no direct information about Sema3A C-terminal tail structure and which amino acids participate in the binding to GAGs. Prediction software forecasts that Sema3A C-terminal domain is an intrinsically disordered region (IDR). The amino acid sequence characteristics of SCT_WT_, such as low mean hydrophobicity and high net charge, are typical of IDRs [73]. Earlier studies suggested that heparin binding sites may be present in disordered regions [74] and association rates of proteins with heparin were positively correlated with the percentage of disordered residues in heparin-binding sites [75]. In the present work, we have performed a more detailed study of the Sema3A–GAG interaction. Our findings by NMR spectroscopy confirm that Sema3A C-terminal polybasic domain is a disordered region and the most affected residues after heparin binding are two basic clusters, ^732^QRRQR^736^ and ^748^KHLKENKK^755^. The first one contains the BBXB consensus sequence and is part of the peptide FS2 ^725^VWKRDRKQRRQR^736^. The second one may be a region experiencing conformational change after heparin binding to the first site. However, the sequence corresponding to FS3 peptide, which also contains a consensus BBXB-binding motif KKGRNRR, showed appreciable lower chemical shift perturbation in presence of dp14 heparin. As mentioned above, besides groups of positives charges, non-electrostatic interactions can also contribute to the stability of heparin–protein complexes [76,77]. Moreover, these results lend to support that the presence of disorder is not accidental and is very relevant for Sema3A activity modulation.

We have studied the effect of a new peptoid molecule (CSIC02) in the interaction between Sema3A and heparin dp14 oligosaccharide. We observed a displacement of Sema3A basic tail from heparin by CSIC02 using NMR CSP studies. CSIC02, CSIC03, and CSIC04 interact directly with GAGs, but only CSIC02 interferes in the interaction between Sema3A C-terminal peptides and heparin. Complementary analysis combining NMR and molecular dynamics simulations have been used to study the GAG–peptoids complexes, mainly sustained by electrostatic interactions between the anionic groups of the GAGs and the protonated tertiary amines of the peptoids. Our results suggest that SICHI and CSIC02 are able to participate in an additional H-bond via their C-terminal 2-carboxamide residue, whereas CSIC03 and CSIC04 lack this contribution. Besides, the Cbz group plays a marginal role in the GAG–peptoid interaction. On the other hand, CSIC02 has some synthetic and physicochemical advantages over SICHI. Thus, CSIC02 is actually a synthetic precursor of SICHI, with an aromatic residue that simplifies isolation, purification, and UV-detection of the molecule. Besides, the slightly increased hydrophobicity improves the pharmacological properties without compromising aqueous solubility or protonation state at neutral pH, which rules the key electrostatic interaction with the GAGs.

Our structural study supports the feasibility of targeting Sema3A–GAG interactions. The structural information obtained for Sema3A C-terminal region provides a framework for the design of new molecules targeting this protein–GAG interaction with potential therapeutic applications. However, some challenges that lie ahead are the great diversity in the structures of GAGs species and the degree of specificity of a particular GAG–protein interaction. Future work should focus on the affinity and specificity of the small molecule- binders of GAGs, particularly considering their high abundance and the very difficult objective of targeting an individual protein–GAG interaction.

## 4. Materials and Methods

### 4.1. Glycosaminoglycans, Peptoids and Peptides

Glycosaminoglycans were obtained from commercial sources. Unfractionated heparin (Mw~15 kDa), chondroitin sulfate A (CS-A, Mw~15 kDa), and chondroitin sulfate mixture (CS, Mw~50 kDa) were purchased from Sigma-Aldrich (St. Louis, MO, USA) whereas heparin oligosaccharides dp14 (Mw~4.0 kDa) and dp8 (Mw~2.1 kDa), heparan sulfate (HS, Mw~29 kDa), and dermatan sulfate (DS, Mw~41 kDa) were purchased from Iduron (London, UK). Elemental analysis of CS-A from Sigma gave a degree of sulfation per monosaccharide of 0.32 [78]. CS is a mix of different forms of chondroitin sulfate (80% CS-A + CS-C, basic disaccharide unit contain one sulfate) and its overall sulfation degree per monosaccharide is 0.60 [79]. Synthetic peptides from the Sema3A C-terminal domain (FS2, FS3, and (N)FS3; purity > 95%), without any N- or C-terminal modification, were purchased from GenScript USA (Piscataway, NJ). Preparation of SICHI was first described in [35]. Synthesis and characterization of CSIC02, CSIC03, and CSIC04 is detailed in the Supplementary Material.

### 4.2. Recombinant Protein Expression and Purification

SCT recombinant constructs were produced without any fusion tag in *E. coli* BL21 (DE3) with 1 mM IPTG overnight at 37 °C. Purification was achieved by a first step of cationic exchange (SP sepharose HP resin, GE Healthcare, Chicago, IL, USA), performed at pH > 9, followed by reverse phase using a C_18_ symmetry column (Waters) on a ÄKTA purifier system (GE Healthcare). Conditions for cationic exchange were Buffer A: 20 mM Tris-HCl, 130 mM NaCl, 8 M Urea, pH = 9.5; and Buffer B: 20 mM Tris-HCl, 1M NaCl, 8M Urea, pH = 9.5. Eluted protein was dialyzed O/N against milliQ water using a Spectra/Por membrane with a MCWO of 3500 Da in order to get rid of urea and NaCl. Dialyzed protein was then concentrated using Amicon Ultra-15 centrifugal units (Millipore, with a cut-off of 3000 Da) to 5–10 mL. Then, reverse phase chromatography (RPC) was performed to additional purification of the protein (Buffer A, 5% acetonitrile, 0,05% trifluoroacetic acid and Buffer B, 70% acetonitrile, 0,05% trifluoroacetic acid). Protein samples of 1–1.5 mL were injected, and a linear gradient from 0% to 100% B was performed with a flow rate of 1 mL/min (Figure S19). Protein elution was followed by a coupled UV detector by measuring the absorbance at 280 nm (SCT contains two tryptophan residues). The eluted protein was collected and freeze-dried. Purity and integrity of the SCT construct was checked by SDS-PAGE electrophoresis and mass spectrometry (MALDI-TOF) (Figure S20). Protein concentration was determined from Abs_280_ measurements on a NanoDrop 8000 (Thermo Scientific, Loughborough, UK).

The amount of pure protein following this protocol gave modest yields, of about 0.75–1.5 mg/L. Problems arose when attempting to produce isotopically labelled protein (^15^N or ^15^N/^13^C) for NMR studies. Production of labelled protein requires the use of minimal medium (M9), where the sole sources of nitrogen and carbon are added ^15^N NH_4_Cl and ^13^C-glucose. However, M9 medium is not optimal in terms of bacterial growth and protein expression and, consequently, protein yields are generally inferior compared to proteins produced in rich medium (LB). In our case, the attempts to produce labelled protein were unsuccessful and typical strategies to get better protein expression (increased aeration, higher stirring speed, or modifications in the M9 composition) failed to give any improvement. Another strategy is the use of synthetic DNA templates with optimized codon usage; in our particular case, for *E. coli*. Given that the Sema3A C-terminal domain is short, we decided to order a synthetic gene with optimized codons (GeneArt, LifeTechnologies, Carlsbad, CA, USA; Figure S21) and use it as a template for amplification of the different constructs (using specific oligonucleotides for each construct). This approach substantially increased the levels of Sema3A C-terminal domain expression and, hence, the amount of purified unlabelled protein, from 0–1.5 mg/L to 2–12 mg/L (depending on the specific construct) after codon optimization. For the best expressing constructs, we were also able to obtain ^15^N-labeled protein, at around 2 mg/L. Although that could be enough to perform some NMR experiments, we tried to further increase the levels of expression for ^15^N- and ^15^N,^13^C-labelled constructs by using the expression media EnPresso^®^ B Defined Nitrogen-free (BioSilta Oy., Cambridgeshire, UK) and the manufacturer protein expression protocol. In this way, we were able to get yields for labelled Sema3A C-terminal constructs between 5–15 mg/L, even better than the yields for the corresponding unlabelled constructs prepared in LB medium.

### 4.3. Methylene Blue Assay and pKa Determination by Potentiometry

UV absorbance was measured using a SpectraMax M5 spectrophotometer (Molecular Devices, Sunnyvale, CA, USA). The experiments were performed with a 10 μM methylene blue (MB) solution in 5 mM Tris-HCl buffer at pH 7.5, to which increasing amounts of heparin were added to saturation of the MB dye. For the competition assays, solutions of MB and heparin at fixed concentrations were titrated with increasing amounts of SICHI or CSIC02–04 peptoids. Then, the relative increase in absorbance at 665 nm, due to release of MB from the MB–heparin complex when adding either SICHI or CSIC02–04 to the cuvette, can be plotted vs. the charge ratio considering their respective concentrations and that all the peptoids have three positive charges, and heparin has four negative charges per disaccharide unit. With this information, the charge excess for 50% of displacement of the MB dye (CE_50_) for each compound can be calculated for comparison. Potentiometric titrations were carried out in a reaction vessel thermostated with a water bath at 25.0 ± 0.1 °C under nitrogen atmosphere, and 0.15 M NaCl was used as the supporting electrolyte. The titrant was delivered by a precision microburette. The potentiometric measurements were made with a pH-mV meter. The reference electrode was an Ag/AgCl electrode in saturated KCl solution. The acquisition of the emf data was performed with the computer program Tiamo 2.3.1. The HYPERQUAD 2013 program (http://www.hyperquad.co.uk/HQ2013.htm (accessed on 3 September 2021))was used to process the data and calculate the protonation constants. The pH range investigated was 3.5–11.0, and the concentration of CSIC02 was 1 × 10^−3^ M. Two different titrations were performed and fitted either as a single set or as separate curves without significant variations in the values of the stability constants. Finally, the sets of data were merged and treated simultaneously to give the final stability constants (Figures S22 and S23).

### 4.4. NMR Experiments

Next, 1D ^1^H T1*ρ* relaxation filtered and WaterLOGSY NMR experiments were acquired at 25 °C on a 500-MHz spectrometer (Varian Inova; Agilent Technologies, Santa Clara, CA, USA) equipped with an AutoX inverse double-resonance probe with a z-shielded pulsed-field gradient coil. The samples were prepared in a 5-mm Wilmad NMR 528-PP tubes with 10 mM Tris-d11 (for peptide/GAG samples) and 150 mM NaCl dissolved in 90% H_2_O/10% D_2_O at pH 7.5. All spectra were obtained with a sweep width of 8 kHz and 16,384 acquired data points. The experiments were recorded using the standard pulse sequence in the (WLOGSY_ES) Chempack library, with excitation sculpting to suppress water resonances. For Water-LOGSY experiments, the first water-selective 180° pulse was 25 ms long. The spectra were acquired with 1200 transients and a spin-lock pulse of 50 ms at 3.7 kHz. To phase Water-LOGSY spectra correctly, it is necessary to use an internal reference compound. In our case, the reference is the residual ethanol absorbed/bonded in heparin-agarose resin (this interaction was previously confirmed with T1*ρ* filtered experiments), which appears as a positively phased signal. For the T1*ρ* relaxation filtered experiments, we used a proton pulse sequence with a spin-lock filter and excitation sculpting (PROTON_ES, Chempack Library), with 256 transients and a spin-lock pulse of 50 ms at 4 kHz.

Proton and carbon direct detected 2D/3D NMR experiments for protein characterization were acquired at 298 K on a 11.7 T Bruker AVANCE IIIHD spectrometer operating at 500.13 MHz (1H) equipped with a 5-mm cryogenically cooled triple-resonance probehead (TCI). SCT_WT_ protein samples were prepared in Shigemi NMR tubes and the protein concentration for backbone assignment was 1 mM in 10 mM acetate buffer, pH 5, 90%/10% (*v/v*) H_2_0:D_2_O. Important acquisition parameters for 2D/3D protein NMR experiments are reported in Table S2. All applied experiments are implemented in the Bruker Topspin pulse program catalogue and were performed without any additional modification. The 3D experiments were recorded with 25–50% non-uniform sampling (NUS) and multi-dimensional decomposition (MDD) was used for data reconstruction.

All 1D/2D spectra were acquired, processed, and analyzed by using Bruker TopSpin 3.5.6 software and/or MNova 12–14 (Mestrelab Research, Santiago de Compostela, Spain). Chemical shifts were referenced using the ^1^H and ^13^C shifts of DSS. Nitrogen chemical shifts were referenced indirectly using the conversion factor derived from the ratio of NMR frequencies [80]. Three-dimensional spectra were analyzed using CCPNmr Analysis 2.5 [81]. Chemical shift perturbation maps for amide ^1^H and ^15^N resonances were calculated using the Equation [57], where ∆δH and ∆δN are the observed CSP for ^1^H and ^15^N, respectively: Δδ_total_ = {δ_H_^2^ + (0.14 × δ_N_)^2^}^1/2^.

### 4.5. Surface Plasmon Resonance

All SPR experiments were performed on a Biacore T-100 instrument (Cytiva, formally GE Healthcare, Marlborough, MA, USA) in HBS-T buffer (10 mM HEPES, 150 mM NaCl, 0.05% Tween-20, pH 7.5) at 25 °C. Heparin and CS-A were biotinylated at their reducing ends following described procedures [82] and immobilized on a streptavidin (SA) chip. By using heparin concentrations of 1 μM, ~350 RUs and ~450 RUs of biotinylated heparin and CS-A were immobilized, respectively. For binding studies, serial dilutions of peptides or SCT constructs in HBS-T were injected at a flow rate of 60 μL/min, with 60 s and 120 s association and dissociation times for peptides and 90 s and 180 s for SCT constructs. An injection of 2 M MgCl_2_ was included at the end of each cycle to regenerate the sensor surface. Binding responses were double-referenced by subtraction of a buffer injection and of the signal from a control SA channel without any GAG immobilized. Data analysis was performed with the BIAevaluation v1.1 software (Biacore). The sensorgrams as a function of protein concentration were globally fitted to a ‘one site-two stages’ binding (binding and conformational change) model of the kinetic *k*_on_ and *k*_off_ rates or a steady-state non-linear fitting analysis was performed using the RU_max_ values for *K*_d(app)_ calculation. The SPR experiments were performed in duplicate and the dissociation constant (*K*_d_) values and binding levels are expressed as the mean and standard deviation from the individual experiments.

### 4.6. Circular Dichroism

Far-UV CD spectra (260–180 nm) were measured on a Jasco J-815 instrument (Jasco, Tokyo, Japan), using a 1-cm path length cuvette (total volume of 400 mL), with parameters as follows: bandwidth = 1 nm, data pitch = 0.1 nm, and scan speed = 50 nm/min. Proteins were prepared at a 5-mM concentration in 10 mM NaH_2_PO_4_, 150 mM NaF, pH = 6.8. Spectra were processed with the SpectraManager software^TM^ (Jasco) and estimation of protein secondary structure was carried out with the CDSSTR method on the DichroWeb server using a reference set enriched in unstructured proteins [83].

### 4.7. Heparin Agarose Affinity

A prepacked 1-mL HiTrap Heparin HP affinity column (GE Healthcare) was used on a AKTA purifier system to perform heparin affinity chromatography. Then, 100 μL of SCT samples at ~100 μM were injected and run at 1 mL/min in a linear gradient with buffer A = 10 mM Tris-HCl, pH = 7.5 and buffer B = 10 mM Tris-HCl, 2M NaCl, pH = 7.5.

### 4.8. Bioinformatics Tools

To evaluate the intrinsic disorder tendency along the amino acid sequence we used PONDR-VLXT (http://www.pondr.com/ (accessed on 3 September 2021)) [84], IUPred2A (https://iupred2a.elte.hu/ (accessed on 3 September 2021)) [85], and SPOT-disorder2 (https://sparks-lab.org/server/spot-disorder2/ (accessed on 3 September 2021)) [86]. The online tool ncSPC (https://st-protein02.chem.au.dk/ncSPC/ (accessed on 3 September 2021)) was applied to calculate the secondary structure propensity Tamiola and Mulder 2010). The δ2D method (https://www-cohsoftware.ch.cam.ac.uk/ (accessed on 3 September 2021)) [87] was employed to estimate secondary structure populations. Both methods use the chemical shift NMR assignments (HN, N, C’, Cα, and Cβ nuclei) for their calculations.

### 4.9. Computational Methods

All molecular simulations were carried out with the package Schrödinger Suite 2021 [88] through its graphical interface, Maestro [89]. The program Macromodel [90], with its default force field OPLS4 [91] and GB/SA water solvation conditions [92], was used for energy minimization. All molecular systems were automatically assigned the default OPLS4 parameters and partial charges. Molecular docking was performed with the program Glide [93,94,95,96]. Molecular dynamics (MD) simulations were performed with the program Desmond [97,98] using the OPLS4 force field. All MD simulation systems were prepared in the same way. Briefly, solutes were placed in a truncated octahedral box whose sides were at 15 Å of the closest solute atom, with added Cl^−^ or Na^+^ ions to reach neutrality, and the whole system was solvated with TIP3P water using the system builder of the Maestro–Desmond interface [99]. Molecular dynamics were run following a general protocol that started with a default equilibration consisting on: (i) 100 ps Brownian dynamics (periodic boundary conditions (PBC), NVT ensemble) at 10 K, with small time steps (1.0, 1.0, and 3.0 fs, for the bonded van der Waals and short range and long range electrostatic interactions, respectively) and restraints (50.0 kcal/mol) on solute heavy atoms; (ii) 12 ps MD (PBC, NVT) at 10 K, with small time steps and same restraints; (iii) 12 ps MD (PBC, NPT) at 10 K and 1 atm, with default time steps (2.0, 2.0, and 6.0 fs) and same restraints; (iv) 12 ps MD (PBC, NPT) at 300 K and 1 atm, with default time steps and same restraints; and (v) 24 ps MD (PBC, NPT) at 300 K and 1 atm, with default time steps and without restraints. Production MD simulations (2 fs time step) were performed under the same conditions (PBC, NPT, 300 K, and 1 atm) using the Nose–Hoover thermostat method [100,101] with a relaxation time of 1.0 ps and the Martyna–Tobias–Klein barostat method [102] with isotropic coupling and a relaxation time of 2 ps. Integration was carried out with the RESPA integrator [103] using time steps of 2.0, 2.0, and 6.0 fs. A cut-off of 12 Å was applied to van der Waals and short-range electrostatic interactions, while long-range electrostatic interactions were computed using the smooth particle mesh Ewald method with an Ewald tolerance of 10^−9^ [104,105]. Bond lengths to hydrogen atoms were constrained using the Shake algorithm [106]. The simulation interactions diagram (SID) application included in the Desmond–Maestro interface and different ad-hoc scripts were used to analyze the simulations results. In this way, the interactions between peptides or peptoids and heparin were determined and classified in four types: hydrogen bonds, hydrophobic, ionic, and water bridges. The geometric criteria for H-bonding requires: a distance ≤2.5 Å between the donor and acceptor atoms (D—H···A); a donor angle of ≥120° between the donor-hydrogen-acceptor atoms (D—H···A); and an acceptor angle of ≥90° between the hydrogen-acceptor-bonded atom (H···A—X). The hydrophobic contacts fall into three subtypes: π–cation (aromatic and charged groups within 4.5 Å), π–π (two aromatic groups stacked face-to-face or face-to-edge), and other non-specific interactions (two hydrophobic groups within 3.6 Å). Ionic interactions are those between two oppositely charged atoms that are within 3.7 Å of each other and do not involve a hydrogen bond. Water bridges are hydrogen-bonding interactions mediated by a water molecule, but the hydrogen-bond geometry is slightly relaxed from the standard H-bond definition: distance ≤2.8 Å, donor angle ≥110°, and acceptor angle ≥90°. All figures and movies from the docked structures and simulations were prepared with Pymol v. 2.4.1 [107].

A heparin dp30 model was derived from structure 3IRK [108] from the Protein Data Bank [109]. This model included 15 l-IdoA2S-α(1→4)-d-GlcNS6S-α(1→4) disaccharide units and it was modeled with all its sulfate and carboxylate groups ionized, thus the total charge of the molecule was -60. A shorter heparin dp8 model (4 disaccharide units, total charge -16) was built from this by removing an adequate number of disaccharide units. A chondroitin sulfate A dp6 model (total charge -6) was derived from structure PDB 1C4S [110], which included three d-GalNAc4S-β(1→4)-d-GlcA-β(1→3) disaccharide units, and, from this, a chondroitin sulfate E dp6 model (total charge -9) was built, by replacing the 6-OH with sulfate groups in the three d-GalNAc4S subunits.

Peptides FS2, FS3, Pep1, Pep2, and Pep3 were built within Maestro in an extended conformation, with all Lys, Arg, Glu, and Asp residues ionized, and with the N- and C-termini capped with acetyl (ACE) and N-methylamide (NMA) groups, respectively. Their structures were energy minimized and then subjected to 200 ns MD, following the above protocol. The structures of the peptides in the last frame of each simulation were used to prepare new systems where the solute consisted of a peptide molecule plus a dp30 heparin molecule, both arbitrarily separated by a distance of > 20 Å. The final solvated systems (~143,000 atoms) were submitted to 100 ns MD, coordinates were saved every 10 ps, and the trajectories were analyzed, as described above.

The peptoid molecules were also built within Maestro, with their three tertiary amines protonated, and energy minimized before docking against dp8 heparin, dp6 CS-A, and dp6 CS-E. Docking was performed using the default Glide XP precision settings. The best docked peptoid–heparin complexes (i.e., those with the best docking scores) were used to setup simulation systems, as described above. The final solvated systems (~17,000 atoms) were submitted to 250-ns MD simulations, coordinates were saved every 250 ps, and the trajectories were analyzed, as previously described.

## 5. Conclusions

We have characterized the interaction of the whole Sema3A C-terminal polybasic region with GAGs and its inhibition. First, we produced, purified ^15^N,^13^C-labeled basic domain and performed the backbone assignment by acquiring 3D ^1^H and ^13^C direct detected NMR experiments. The limited spectral dispersion, and the lack of defined secondary structure elements, predicted based on experimental chemical shifts, categorizes human Sema3A C-terminal polybasic region as an intrinsically disordered region. Next, we used a combination of biophysical techniques (NMR, SPR, Fluorescence and Heparin Affinity Chromatography) to gain insight into the interaction of the Sema3A C-terminal domain with GAGs. These analyses confirmed that Sema3A C-terminal polybasic region binds to GAGs, preferably to heparin, and allowed us to identify the specific residues involved in the interaction. Last, we studied the effect of a new peptoid molecule (CSIC02) in the ineraction between Sema3A and heparin (dp14 oligosaccharide). We observed a displacement of Sema3A basic tail from heparin by CSIC002 using 2D NMR ^1^H,^15^N-HSQC spectra chemical shift pertubation. Our structural study paves the way toward the design of new molecules targeting these protein-GAG interactions with potential therapeutic applications.

## 6. Patents

Messeguer, A.; Alfonso, I.; Bujons, J.; Pérez, Y.; Corredor, M.; Moure, A.; & Solomon, A. Semaphorin 3A neurodegeneration modulators, compositions and uses thereof. Appl. N: EP19383007. Priority Date: 15 November 2019. Applicants: Spanish Research Council (CSIC), Madrid, Spain, Tel-Aviv University, Tel Aviv-Yafo, Israel.

## Data Availability

The chemical shift values for the ^1^H, ^13^C and ^15^N resonances of the C-terminal polybasic domain of Semaphorin 3A have been deposited in the BioMagResBank (https://bmrb.io/ (accessed on 3 September 2021)) under accession number 50962.

## Supplementary Materials

The following are available online at https://www.mdpi.com/article/10.3390/ph14090906/s1, Figure S1: Amino acid sequence and predicted furin processing sites in Semaphorins; Figure S2: SCT proline connectivity (C’_i-1_-NH_i_) was observable in 2D ^13^C-^15^N CON experiment; Figure S3: Intrinsic disorder prediction for full-length human Sema3A; Figure S4: 2D ^1^H-^15^N HSQC NMR spectra of SCT_WT_ at different pH values; Figure S5: Analysis of the CD spectra; Figure S6: Results of Clustal Omega sequence alignment for class-3 Semaphorins; Figure S7: Superimposed ^1^H,^15^N-HSQC spectra of LCT_WT_ (716–771) construct and heparin affinity chromatography profiles with/without reducing agent; Figure S8: Binding kinetics of SCT_WT_ to immobilized heparin using SPR; Figure S9: Binding SCT_WT_ to immobilized CS-A; Figure S10: Superimposed 2D ^1^H,^15^N-HSQC spectra of SCT_WT_ in presence of high molecular weight heparin sulfate; Figure S11: SPR sensorgrams of SCT_RR/QH_ flown over immobilized biotinylated-heparin or CS-A; Figure S12: Heparin affinity chromatography superimposed profiles of SCT_WT_ vs. SCT_RR/QH_; Figure S13: Concentration-dependent ^1^H NMR and DOSY spectra of CSIC02; Figure S14: CSIC02 spectra with increasing amount of CS; Figure S15: 1D ^1^H WaterLOGSY NMR experiments; Figure S16: Spin-lock filtered ^1^H NMR spectra; Figure S17: Control NMR experiment to evaluate the effect of 4 mM CSIC02 on the [^1^H,^15^N]-HSQC spectrum of SCT_WT_; Figure S18: pH titration curve of SCT_WT_ calculated using ProteinTool; Figure S19: Reverse phase chromatograms of ^15^N and ^13^C labelled SCT_WT_; SDS-PAGE and MALDI-TOF MS of SCT_WT_ construct; Figure S20: SDS-PAGE electrophoresis and Mass spectrometry of SCT_WT_; Figure S21: Sequence of the original amplified Sema3A gene corresponding to the C-terminal polybasic domain; Figure S22: Potentiometric titration of CSIC02; Figure S23: Comparison of the pKa values and distribution of protonated species at different pH for SICHI and CSIC02; Figure S24. Results from the molecular dynamics simulations of the heparin/FS3 interaction; Figure S25: Results from the molecular dynamics simulations of the heparin/Pep1 interaction; Figure S26: Results from the heparin/Pep2 simulation; Figure S27: Results from the heparin/Pep3 simulation; Figure S28. Best docked poses of SICHI, CSIC02–04 to a dp8 CS-A model; Figure S29: Best docked poses of SICHI, CSIC02–04 to a dp8 CS-E model; Figure S30: Results from the heparin/peptoids simulations; Table S1a: Peptides and Semaphorin 3A basic domain (LCT and SCT) constructs used in the present work; Table S1b: Theoretical versus experimental molecular weights obtained for the different Sema3A SCT constructs produced and purified; Table S2: Experimental acquisition parameters used to collect the 2D/3D NMR experiments; Table S3: Results from the kinetic and steady-state analysis of the SPR sensorgrams; Video S1: 20 ns fragment of the FS2/heparin trajectory, Video S2: 20 ns fragment of the FS2/heparin trajectory, Video S3: 20 ns fragment of the Pep1/heparin simulation, Video S4: 20 ns fragment of the SICHI/heparin simulation, Video S5: 20 ns fragment of the CSIC02/heparin simulation, Video S6: 20 ns fragment of the CSIC03/heparin simulation, Video S7: 20 ns fragment of the CSIC04/heparin simulation.
