# Supplementary material for: Semaphorin 3A—Glycosaminoglycans Interaction as Therapeutic Target for Axonal Regeneration
Source: Pharmaceuticals (Basel). 2021 Sep 7;14(9):906. doi: 10.3390/ph14090906 (PMC8465649; doi:10.3390/ph14090906)
Supplement: Supplementary file 1 [file pharmaceuticals-14-00906-s001.zip › pharmaceuticals-1348820-supplementary.pdf]

Name	Residue Numbering	Source	Sequence
**FS2**	725-736	S	VWKRDRKQRRQR
**FS3**	754-760	S	KKGRNRR
**NFS3**	753-760	S	NKKGRNRR
**LCT$_{WT}$**	716-771	R	ANTMDEFCEQVWKRDRKQRRQRPGHTPGNSNKWKHLQENKKGRNRRTHEFE RAPRSV
**SCT$_{WT}$**	725-771	R	AVWKRDRKQRRQRPGHTPGNSNKWKHLQENKKGRNRRTHEFERAPRSV
**SCT$_{RR/QH}$**	725-771	R	AVWKRD**Q**KQ**H**RQRPGHTPGNSNKWKHLQENKKGRNRRTHEFERAPRSV
**SCT$_{FS3}$**	725-760	R	AVWKRDRKQRRQRPGHTPGNSNKWKHLQENKKGRNRR
**SCT$_{FS4}$**	725-769	R	AVWKRDRKQRRQRPGHTPGNSNKWKHLQENKKGRNRRTHEFERAPR

[a] according to Uniprot entry Q14563
[b] Mutations introduced in the Sema3A basic domain constructs are highlighted in red. An extra Ala residue at the N-terminus resulting from the cloning is marked in grey.
[c] S = synthetic, R = recombinant

Table S1a. Peptides and Semaphorin 3A basic domain (LCT and SCT, for long and short C-terminal) constructs used in the present work.

Sema3A construct	Theoretical M_W (Da)	Experimental M_W (MALDI-TOF)(Da)
**SCT$_{WT}$**	5915	5913
**SCT$_{FS3}$**	4605	4605
**SCT$_{FS4}$**	5730	5732
**LCT$_{WT}$**	7013	7055*

Table S1b. Theoretical versus experimental molecular weights obtained for the different Sema3A SCT constructs produced and purified. *A difference of +42-43 Da mass from the theoretical Mw is observed, likely corresponding to acetylation at the N-terminus.

>UniProtKB - Q14563 (SEM3A_HUMAN) – 16 Lys + Arg

MDEFCEQVWKRDRKQRR|QRPGHTPGNSNKWKHLQENKKGRNRR|THEFERAPR|SV

>UniProtKB - Q13214 (SEM3B_HUMAN) – 11 Lys + Arg

ANSLRMCRPQPALQSLPLESRRKGRNRR|THAPEPRAERGPR|SATHW

> **UniProtKB - Q99985 (SEM3C_HUMAN) – 11 Lys + Arg**

INQYCKDTRQQHQQGDESQKMRGDYGKLKALINSRKSR|NRR|NQLPES

> **UniProtKB - O95025 (SEM3D_HUMAN) – 16 Lys + Arg**

LDQYCEQMWHREKR|RQRNKGGPKWKHMQEMKKKRNRRHHR|DLDELPRAVAT

> **UniProtKB - O15041 (SEM3E_HUMAN) – 15 Lys + Arg**

VEEYCEKVWCTDRKRKKLKMSPSKWKYANPQEKKLRSKPEHYRLPR|HTLDS

> **UniProtKB - Q13275 (SEM3F_HUMAN) – 8 Lys + Arg**

IHQYCQGYWRHVPPSPREAPGAPRSPEPQDQKKPRNRR|HHPPDT

> **UniProtKB - Q9NS98 (SEM3G_HUMAN) – 14 Lys + Arg**

VDEYCERVWCRGTTECSGCFRSRSRGKQARGKSWAGLELGKKMKSRVHAEHNRTPR|EVEAT

Figure S1. Amino acid sequence and predicted furin processing sites (red vertical bar) in Semaphorins using ProP v.1.0b ProPeptide Cleavage Site Prediction (Score > 0.48) and literature data. The negatively charged sulfate and carboxyl groups in GAGs mediate interactions predominantly with positively charged lysine and arginine residues in the protein (bold, in blue). Polar residues, usually asparagine, glutamine, and histidine (bold, black), sometimes participate in hydrogen bonding with GAGs.

Experiments	Dimension of acquired data			Spectral width			NS	d1 + aq (s)	% NUS
	t1	t2	t3	f1	f2	f3			
**^{1}H detected**									
**HSQC**	400 (^{15}N)	2048 (^{1}H)		33.2	16.2		8	1.13	50
**HNCO**	128 (^{13}C)	72 (^{15}N)	2048 (^{1}H)	16.0	36.0	16.2	8	1.14	25
**HNCA**	128 (^{13}C)	80 (^{15}N)	1800 (^{1}H)	30.0	36.0	16.2	16	1.22	25
**HN(CO)CA**	140 (^{13}C)	80 (^{15}N)	2048 (^{1}H)	30.0	36.0	16.2	16	1.13	50
**CBCA(CO)NH**	128 (^{13}C)	88 (^{15}N)	2048 (^{1}H)	80.0	36.0	16.2	16	1.10	50
**CBCANH**	128 (^{13}C)	80 (^{15}N)	2048 (^{1}H)	80.0	36.0	16.2	20	1.10	50
**HBHA(CO)NH**	200 (^{1}H)	72 (^{15}N)	2048 (^{1}H)	36.0	16.2	16.0	16	1.13	50
**HCCHCOSY**	200 (^{1}H)	80 (^{13}C)	1600 (^{1}H)	80.0	16.2	16.0	16	1.10	50
**HCCCONH**	200 (^{1}H)	72 (^{15}N)	1600 (^{1}H)	36.0	16.2	16.0	16	1.13	50
**^{13}C detected**									
**CON**	200 (^{13}C)	1024 (^{15}N)							
**CBCACON**	128 (^{13}C)	80 (^{15}N)	1024 (^{13}C)	80.0	47.0	40.0	16	1.10	25
**CBCANCO**	128 (^{13}C)	80 (^{15}N)	1024 (^{13}C)	80.0	47.0	40.0	16	1.10	25

Table S2. Experimental acquisition parameters used to collect the 2D/3D NMR experiments.

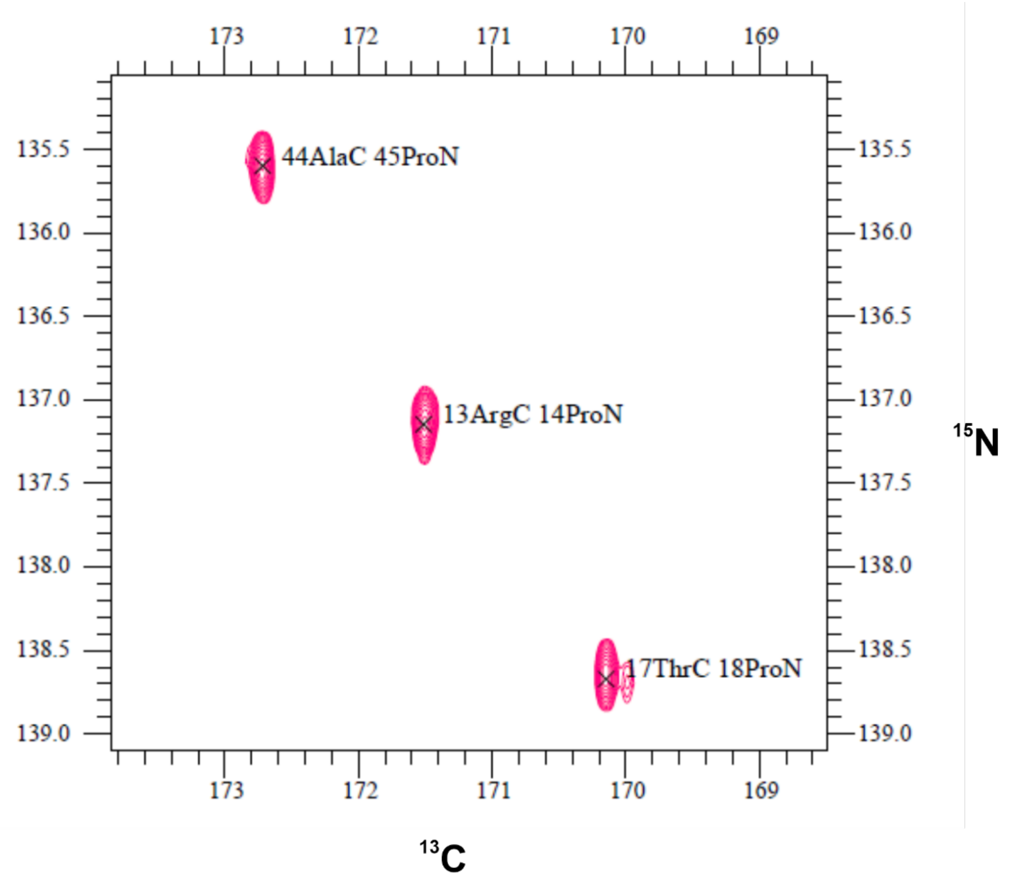

Figure S2. SCT_{WT} proline connectivity (C'_{i-1}-NH_i) was observable in 2D ^{13}C-^{15}N CON experiment.

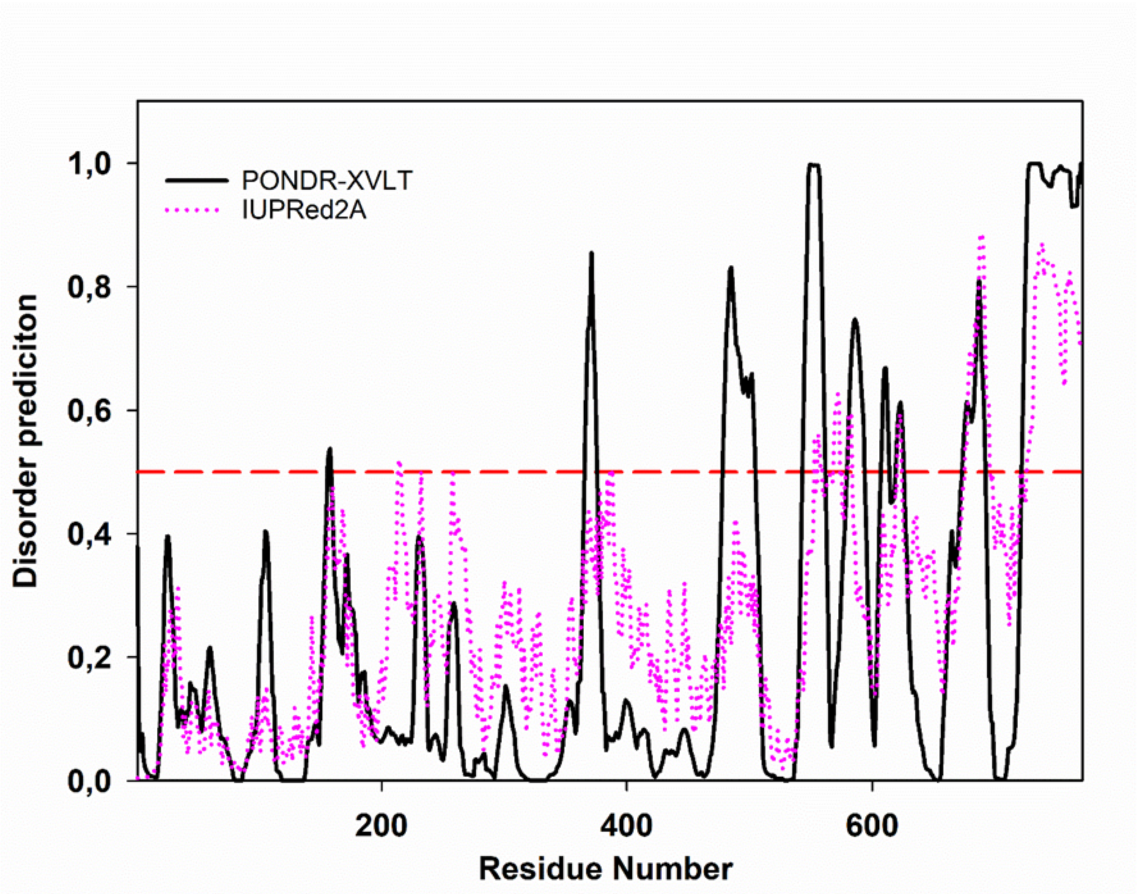

Figure S3. Intrinsic disorder prediction for full-length human Sema3A (UniProt ID: Q141563) using IUPred2A and PONDR® VLXT. Disordered segments are indicated by values higher than the default cut-off (0.5), lower values predict structured regions.

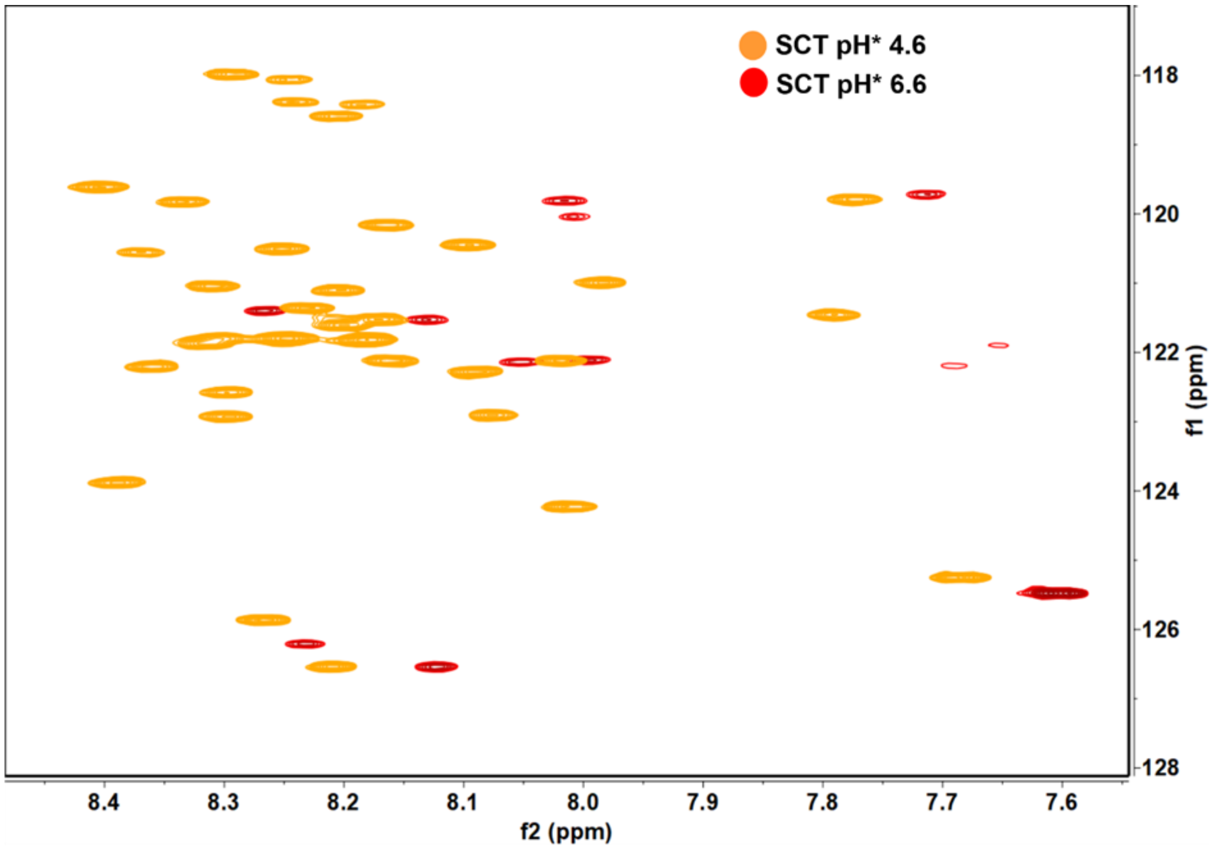

Figure S4. 2D ^{1}H-^{15}N HSQC NMR spectra of SCT_{WT} at different pH values.

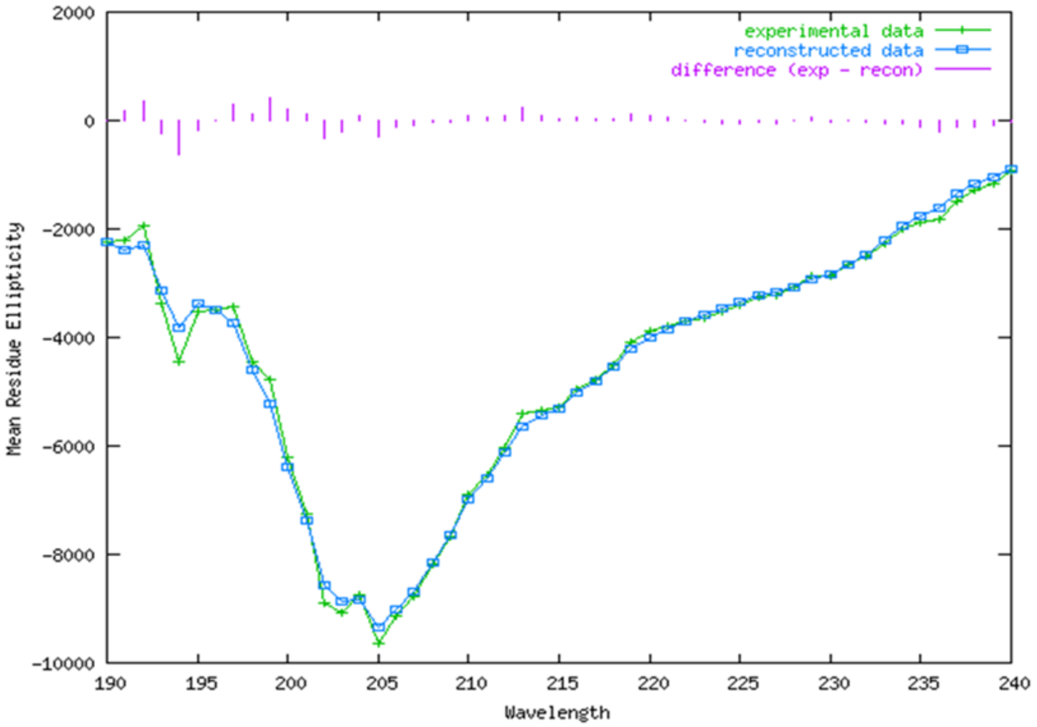

Figure S5. Analysis of the CD spectra using the DichroWeb server assigns a 15% overall helical content for Sema3A C-terminal region (SCT_{WT}), using CDSSTR analysis program with reference set nº 7 (has higher proportion of unfolded proteins). (Whitmore, L.; Wallace, B.A. Protein secondary structure analyses from circular dichroism spectroscopy: methods and reference databases. *Biopolymers* **2008**, *89*, 392-400)

```
sp|Q99985|SEM3C_HUMAN      INQ--YCKDTRQQHQQGDE------SQKMRGDYGKLKALINSRKSRNRRNQLPES----- 751
sp|Q13275|SEM3F_HUMAN      IHQ--YCQGYWRHVPPSPR------EA--PG-APRSPEPQDQKKPRNRRHHPPDT----- 785
sp|Q13214|SEM3B_HUMAN      ANSLRMCRPQ---------------------PALQSLPLESRRKGRNRRTHAPEPRAERG 742
sp|Q14563|SEM3A_HUMAN      MDE--FCEQVWKRDRKQRRQR----PGHTPGNSNKWKHLQENKKGRNRRTHE-FERAPR- 769
sp|O95025|SEM3D_HUMAN      LDQ--YCEQMWHREKRRQRNK----G------GPKWKHMQEMKKKRNRRHHRDLDELPR- 773
sp|O15041|SEM3E_HUMAN      VEE--YCEKVWCTDRKRKKLKM---------SPSKWKYANP-QEKKLR-SKPEHYRLPRH 771
sp|Q9NS98|SEM3G_HUMAN      VDE--YCERVWCRGTTECSGCFRSRSRGKQARGKSWAGLELGKKMKSR-VHAEHNRTPRE 778
                            ..   *.                                  :: : *  :

sp|Q99985|SEM3C_HUMAN      -------      751
sp|Q13275|SEM3F_HUMAN      -------      785
sp|Q13214|SEM3B_HUMAN      PRSATHW      749
sp|Q14563|SEM3A_HUMAN      --SV---      771
sp|O95025|SEM3D_HUMAN      --AVAT-      777
sp|O15041|SEM3E_HUMAN      TLDS---      775
sp|Q9NS98|SEM3G_HUMAN      VEAT---      782
```

Figure S6. Results of Clustal Omega sequence alignment (https://www.ebi.ac.uk/Tools/msa/clustalo/) for class-3 Semaphorins. We only show the C-terminal region.

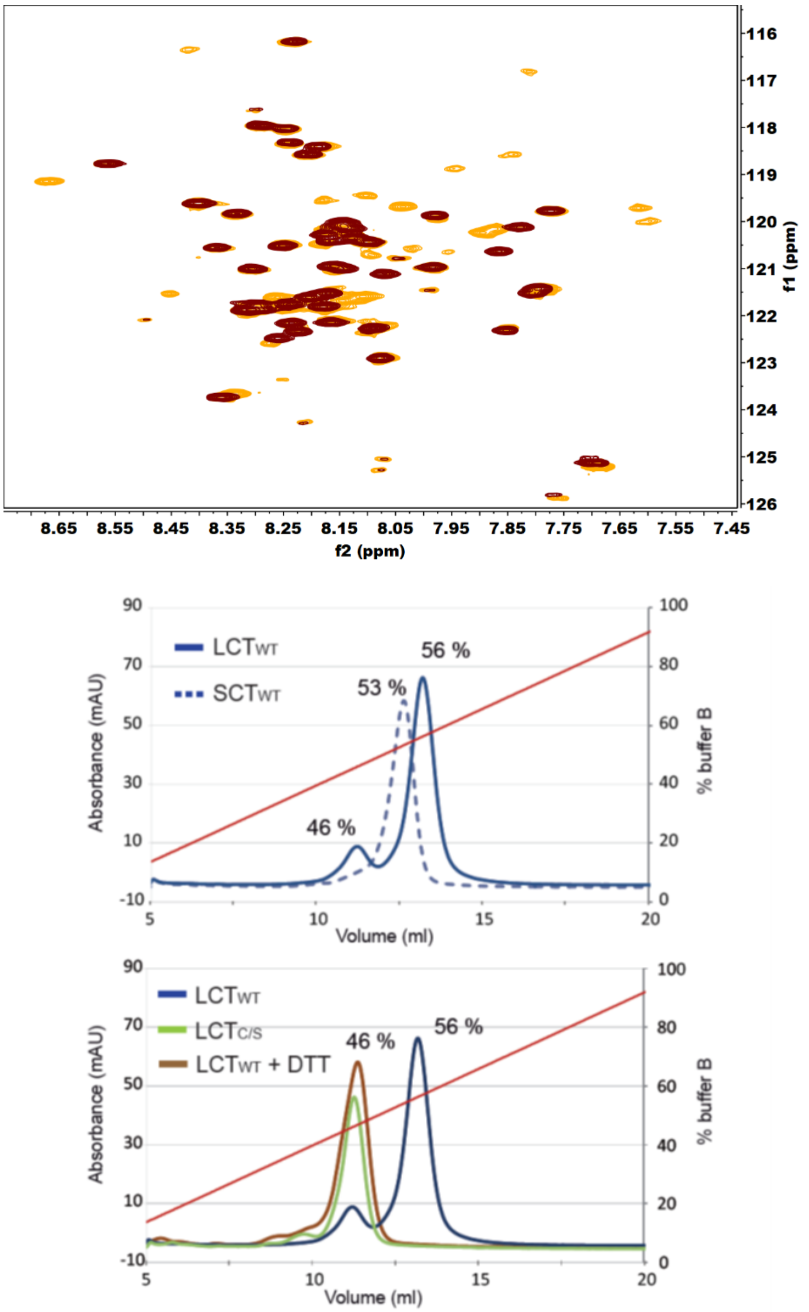

Figure S7. Superimposed ^{1}H,^{15}N-HSQC spectra of LCT_{WT} (716-771) construct without reducing agent (orange) and with 5 mM TCEP (dark red). As we can see from the spectrum, an increase in the number of resonances and peak dispersion in observed in absence of TCEP, consistent with the presence of different species in solution. Heparin affinity chromatography profiles of LCT_{WT} (blue line), with a major peak of elution in ~1.12 M NaCl and a minor peak in ~0.92 M NaCl, and SCT_{WT} (blue dashed line), eluting as a single peak in ~1.06 M NaCl. Heparin affinity chromatography profiles of LCT_{WT} (blue line) vs. $LCT_{C/S}$ (green line) and of LCT_{WT} run under reducing conditions (5 mM DTT, brown line). Both $LCT_{C/S}$ and LCT_{WT} + 5mM DTT elute in ~0.92 M NaCl, matching with the minor peak of LCT_{WT} and confirming that the peak corresponds to the monomeric (no disulfide bond) form of LCT_{WT}.

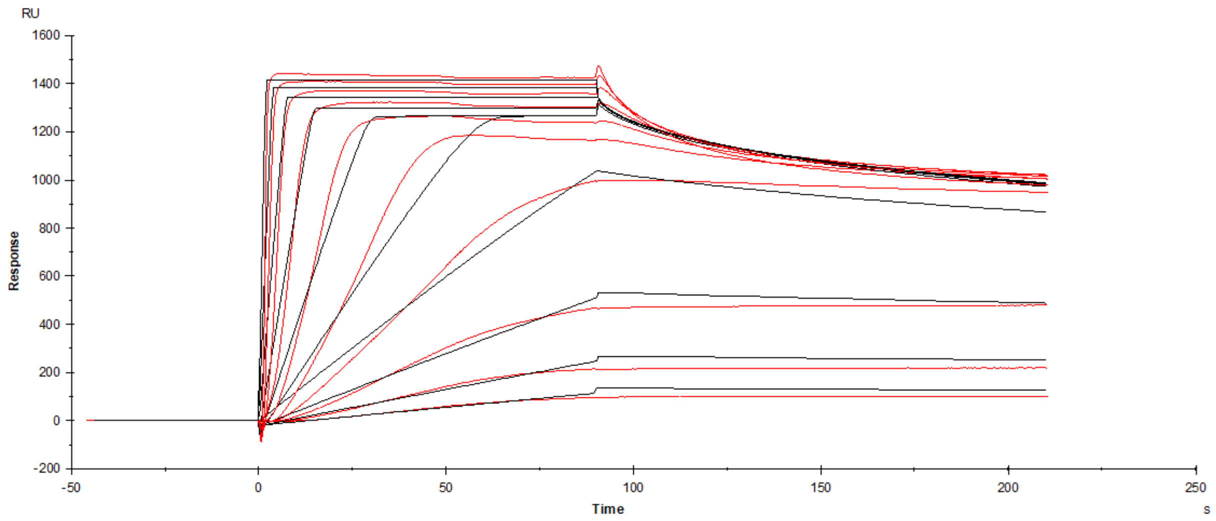

Figure S8. Binding kinetics of SCT_{WT} to immobilized heparin using SPR. The SCT_{WT}/heparin binding kinetics were determined by globally fitting the experimental curves (red lines) to a one site-two stages model (black lines) using the BIA-evaluation software package.

Name	Residues	K_d (kinetic, M)	K_d (steady-state, M)	Comments
**FS2**	725-736	$1.0 \pm 0.1 \times 10^{-6}$	$7.9 \pm 0.9 \times 10^{-6}$	Fast exchange, SS is OK
**FS3**	754-760	6.37×10^{-6}	$5.1 \pm 0.3 \times 10^{-5}$	Fast exchange, SS is OK
**NFS3**	753-760	$5.7 \pm 0.2 \times 10^{-5}$	$1.9 \pm 0.3 \times 10^{-4}$	Fast exchange, SS is OK
**LSCT**	716-771	$0.08\text{-}0.02 \times 10^{-9}$	No fit	No dissociation
**SCT_{WT}**	725-771	$1.6 \pm 0.1 \times 10^{-9}$	$8.9 \pm 1.6 \times 10^{-8}$	Complex sensorgram
**$SCT_{RR/QH}$**	725-771	$7.5 \pm 0.2 \times 10^{-9}$	$1.2 \pm 0.2 \times 10^{-7}$	Complex sensorgram
**SCT_{FS3}**	725-760	$2.2 \pm 0.2 \times 10^{-9}$	$1.2 \pm 0.3 \times 10^{-7}$	Complex sensorgram
**SCT_{FS4}**	725-769	$2.2 \pm 0.2 \times 10^{-9}$	$1.2 \pm 0.2 \times 10^{-7}$	Complex sensorgram

Table S3. Results from the kinetic (one site-two stages model) and steady-state (SS) analysis of the SPR sensorgrams obtained for the interaction between synthetic Sema3A peptides or C-terminal tail recombinant constructs onto heparin-functionalized SPR-chips.

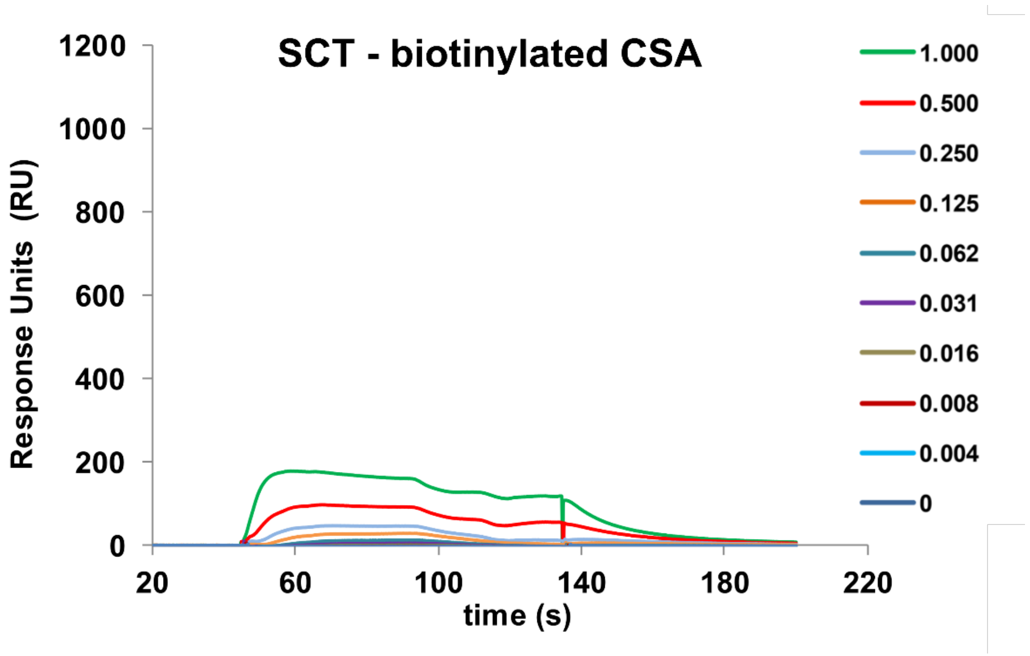

Figure S9. Binding SCT_{WT} to immobilized CS-A (protein concentration from 0 to 1.0 μM).

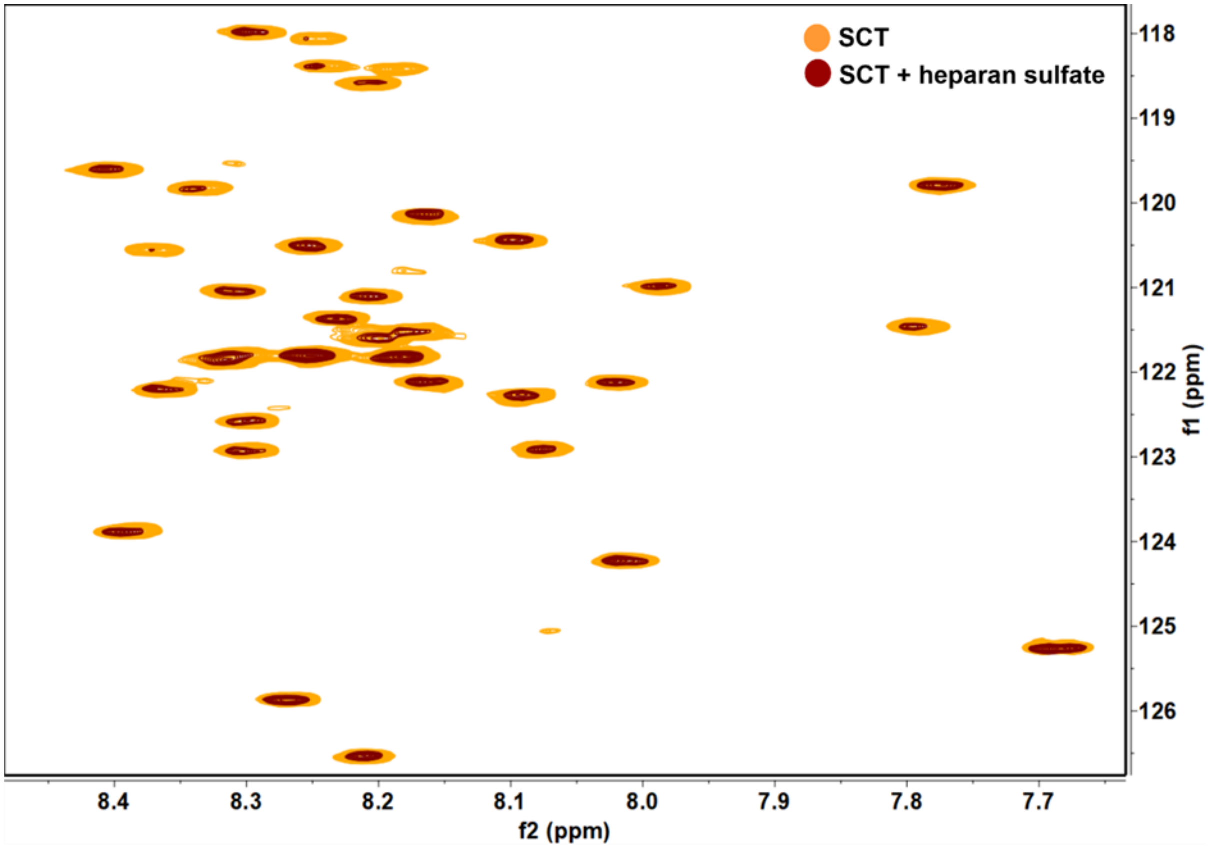

Figure S10. Superimposed 2D $^{1}H,^{15}N$-HSQC spectra of SCT_{WT} in presence of high molecular weight heparan sulfate (0.1 mM protein and 0.08 mM HS, 20 mM acetate, 150 mM NaCl, pH = 4.6, 90/10 H_2O/D_2O).

Sema3A_725-771 R730Q/R733H - heparin

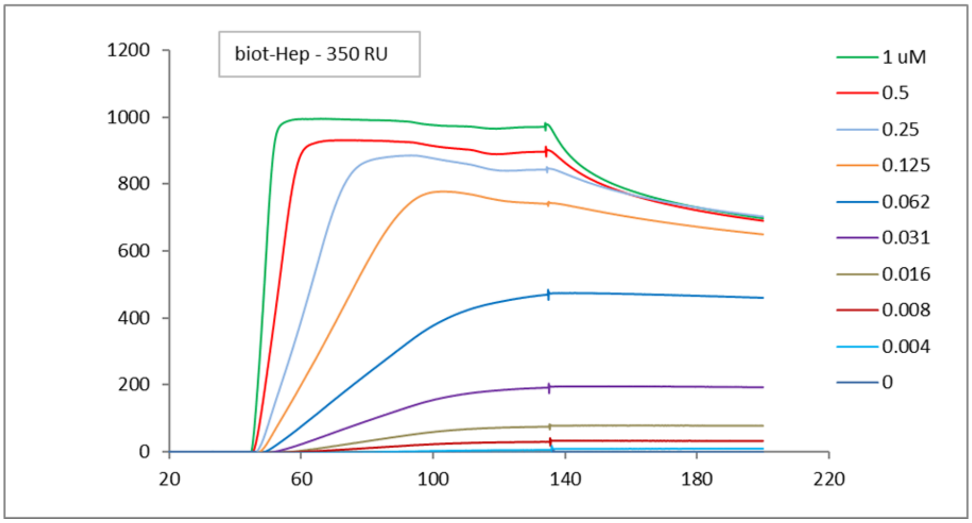

Sema3A_725-771 R730Q/R733H - CSA

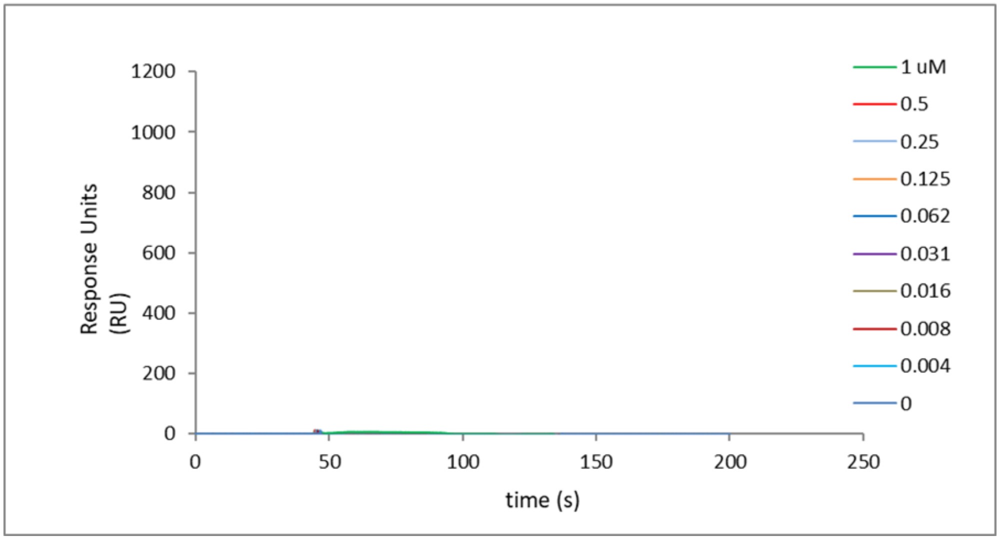

Figure S11. SPR sensorgrams of $SCT_{RR/QH}$ flown over immobilized biotinylated-heparin or CS-A (protein concentration from 0 to 1.0 μM).

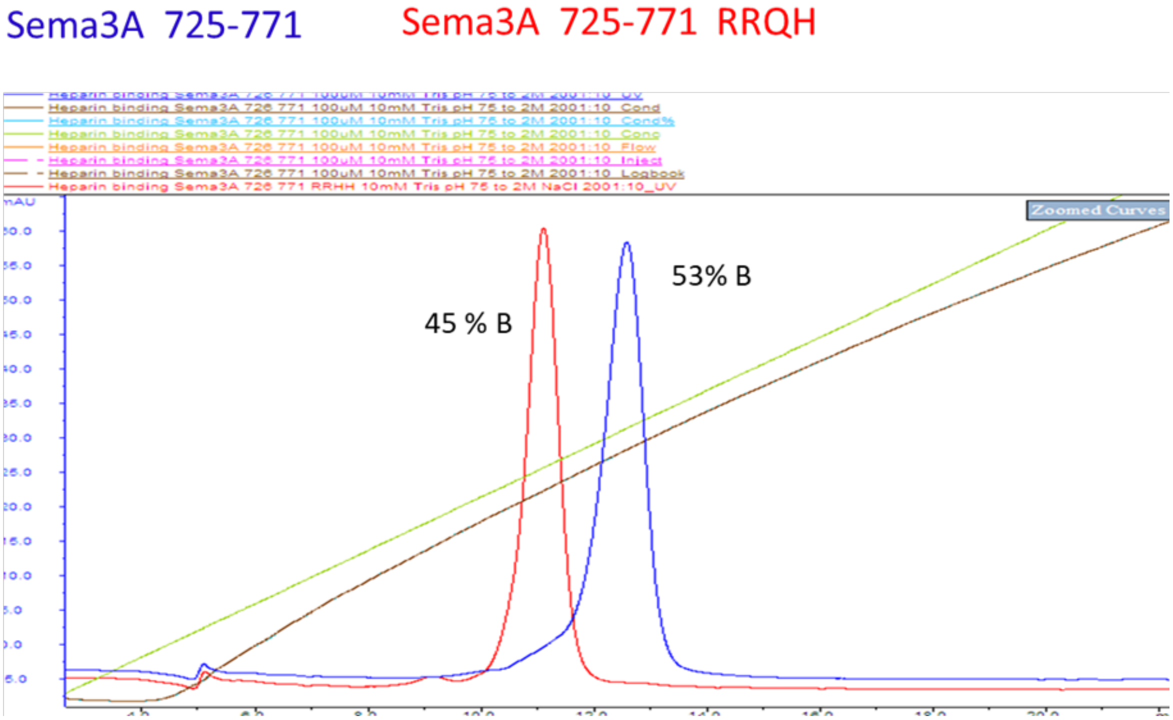

Figure S12. Heparin affinity chromatography superimposed profiles of SCT_{WT} (blue line) vs. $SCT_{RR/QH}$ (red line) with elution peaks in ~1.06 M NaCl and ~0.92 M NaCl respectively.

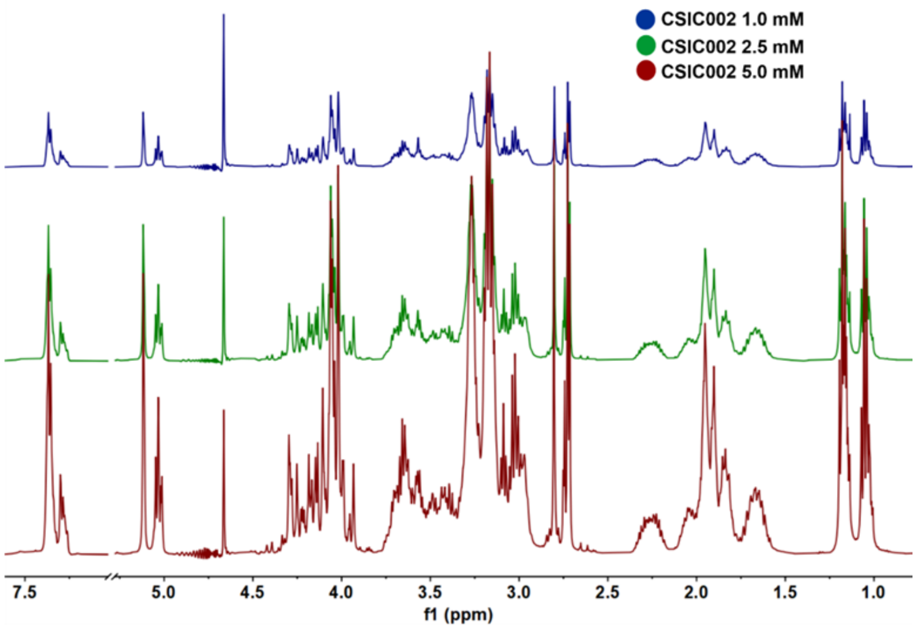

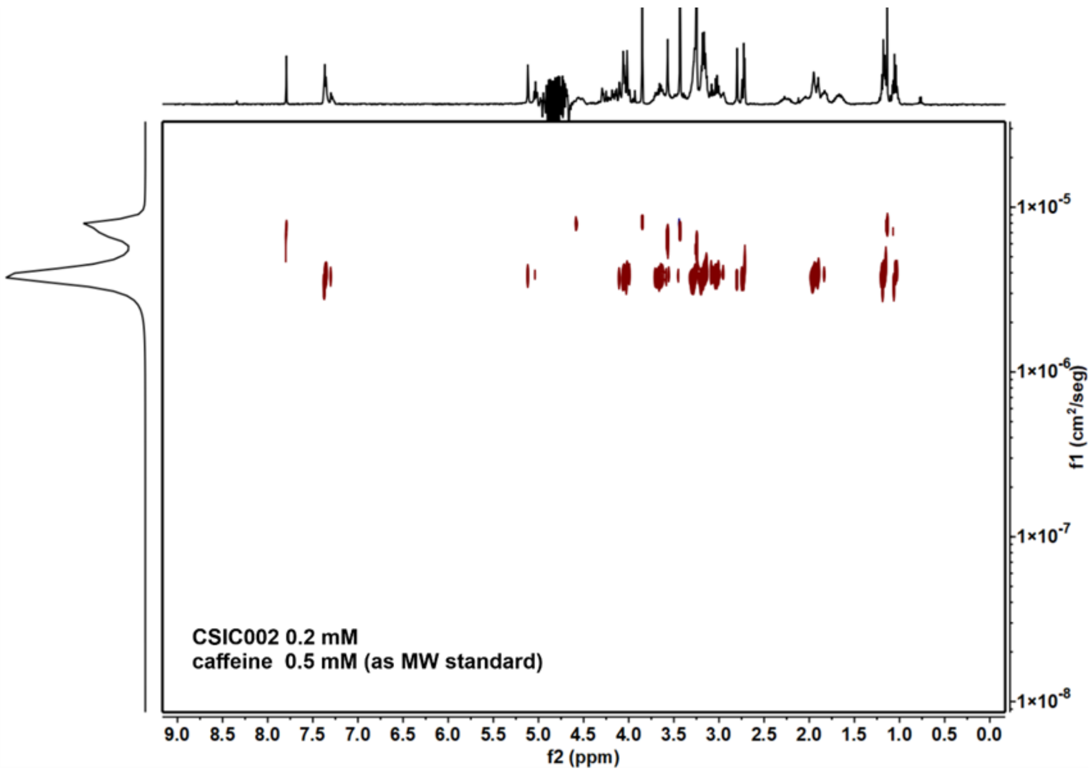

Figure S13. Concentration-dependent NMR spectra of CSIC02 and ^{1}H DOSY NMR spectrum of 0.2 mM CSIC02 in presence of 0.5 mM caffeine. All NMR experiments were done at 298 K in 10 mM Tris-HCl/150 mM NaCl, pH 7.5 in 90% H_2O/10% D_2O. The spectra were processed with MNova software and the measured diffusion coefficients from DOSY were D_{CSIC02}= 3.73 · 10^{-10} m^2/s and $D_{caffeine}$= 7.56 · 10^{-10} m^2/s.

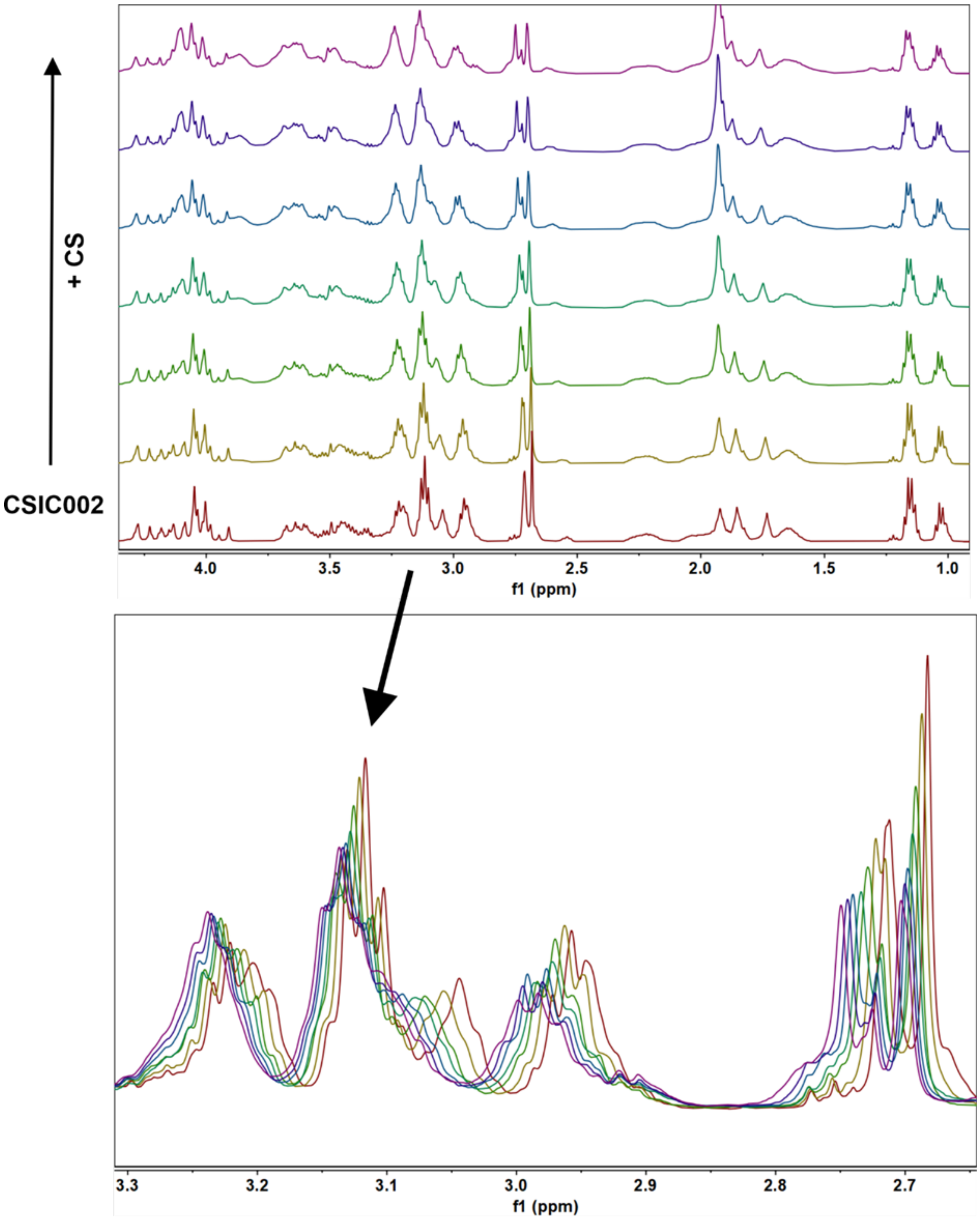

Figure S14. CSIC02 spectra with increasing amount of Chondroitin sulfate from shark cartilage (0,5, 1,0. 1,5, 2,0. 2,5 and 3,0 mg). Spectra were acquired in 10 mM Tris-HCl/150 mM NaCl, pH 7.5 in 90% H_2O/10% D_2O.

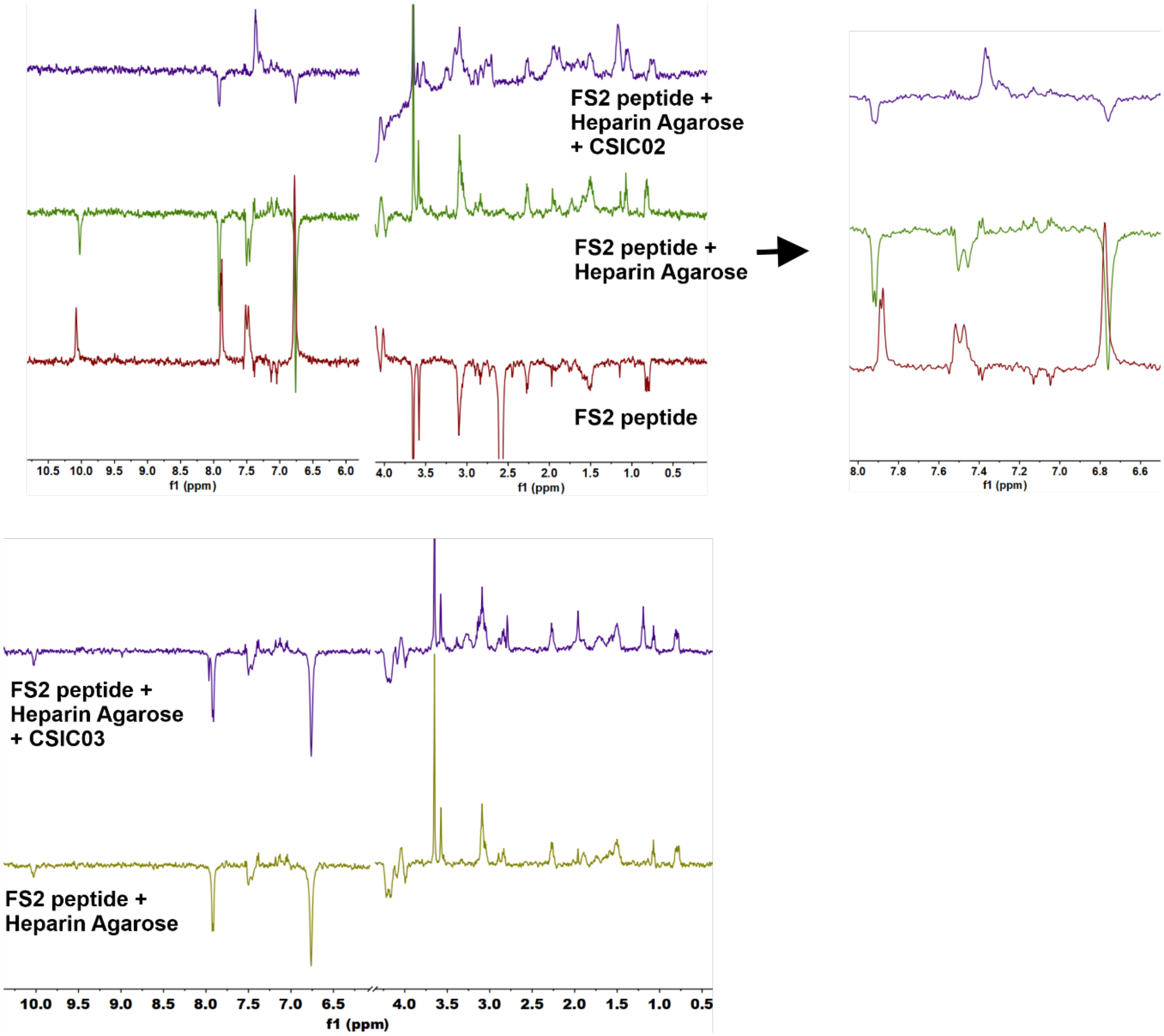

Figure S15. 1D ^{1}H WaterLOGSY NMR experiments showing that only CSIC02 was able to displace FS2 peptide from heparin agarose resin (1:1 peptoid/FS2, both 1 mM). (insert) WaterLOGSY FS2 tryptophan aromatic side chain region. First, peptide protons near water exchangeable protons are affected by water exchange processes and display positive intensity in the WaterLOGSY spectra in absence of heparin agarose. Next, after heparin agarose addition, there is a balance between the cross-relaxation effects between peptide protons and near heparin protons and the cross-relaxation rates between the proton and water molecule and the final result is a change of sign in WaterLOGSY spectrum for tryptophan aromatic resonances. Moreover, competition with CSIC02 decreases the intensity of tryptophan resonances, going towards the initial situation (peptide in absence of heparin agarose) and confirming the competition of CSIC02 with FS2 peptide for the binding to the GAG. (Szczepina, M. G.; Bleile, D. W.; Mullegger, J.; Lewis, A. R.; Pinto, B. M. WaterLOGSY NMR experiments in conjunction with molecular-dynamics simulations identify immobilized water molecules that bridge peptide mimic MDWNMHAA to anticarbohydrate antibody SYA/J6. *Chemistry* **2011**, 17, 11438–11445. https://doi.org/10.1002/chem.201101464)

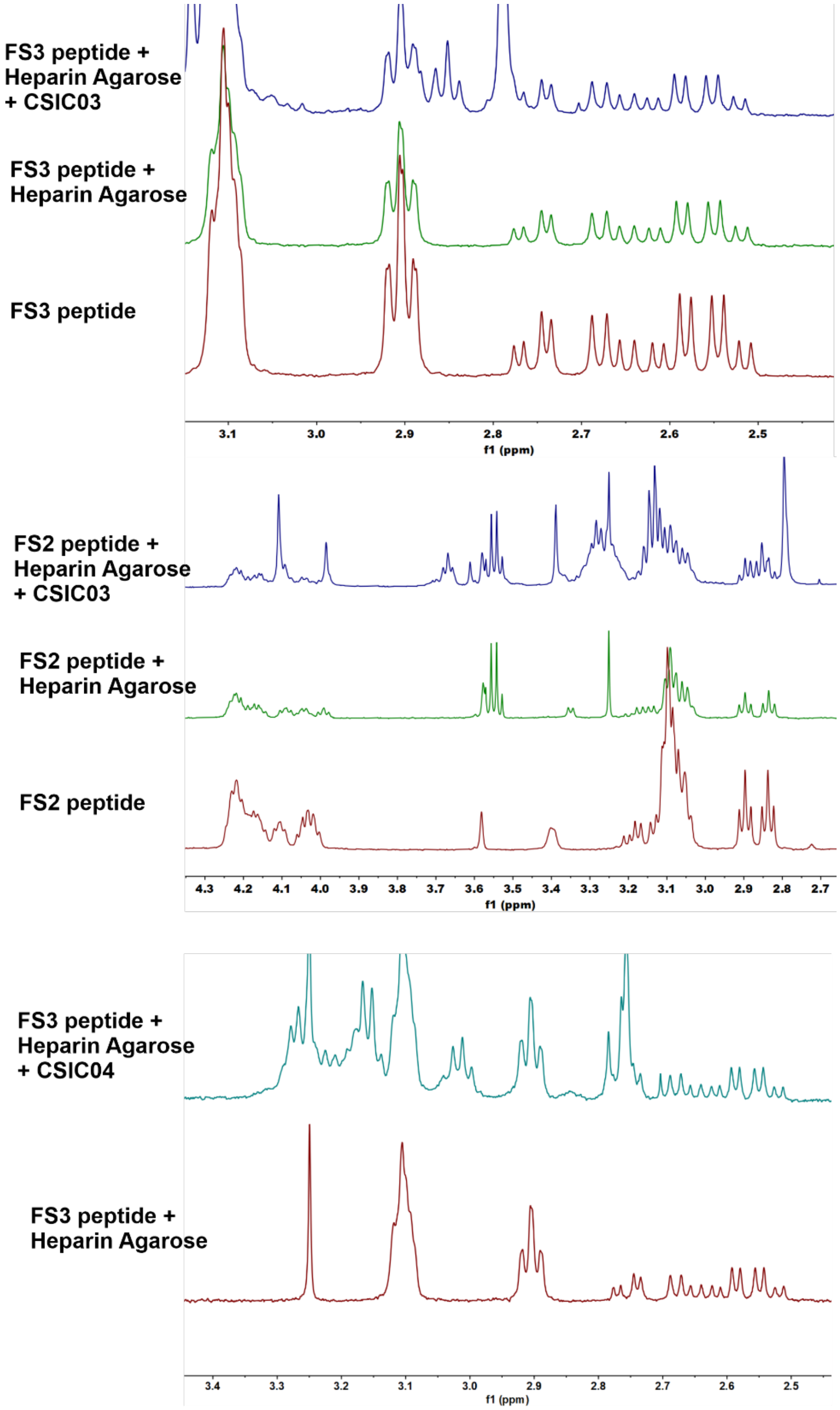

Figure S16. Spin-lock filtered ^{1}H NMR spectra of 1 mM FS3/FS2 peptide alone, with 50 μL suspension of heparin agarose resin and 1 mM CSIC03 or CSIC04. All spectra were acquired in 10 mM Tris-HCl/150 mM NaCl, pH 7.5 in 90% H_2O/10% D_2O.

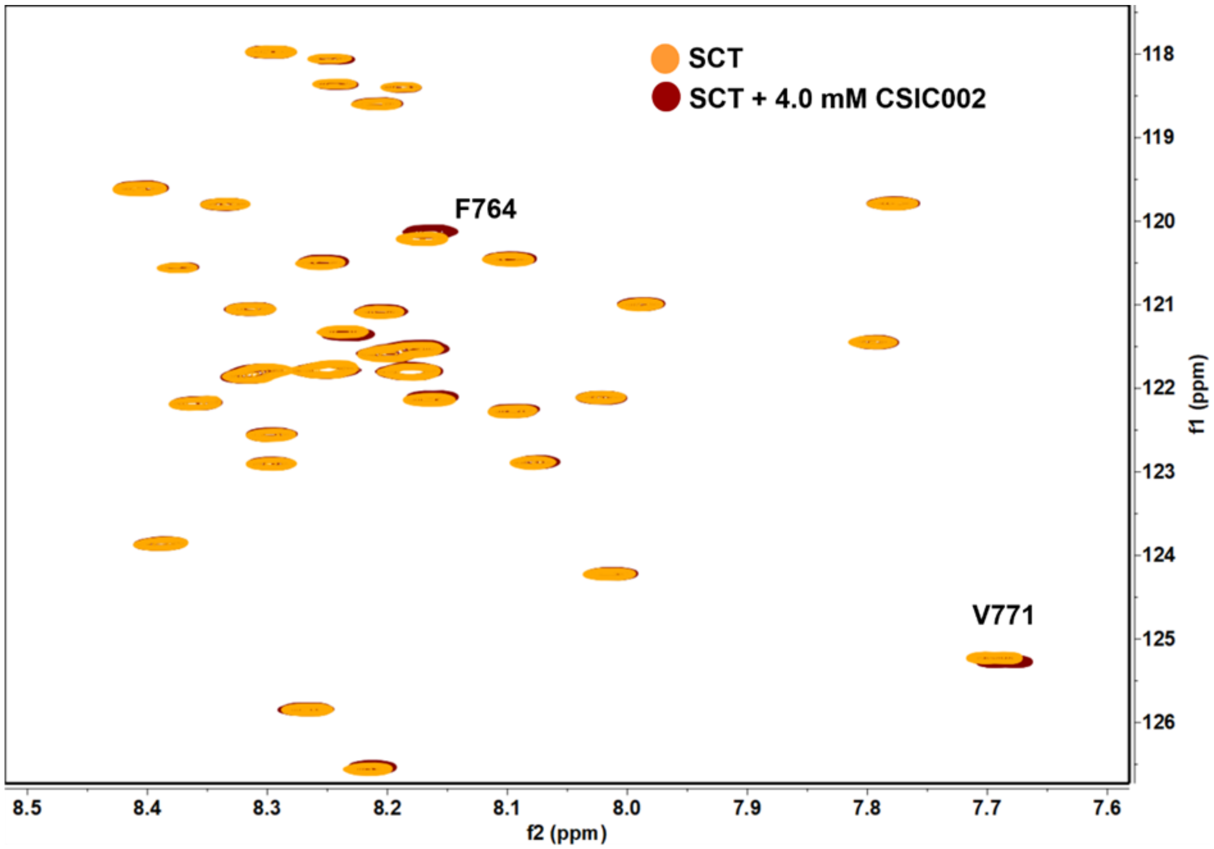

Figure S17. Control NMR experiment to evaluate the effect of 4 mM CSIC02 on the [^{1}H,^{15}N]-HSQC spectrum of SCT_{WT}.

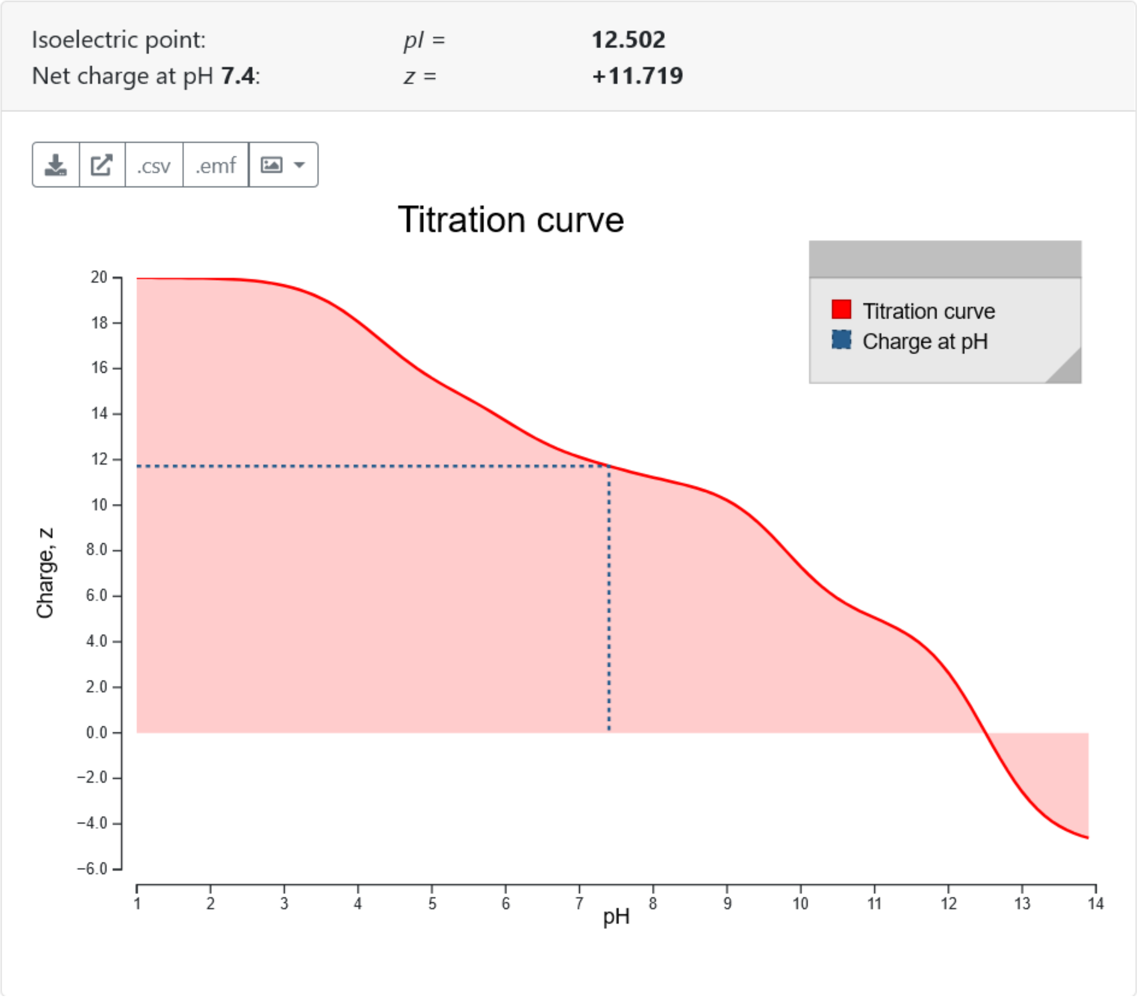

Figure S18. pH titration curve of SCT_{WT} calculated using ProteinTool (at https://www.protpi.ch/Calculator/ProteinTool, Release: 2.2.27.148)

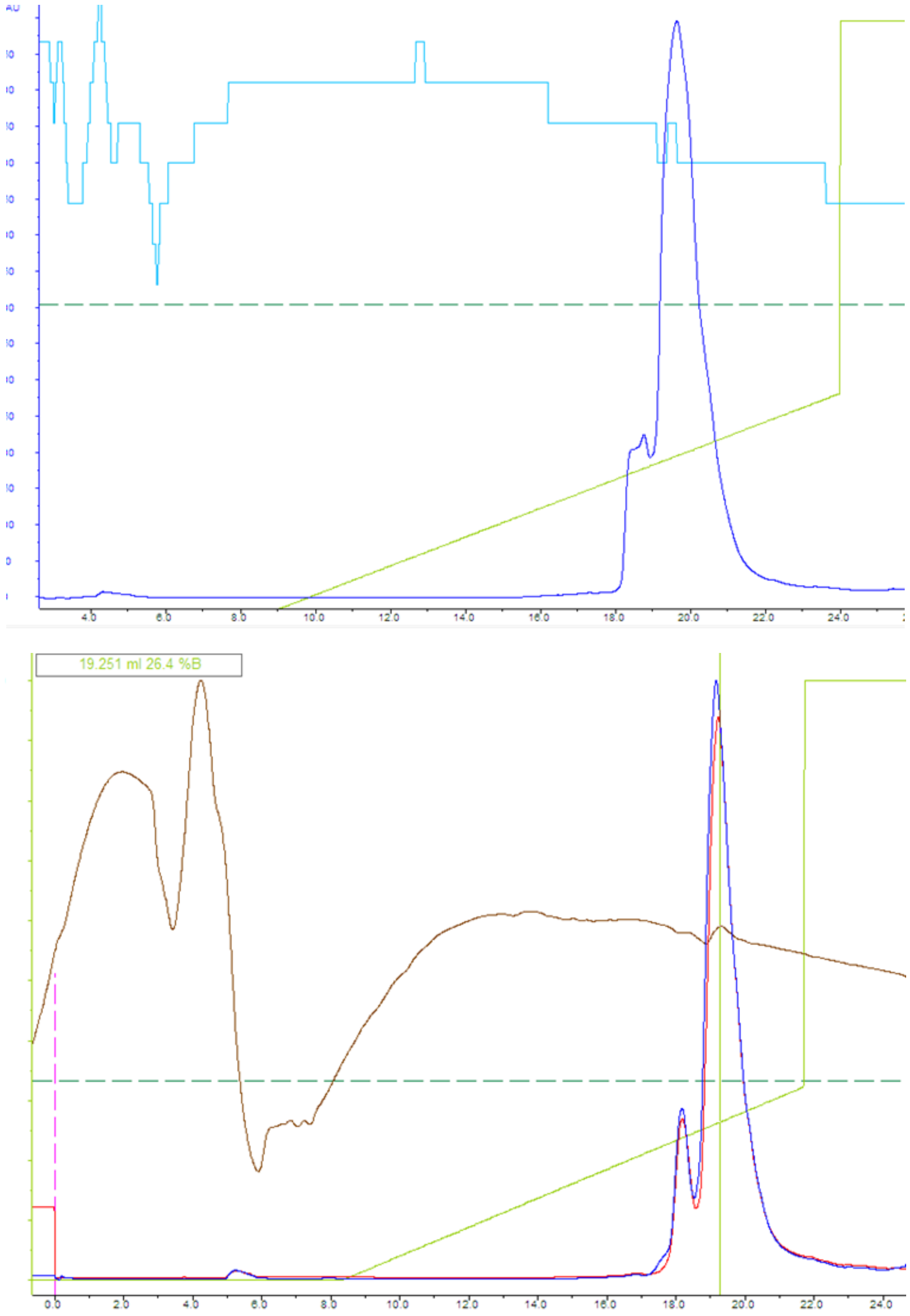

Figure S19. Reverse phase chromatogram of (Top) ^{15}N labelled SCT_{WT} and (Bottom) ^{15}N, ^{13}C labelled SCT_{WT}.

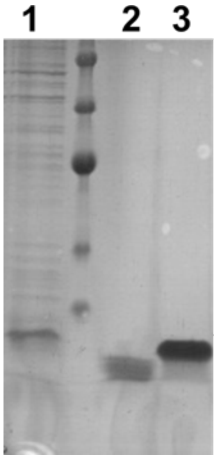

1 E. Coli cell lysate after SCT wt overexpression
2 RPC Peak 1
3 RPC Peak 2

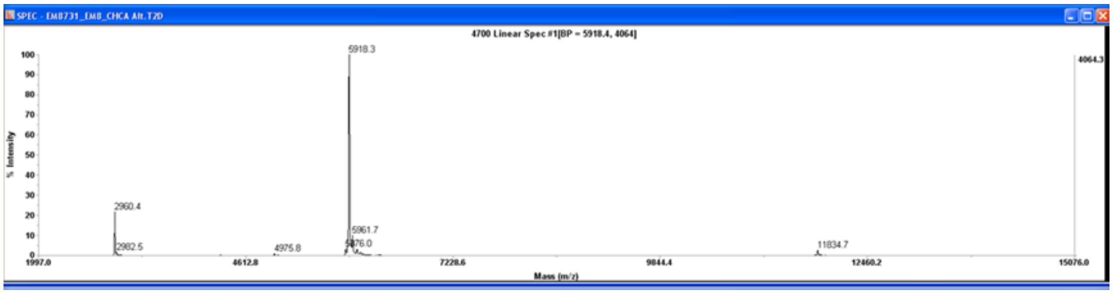

Figure S20. SDS-PAGE electrophoresis and Mass spectrometry characterization (MALDI-TOF, Theoretical M_W 5915 Da) of SCT_{WT} construct (RPC Peak 2).

Sema3A_template

aac acg atg gat gag ttc tgt gaa caa gtt tgg aaa agg gac cga aaa caa cgt cgg caa agg cca gga cat

N T M D E F C E Q V W K R D R K Q R R Q R P G H

acc cca ggg aac agt aac aaa tgg aag cac tta caa gaa aat aag aaa ggt aga aac agg agg acc cac gaa

T P G N S N K W K H L Q E N K K G R N R R T H E

ttt gag agg gca ccc agg agt gtc tga

F E R A P R S V -

Sema3A_template with optimized codons

aat acc atg gat gaa ttt tgt gaa cag gtt tgg aaa cgt gat cgt aaa cag cgt cgt cag cgt ccg ggt cat

N T M D E F C E Q V W K R D R K Q R R Q R P G H

aca ccg ggt aat agc aat aaa tgg aaa cat ctg caa gaa aac aaa aaa ggt cgt aat cgt cgc acc cat gaa

T P G N S N K W K H L Q E N K K G R N R R T H E

ttt gaa cgt gca ccg cgt agc gtt taa

F E R A P R S V -

Figure S21. Sequence of the original amplified Sema3A gene corresponding to the C-terminal polybasic domain. The rare or least used codons by *E.Coli* are indicated in red. *Bottom* – Optimized construct of Sema3A C-t for the expression in *E.Coli*, with the replaced rare codons indicated in green.

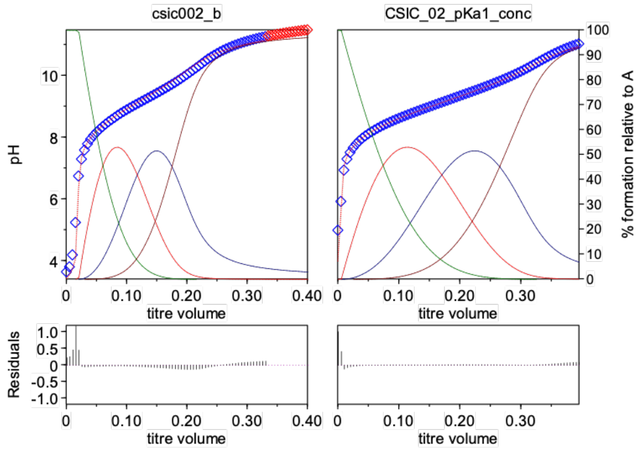

Reaction	Logβ (CSIC02)
$A + H^{+} = A{\cdot}H^{+}$	9.79(2)
$A + 2H^{+} = A{\cdot}H_2^{2+}$	18.90(2)
$A + 3H^{+} = A{\cdot}H_3^{3+}$	27.27(3)

Figure S22: Potentiometric titration of CSIC02 (1 mM in 150 mM NaCl at 298.1 K) showing the fitting performed with Hyperquad 2013, with the cumulative protonation constants obtained as the output (standard deviation on the last significant figure in brackets).

Reaction	pKa (SICHI)	pKa (CSIC02)
$A + H^{+} = A{\cdot}H^{+}$	10.63(2)	9.79(2)
$A{\cdot}H^{+} + H^{+} = A{\cdot}H_2^{2+}$	9.30(4)	9.11(2)
$A{\cdot}H_2^{2+} + H^{+} = A{\cdot}H_3^{3+}$	8.60(4)	8.37(5)
$A{\cdot}H_3^{3+} + H^{+} = A{\cdot}H_4^{4+}$	4.25(6)	n.a.

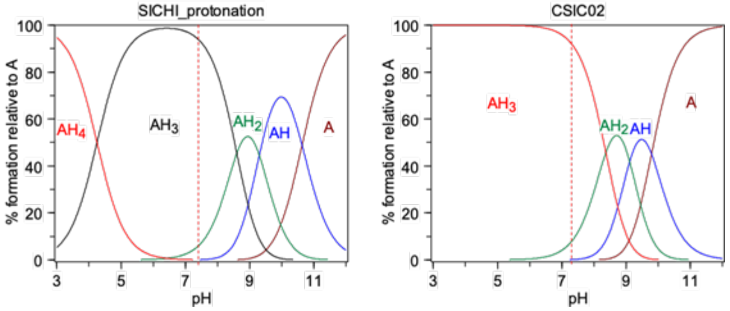

Figure S23: Comparison of the pKa values and distribution of protonated species at different pH for SICHI and CSIC02.

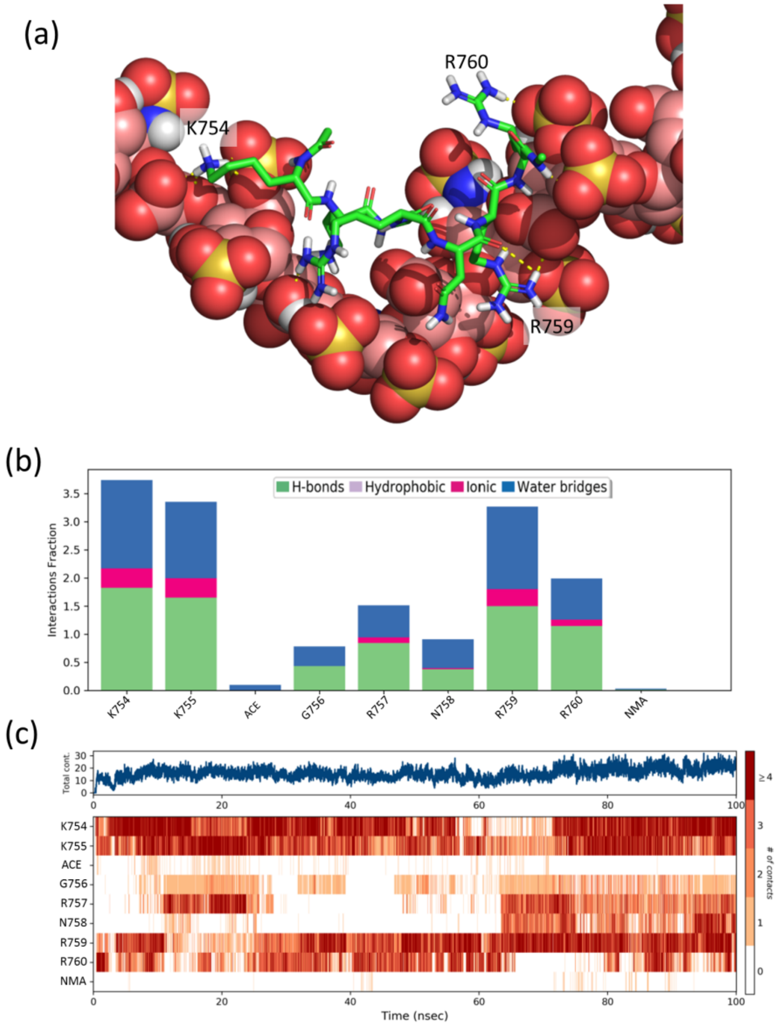

Figure S24. Results from the molecular dynamics simulations of the heparin/FS3 interaction: (a) Representative snapshot of the heparin/FS3 simulation. Heparin is shown as CPK balls and FS3 as sticks. (b) Interactions fraction per FS3 residue. (c) Time-dependence of the total number of interactions and of interactions between each residue of peptide FS3 and heparin.

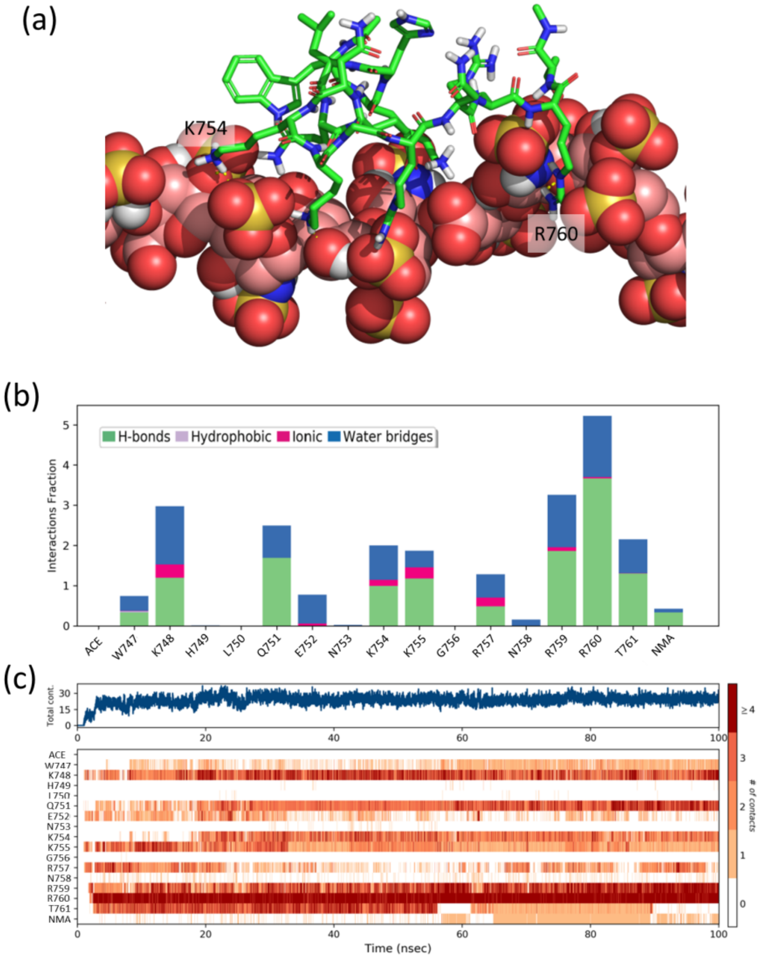

Figure S25. Results from the molecular dynamics simulations of the heparin/Pep1 interaction: (a) Representative snapshot of the heparin/Pep1 simulation. Heparin is shown as CPK balls and FS3 as sticks. (b) Interactions fraction per Pep1 residue. (c) Time-dependence of the total number of interactions and of interactions between each residue of peptide Pep1 and heparin.

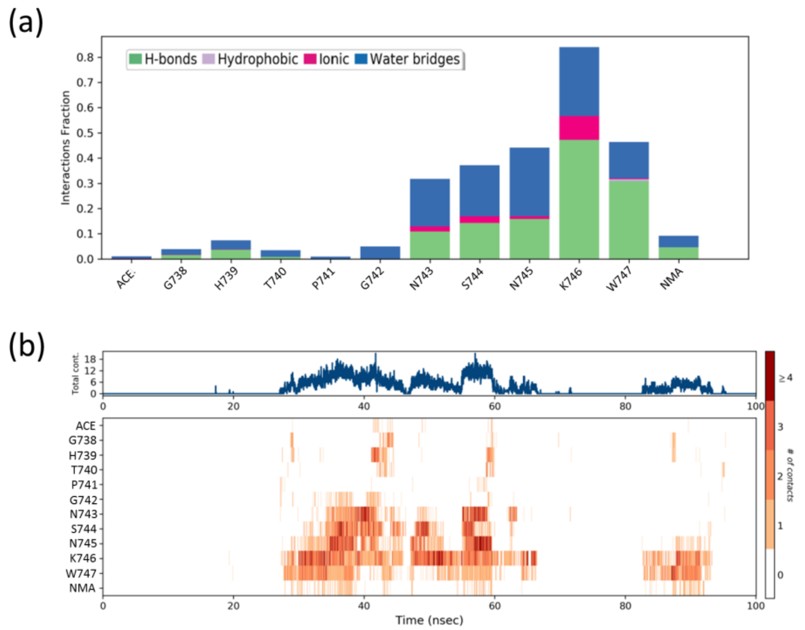

Figure S26. Results from the heparin/Pep2 simulation. (a) Interactions fraction per Pep2 residue. (c) Time-dependence of the total number of interactions and of interactions between each residue of peptide Pep2 and heparin.

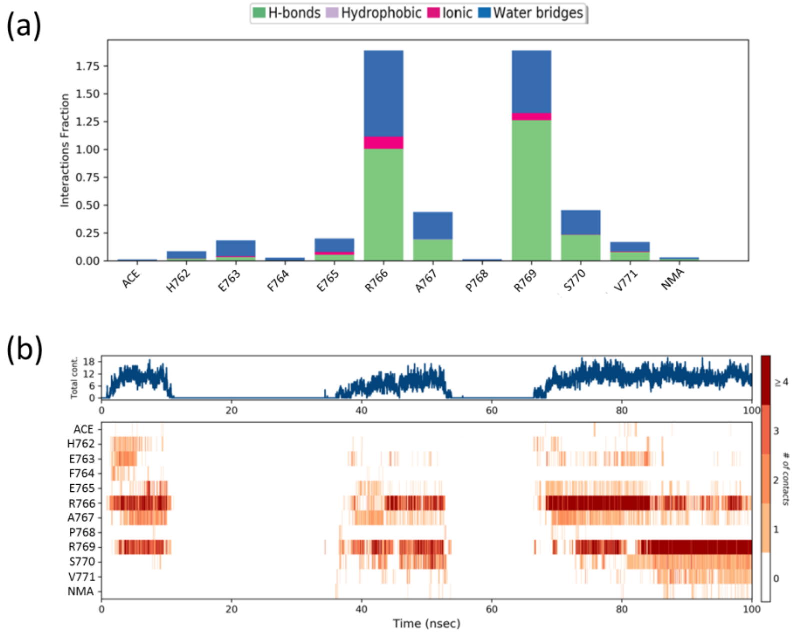

Figure S27. Results from the heparin/Pep3 simulation. (a) Interactions fraction per Pep3 residue. (c) Time-dependence of the total number of interactions and of interactions between each residue of peptide Pep3 and heparin.

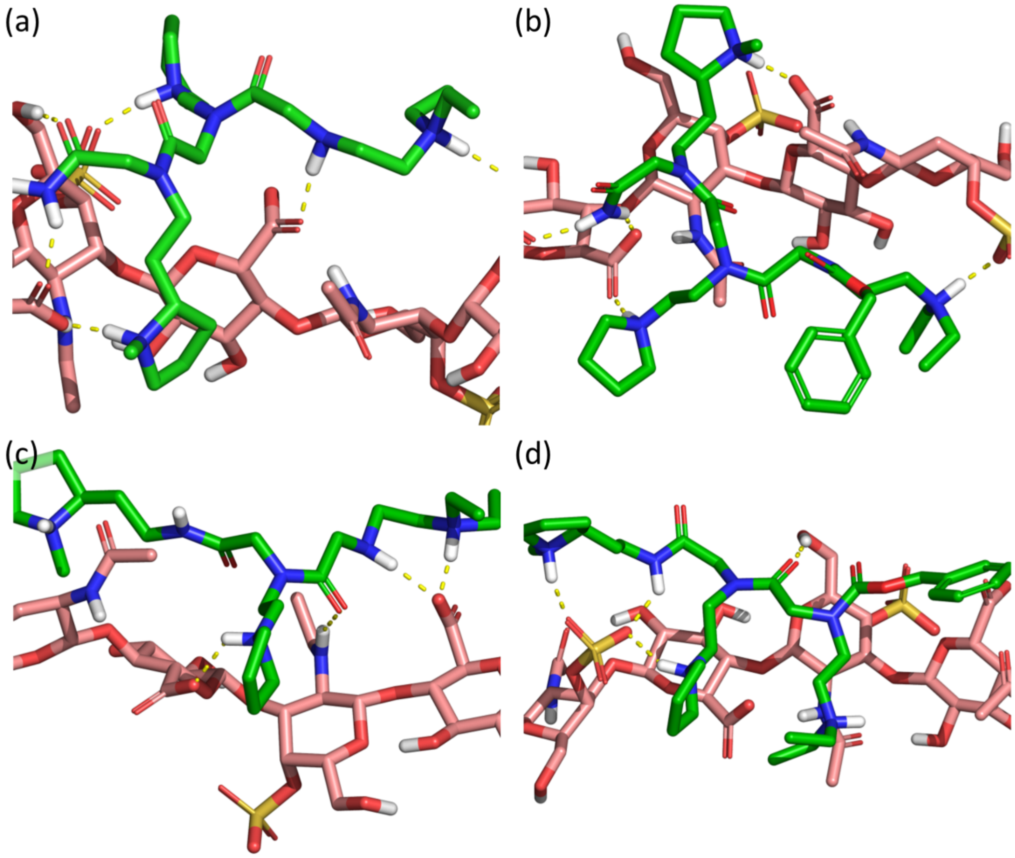

Figure S28. Best docked poses of (a) SICHI, (b) CSIC02, (c) CSIC03 and (d) CSIC04 to a dp8 CS-A model.

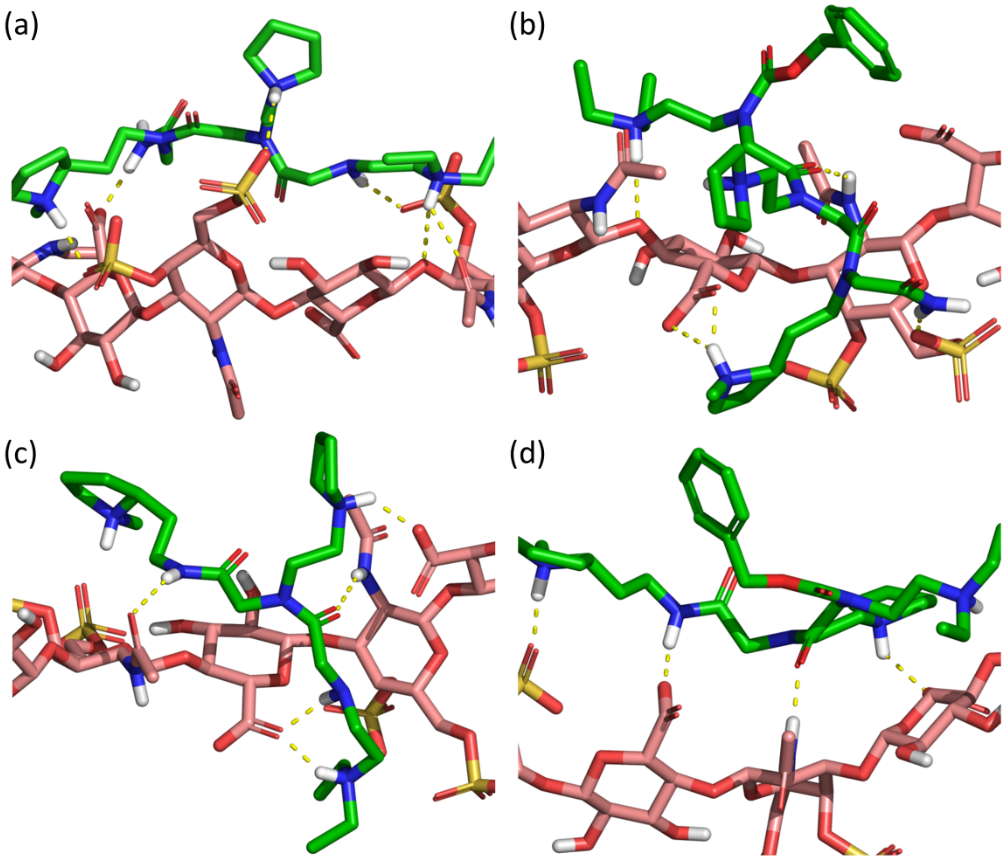

Figure S29. Best docked poses of (a) SICHI, (b) CSIC02, (c) CSIC03 and (d) CSIC04 to a dp8 CS-E model.

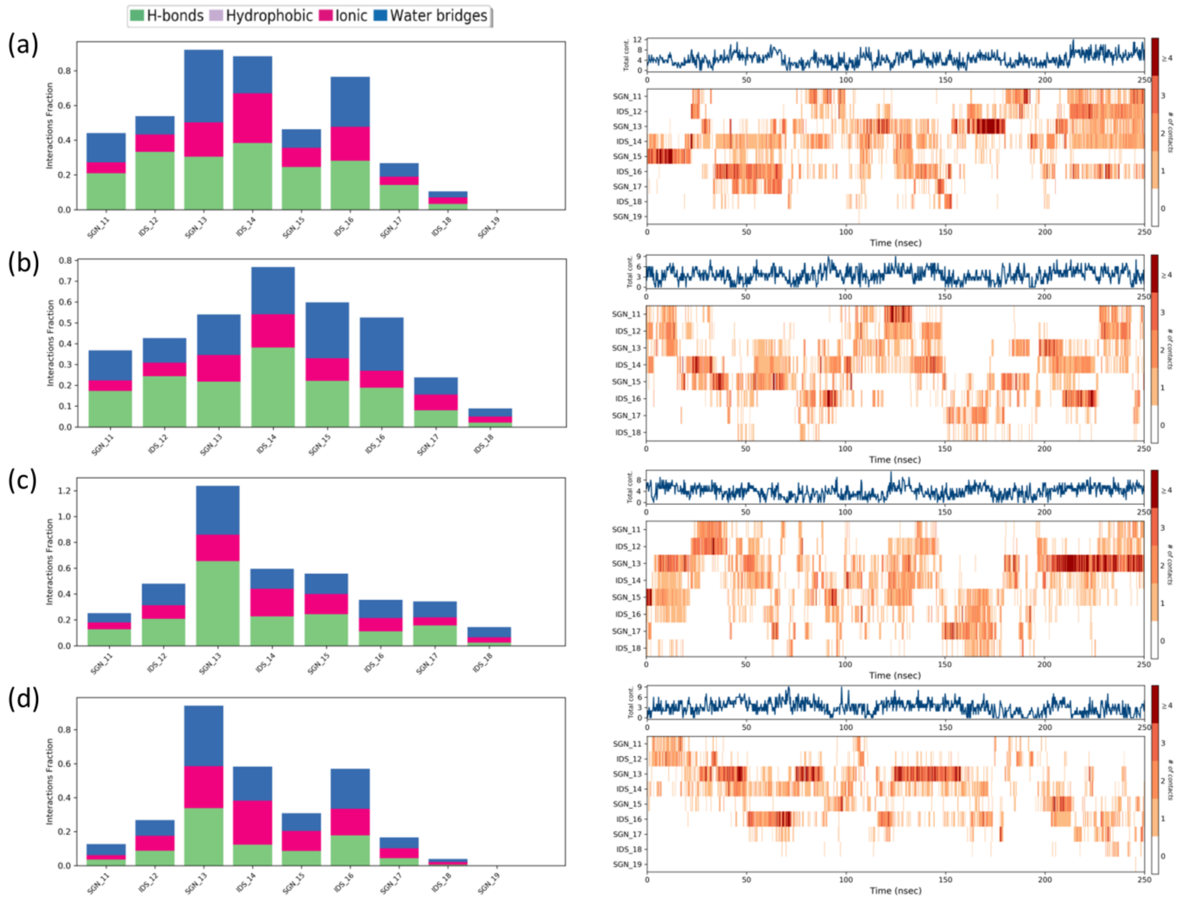

Figure S30. Results from the heparin/peptoids simulations for (a) SICHI, (b) CSIC02, (c) CSIC03 and (d) CSIC04. Left: Interactions fraction per heparin residue. Right: Time-dependence of the total number of interactions (blue) and of the interactions with each heparin residue (brown).

Synthesis and characterization of compounds

General: Reagents and solvents were purchased from commercial suppliers (Aldrich, Fluka, or Merck) and were used without further purification.

Flash chromatography: Flash chromatography purifications were performed on a BioTage instrument. Reversed-phase purifications using KP-C18-HS cartridges, and normal-phase purifications using KP-Sil cartridges.

NMR spectroscopy: The NMR spectroscopic experiments were carried out on a Varian MERCURY 400 spectrometer (400 MHz for ^{1}H and 100 MHz for ^{13}C) at 298 K. Chemical shifts are given in ppm (δ) relative to internal TMS, and coupling constants (*J*) are reported in Hertz (Hz).

Analytical RP-HPLC: Analytical RP-HPLC was performed with a Hewlett Packard Series 1100 (UV detector 1315A) modular system using a X-Terra C_{18} (15 x 0.46 cm, 5 μm). CH_3CN-H_2O Mixtures containing 0.1% TFA at 1 mL/min were used as mobile phase and monitoring wavelength was set at 220 nm. Gradient from 5% to 100 % of CH_3CN in 20 min.

Mass spectrometry: High resolution mass spectra (HRMS) were performed on Acquity UPLC System and a LCT PremierTM XE Benchtop orthogonal acceleration time-of-flight (oa-TOF) (Waters Corporation, Milford, MA) equipped with an electrospray ionization source.

Synthesis of the *N*-terminal monomer:

First step: Preparation of *tert*-butyl *N*-[2-(diethylamino)ethyl]glycinate

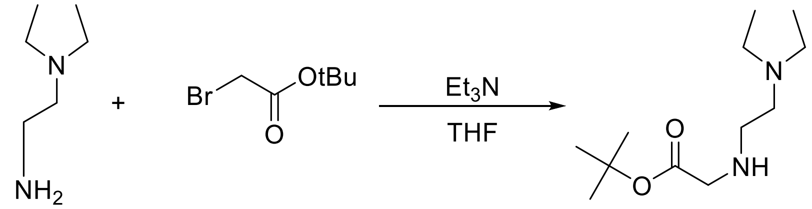

To a solution of *N*,*N*-diethylethylenediamine (3.6 mL, 25.8 mmol) and triethylamine (7.2 mL, 51.6 mmol) in 25 mL of THF, tert-butyl bromoacetate (3.8 mL,25.8 mmol) was added at 0 ºC. Then the mixture was allowed to react overnight at room temperature. The solvent was evaporated to dryness and the residue obtained was treated with water and extracted with CH_2Cl_2. The organic layer was washed with water, dried and filtered. The solvent was eliminated, the ^{1}H-NMR spectrum showed a ratio 4.6:1 monoalkylation and dialkylation products respectively. The residue was distilled under vacuum to yield 2.9 g of *tert*-butyl *N*-[2-(diethylamino)ethyl]glycinate (85 ºC, 0.3 Torrs, 49%). HRMS (M + 1): calcd. for $C_{12}H_{26}N_2O_2$: 231.2073. Found: 231.2075.

^{1}H NMR (400 MHz, $CDCl_3$, 298 K) δ 3.31 (s, 2H, $COCH_2NH$), 2.67 (m, 2H, CH_2NH), 2.55 (m, 6H, CH_2N), 1.46 (s, 9H, ^{t}Bu), 1.03 (t, *J* = 7.1 Hz, 6H, CH_3).

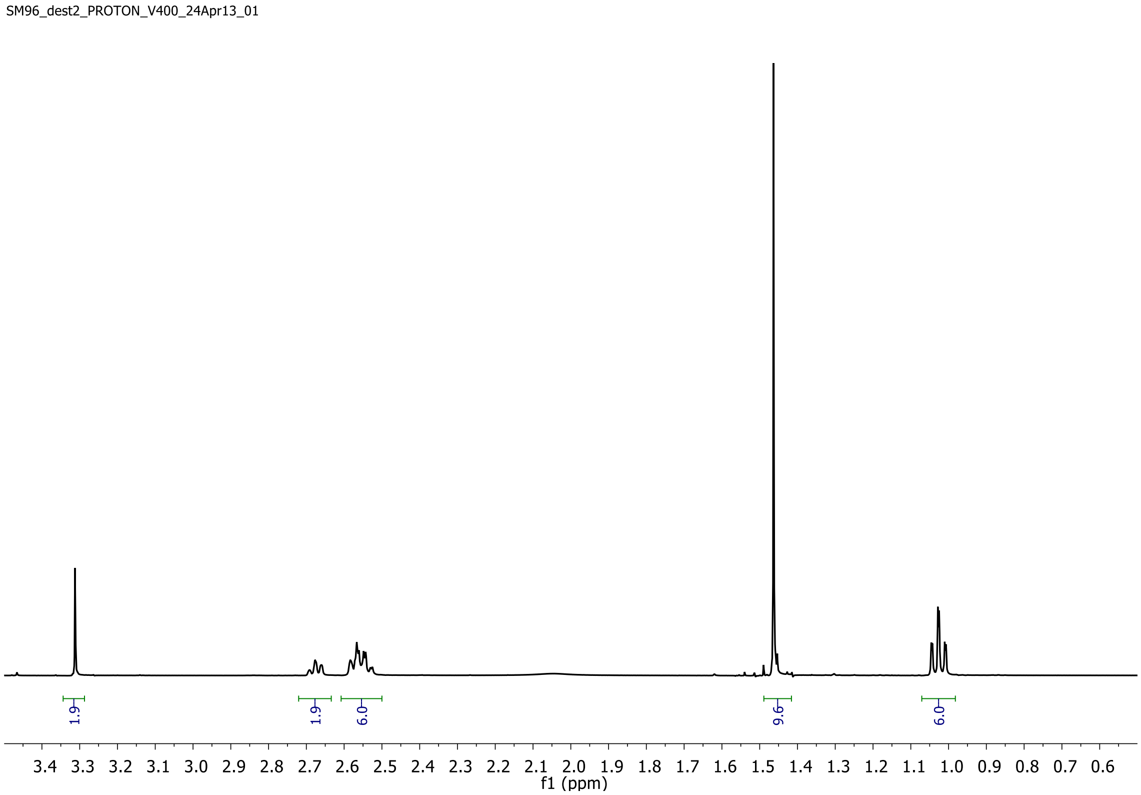

<u>Second step</u>: Preparation of *tert*-butyl *N*-benzyloxycarbonyl- *N*-[2-(diethylamino)ethyl]glycinate

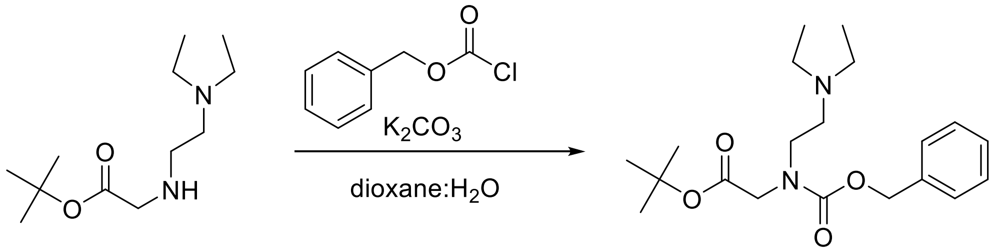

2.5 mL of benzyl chloroformate (17.7 mmol) were added at 0ºC to the solution of *tert*-butyl *N*-[2-(diethylamino)ethyl]glycinate (3.7 g, 16.1 mmol) and K_2CO_3 (6.7 g, 48.3 mmol) in 32 ml of dioxane: H_2O (1:1). The mixture was allowed to react overnight at room temperature. The organic solvent was evaporated, and the residue was extracted with CH_2Cl_2 (3 x 10 mL). The joined organic fractions were dried and filtered. The solvent was removed under reduced pressure and the residue was purified by flash chromatography using DCM: MeOH as eluent (from 0% to 2% MeOH, DCM contained 1% Et_3N) to give 5.1 g of *tert*-butyl *N*-benzyloxycarbonyl- *N*-[2-(diethylamino)ethyl]glycinate (87% yield, RT: 12.9 min). . HRMS (M + 1): calcd. for $C_{20}H_{32}N_2O_4$: 365.2440. Found: 365.2443.

^{1}H NMR (400 MHz, 2 rotamers, $CDCl_3$, 298 K) δ 7.36 – 7.28 (5H, CH_{Ar}), 5.15 (s, 2H, CH_2 - Cbz), 5.12 (s, 2H, CH_2 - Cbz), 4.00 (s, 2H, CH_2CO_2), 3.95 (s, 2H, CH_2CO_2), 3.43 (t, *J* = 7.0 Hz, 2H, NCH_2), 3.37 (m, 2H, NCH_2), 2.69 – 2.52 (m, 4H, NCH_2), 2.47 (q, *J* = 7.1 Hz, 2H, NCH_2), 1.46 (s, 9H, *tert*-butyl), 1.39 (s, 9H, *tert*-butyl), 1.03 (t, *J* = 6.6 Hz, 6H, CH_3), 0.95 (t, *J* = 7.1 Hz, 6H, CH_3).

^{13}C NMR (101 MHz, 2 rotamers, $CDCl_3$, 298 K) δ 169.2 (CO_2), 156.5 (CO_2N), 156.1 (CO_2N), 136.7 (C_{Ar}), 136.7 (C_{Ar}), 128.7 (CH_{Ar}), 128.7 (CH_{Ar}), 128.6 (CH_{Ar}), 128.5 (CH_{Ar}), 128.5 (CH_{Ar}), 128.1 (CH_{Ar}), 128.0 (CH_{Ar}), 127.9

(CH_{Ar}), 81.8 (C), 67.6 (CH_2 - Cbz), 67.3 (CH_2 - Cbz), 51.6 (CH_2), 51.3 (CH_2), 50.8 (CH_2), 50.7 (CH_2), 47.6 ($\underline{C}H_2CH_3$ x 2), 47.5 ($\underline{C}H_2CH_3$ x 2), 47.3 (CH_2), 46.8 (CH_2), 28.2 (CH_3 x 3), 28.1 (CH_3 x 3), 12.0 ($CH_2\underline{C}H_3$ x 2).

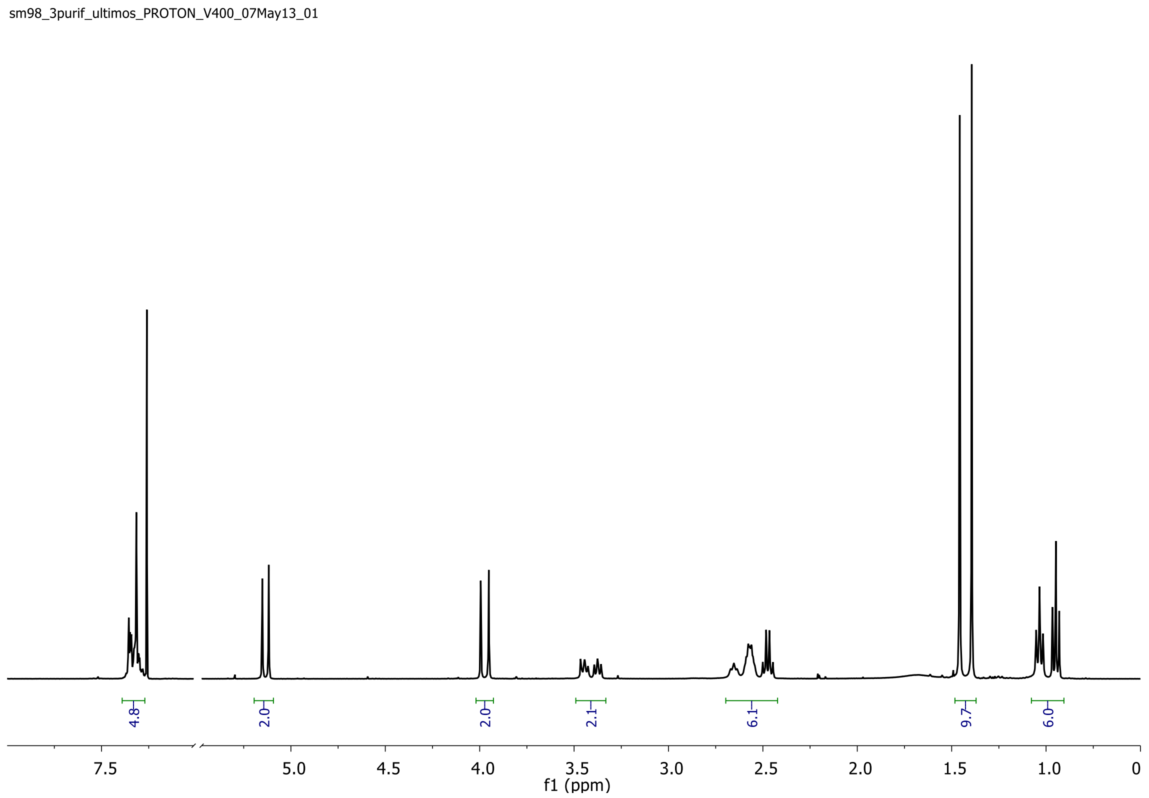

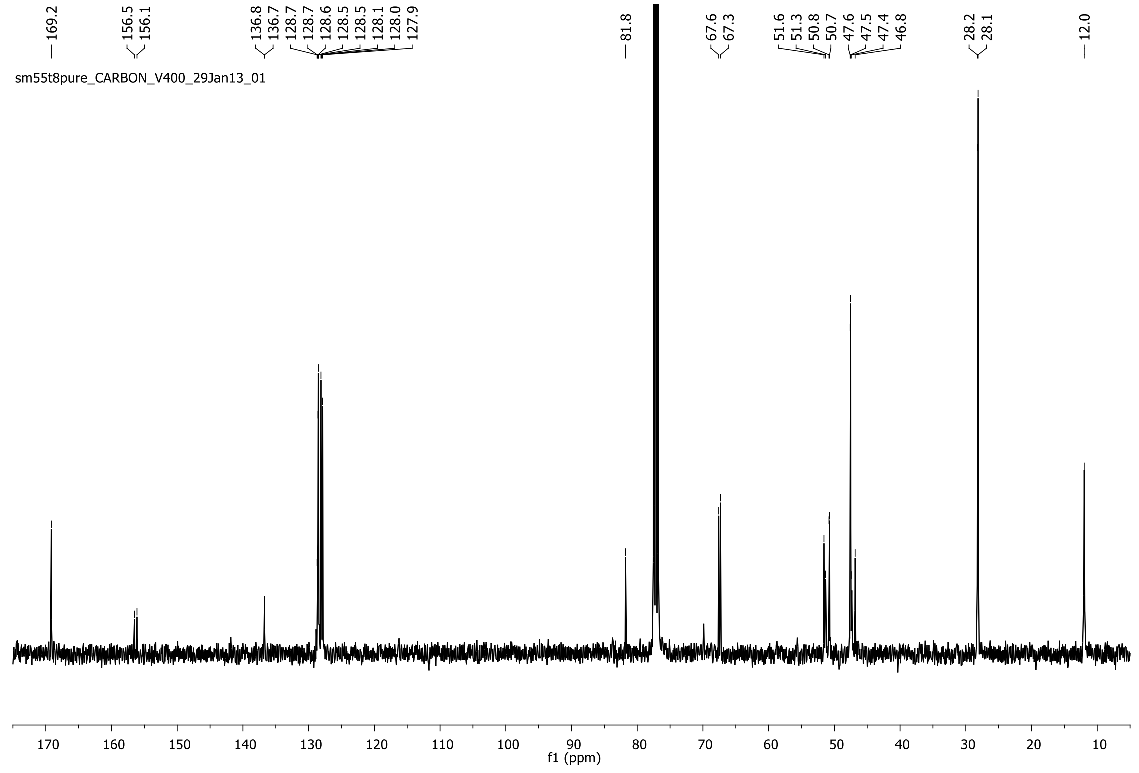

<u>Third step</u>: Preparation of *N*-benzyloxycarbonyl- *N*-[2-(diethylamino)ethyl]glycine hydrochloride

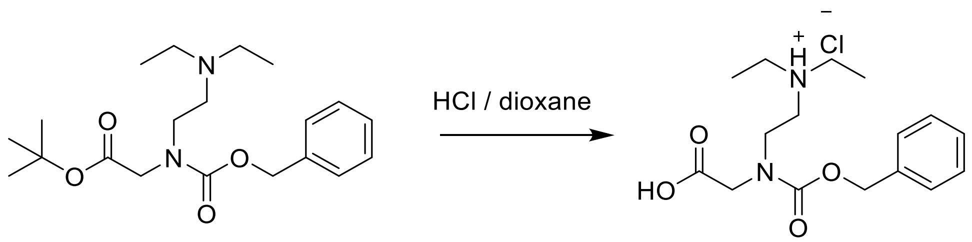

To 1.8 g of *tert*-butyl *N*-benzyloxycarbonyl- *N*-[2-(diethylamino)ethyl]glycinate (4.9 mmol) was added 6.0 mL of HCl/dioxane (4 M, 24.0 mmol), the mixture was allowed to react for 1 h at 60 ºC (HPLC control, RT: 10.6 min). Then, the solvent was removed under reduced pressure. 1.6 g of *N*-benzyloxycarbonyl- *N*-[2-(diethylamino)ethyl]glycine hydrochloride (99 % yield) was obtained and it was used without further purification. HRMS (M + 1): calcd. for $C_{16}H_{24}N_2O_4$: 309.1814. Found: 309.1822.

^{1}H NMR (400 MHz, 2 rotamers, D_2O, 298 K) δ 7.50 – 7.36 (5H, CH_{Ar}), 5.22 (s, 2H, CH_2 - Cbz), 5.17 (s, 2H, CH_2 - Cbz), 3.92 (s, 2H, CH_2CO_2), 3.89 (s, 2H, CH_2CO_2), 3.75 (m, 2H, NCH_2), 3.34 (t, *J* = 5.7 Hz, 2H, NCH_2), 3.27 (t, *J* = 5.9 Hz, 2H, NCH_2), 3.22 – 3.05 (m, 4H, NCH_2), 1.19 (t, *J* = 7.3 Hz, 6H, CH_3), 1.14 (t, *J* = 7.3 Hz, 6H, CH_3).

^{13}C NMR (101 MHz, 2 rotamers, D_2O, 298 K) δ 177.5 (CO), 177.2 (CO), 157.2 (NCO), 157.1 (NCO), 136.1 (C_{Ar}), 135.5 (C_{Ar}), 128.8 (CH_{Ar}), 128.8 (CH_{Ar}), 128.6 (CH_{Ar}), 128.2 (CH_{Ar}), 127.4 (CH_{Ar}), 68.3 (CH_2 – Cbz), 67.8 (CH_2 – Cbz), 52.4 (CH_2), 52.0 (CH_2), 50.1 (CH_2), 49.4 (CH_2), 47.0 (2 x CH_2), 46.9 (2 x CH_2), 44.3 (CH_2), 44.1 (CH_2), 7.9 (CH_3), 7.8 (CH_3).

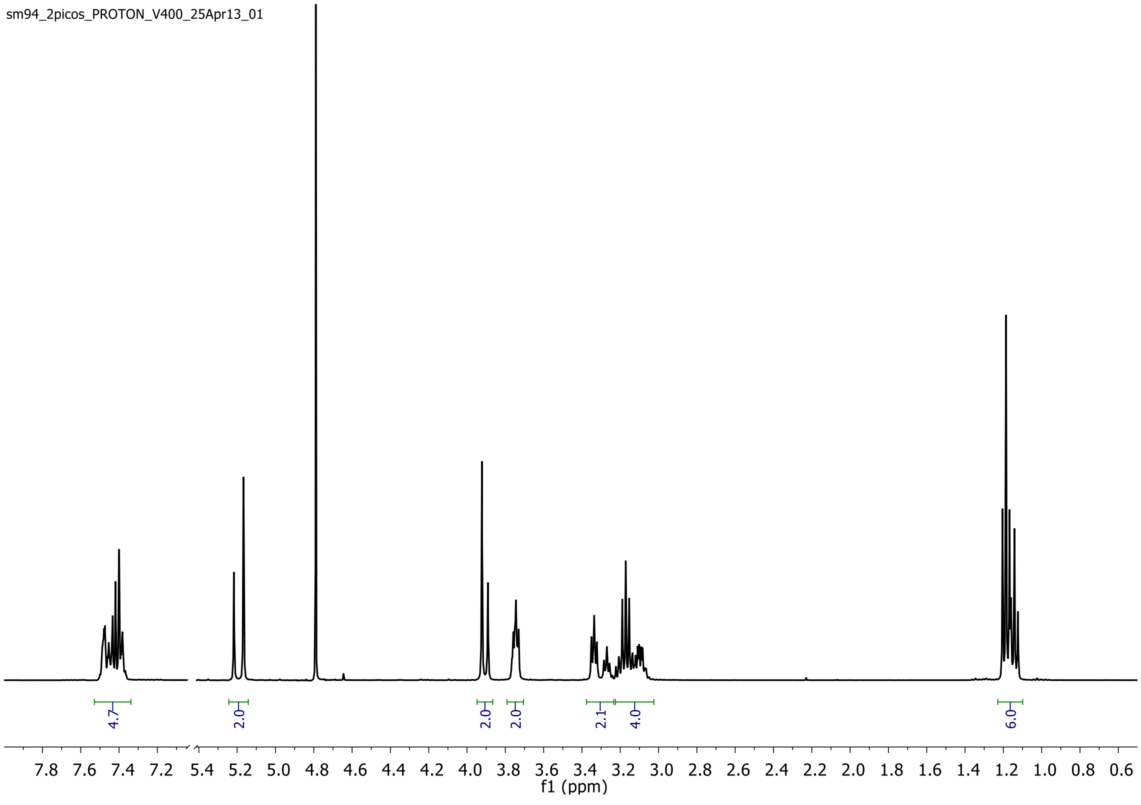

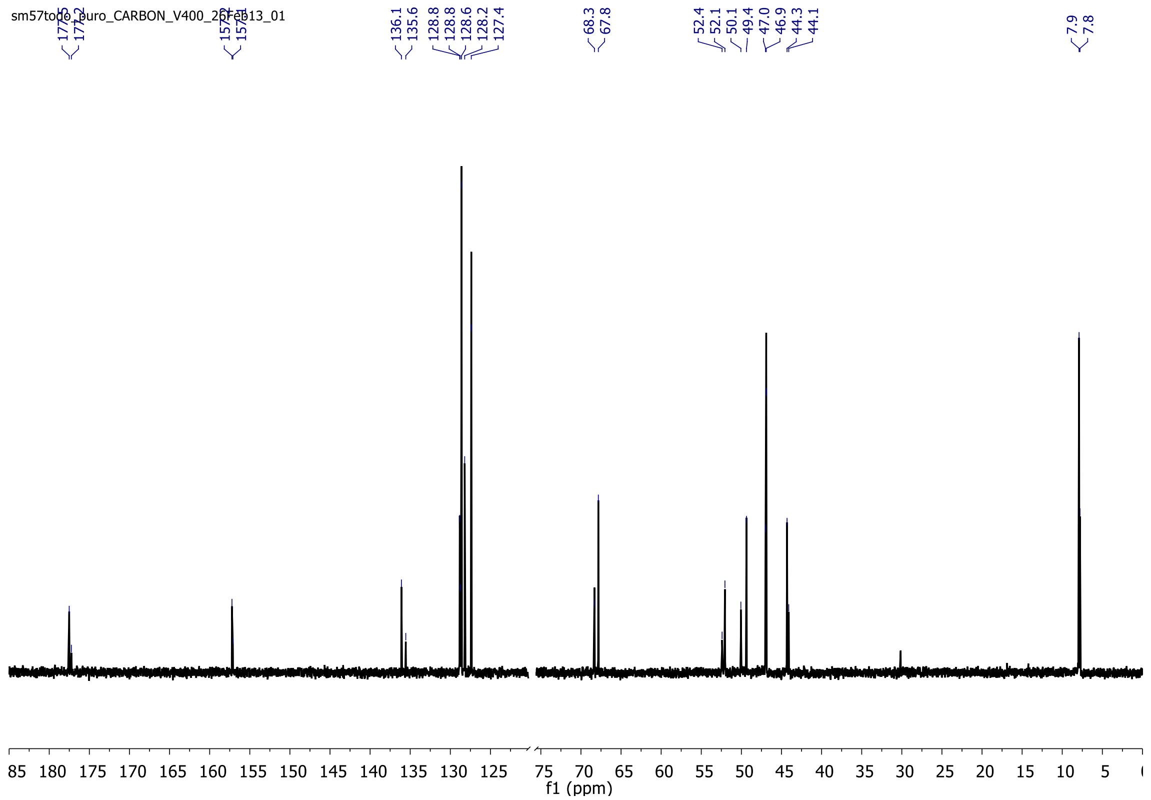

Synthesis of the central monomer:

Preparation of *tert-butyl N*-[(2-pyrrolidin-1-yl)ethyl]glycinate

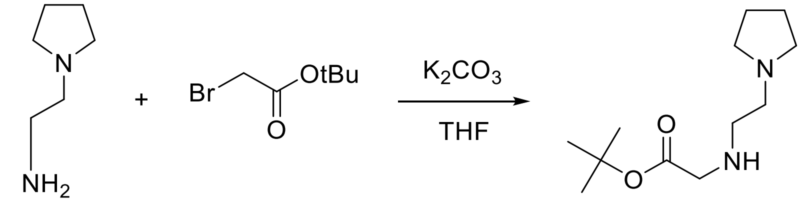

To a mixture of 1-(2-aminoethyl)pyrrolidine (3.0 mL, 23.7 mmol) and K_2CO_3 (9.8 g, 71 mmol) in 25 mL of THF, tert-butyl bromoacetate (3.5 mL, 23.7 mmol)was added at 0 ºC. The mixture was allowed to react for 30 min at room temperature, filtered and the solvent was evaporated to dryness. The residue obtained was treated with water (20 mL) and extracted with CH_2Cl_2 (25 mL), dried and filtered. The solvent was eliminated, the ^{1}H-NMR spectrum showed a ratio 5.6:1 monoalkylation and dialkylation products respectively. The residue was distilled under vacuum to yield 2.9 g of *tert-butyl N*-[(2-pyrrolidin-1-yl)ethyl]glycinate (85 ºC, 0.3 Torrs, 54%). HRMS (M + 1): calcd. for $C_{12}H_{24}N_2O_2$: 229.1916. Found: 229.1913.

^{1}H NMR (400 MHz, $CDCl_3$, 298 K) δ 3.30 (s, 2H, CH_2CO), 2.72 (dd, *J* = 7.0 and 6.2 Hz, 2H, CH_2), 2.58 (dd, *J* = 6.6 and 5.6 Hz, 2H, CH_2), 2.51 (t, *J* = 5.4 Hz, 4H, CH_2), 1.99 (s, 1H, NH), 1.76 (m, 4H, CH_2), 1.45 (s, 4H, CH_3).

^{13}C NMR (101 MHz, $CDCl_3$, 298 K) δ 171.9 (CO), 81.1 (C), 56.3 (CH_2), 54.4 (CH_2 x 2), 52.0 (CH_2), 48.2 (CH_2), 28.3 (CH_3), 23.6 (CH_2 x 2).

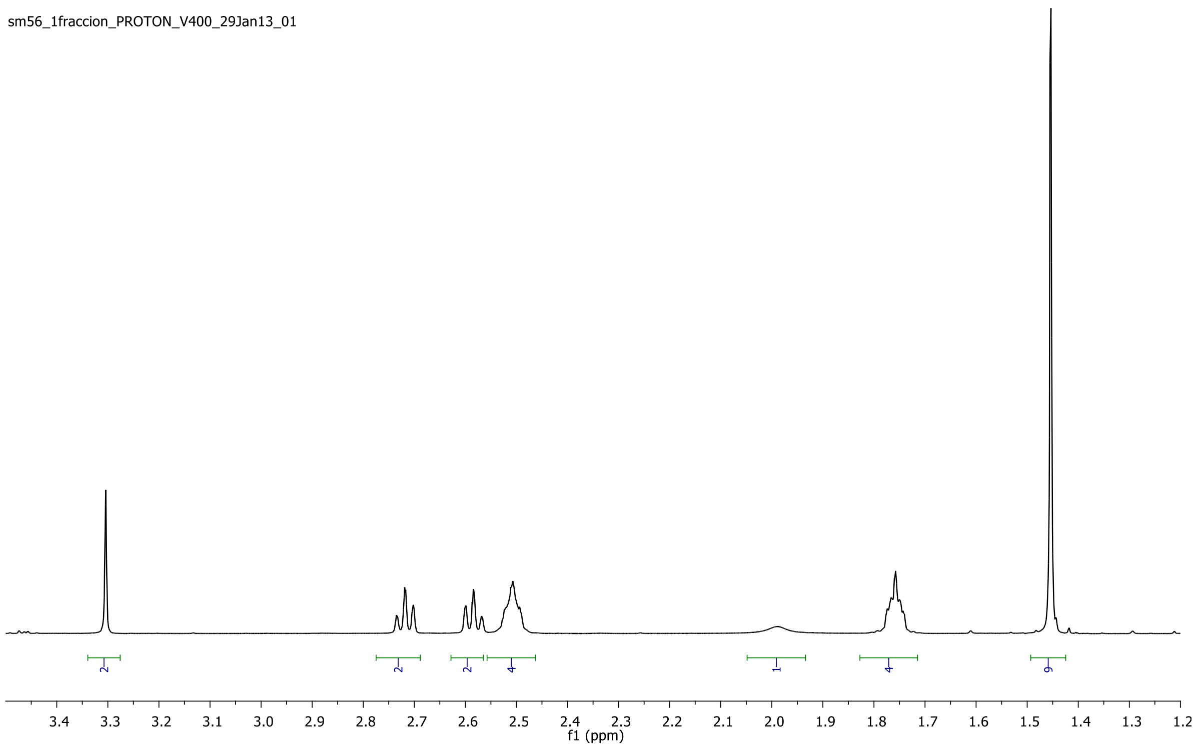
sm56_1fraccion_PROTON_V400_29Jan13_01
2
2
2
4
1
4
9
3.4 3.3 3.2 3.1 3.0 2.9 2.8 2.7 2.6 2.5 2.4 2.3 2.2 2.1 2.0 1.9 1.8 1.7 1.6 1.5 1.4 1.3 1.2
f1 (ppm)

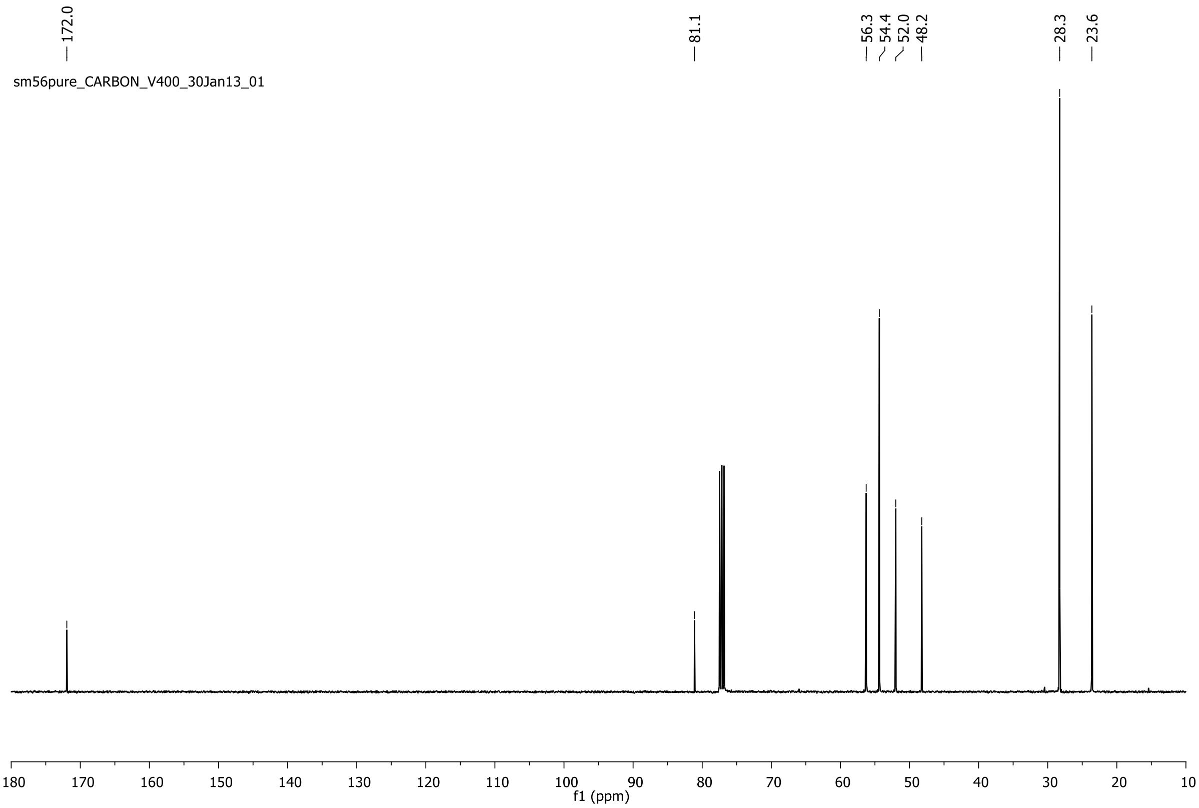
172.0
81.1
56.3
54.4
52.0
48.2
28.3
23.6
sm56pure_CARBON_V400_30Jan13_01
180 170 160 150 140 130 120 110 100 90 80 70 60 50 40 30 20 10
f1 (ppm)

Synthesis of the C-terminal monomer:

Preparation of [2-(1-methylpyrrolidin-2-yl)ethylamino]acetamide

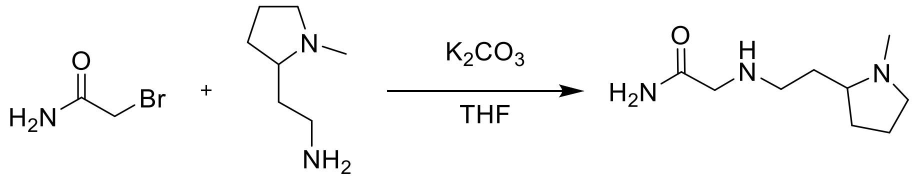

2 mL of 2-(2-Aminoethyl)-1-methylpyrrolidine (13.8 mmol) was dissolved in 75 mL of THF and 5.7 g of K_2CO_3 (41.4 mmol) were added. The mixture was cold at 0 ºC and bromoacetamide (1.9 g, 13.8 mmol) was added slowly. The new mixture was stirred for 2 h at room temperature, filtered and the THF was evaporated. The residue was distilled under vacuum to yield [2-(1-methylpyrrolidin-2-yl)ethylamino]acetamide (120 ºC, 0.3 Torrs). HRMS (M + 1): calcd. for $C_9H_{19}N_3O$: 186.1606. Found: 186.1592.

^{1}H NMR (400 MHz, $CDCl_3$, 298 K) δ 7.20 (s, 1H, NH_2), 5.52 (s, 1H, NH_2), 3.27 (dd, J = 16.8 and 3.9 Hz, 2H, CH_2CO), 3.06 (ddd, J = 9.7, 7.6, and 2.3 Hz, 1H, $\underline{CH_2}NCH_3$), 2.74 – 2.58 (m, 2H, CH_2NH), 2.32 (s, 3H, CH_3), 2.18 – 2.07 (m, 2H, 1H x $\underline{CH_2}NCH_3$ + 1H x CH), 1.98 – 1.64 (m, 4H, CH_2), 1.55 – 1.43 (m, 2H, CH_2), 1.42 (S, NH).

^{13}C NMR (101 MHz, $CDCl_3$, 298 K) δ 175.0 (CO), 64.7 (CH), 57.4 ($\underline{CH_2}NCH_3$), 52.8 (CH_2NH), 47.7 (CH_2CO), 40.7 (CH_3), 33.8 (CH_2), 30.7 (CH_2), 30.5 (CH_2), 22.2 (CH_2).

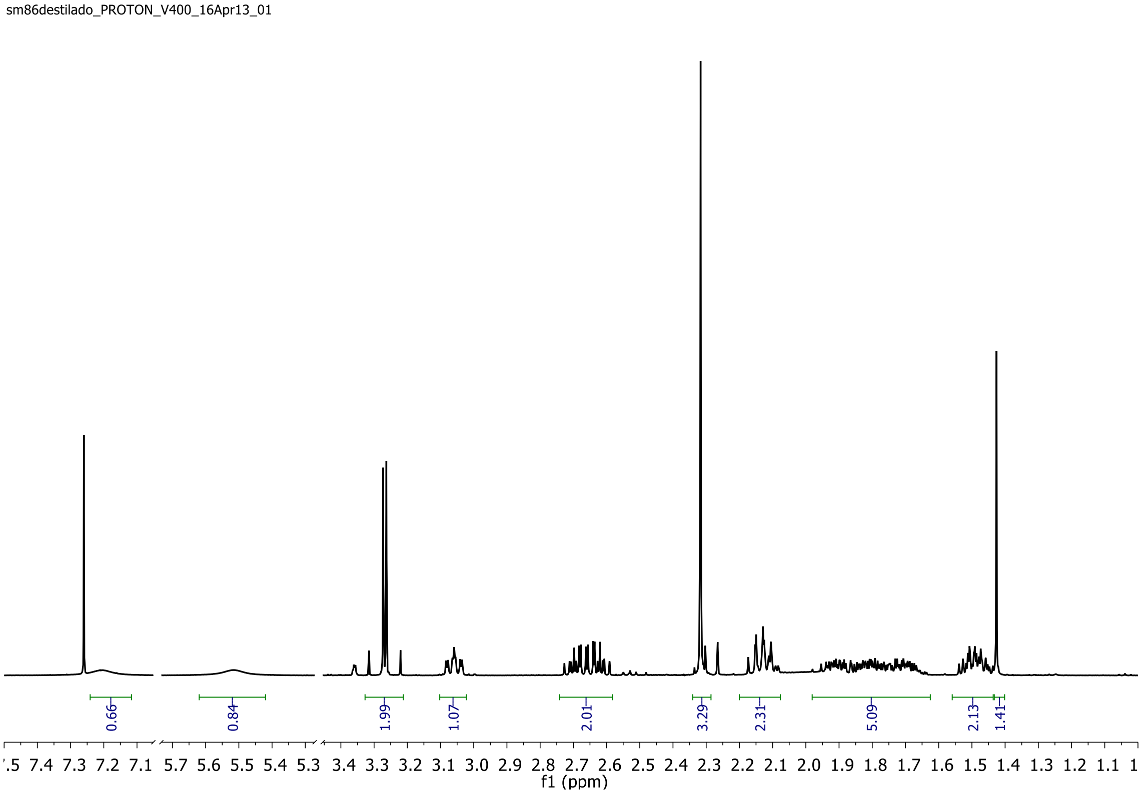

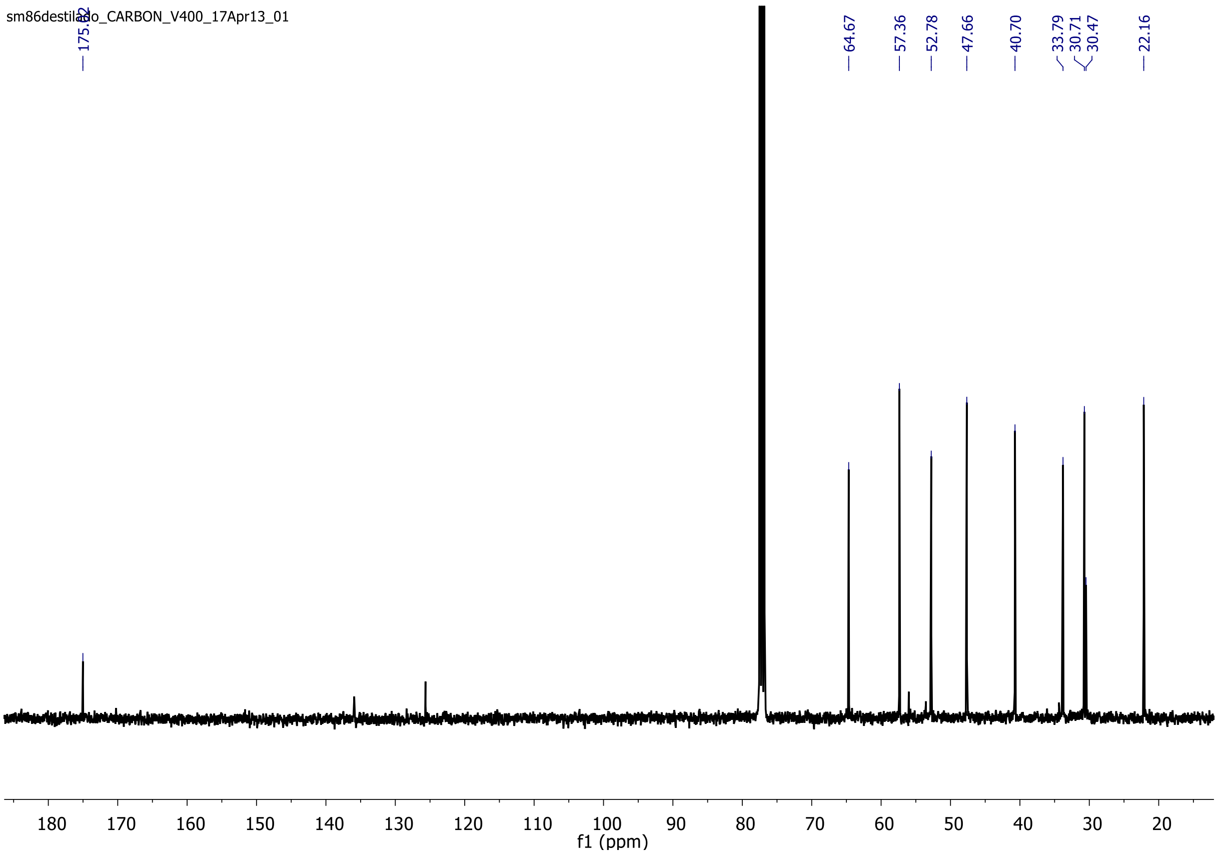

Synthesis of the dimer: Coupling of the N-terminal and central monomers

First step: Preparation of *tert*-butyl 3-(2-pyrrolidin-1-yl)ethyl-6-benzyloxycarbonyl-9-diethyl-4-oxo-3,6,9-triazanonanoate.

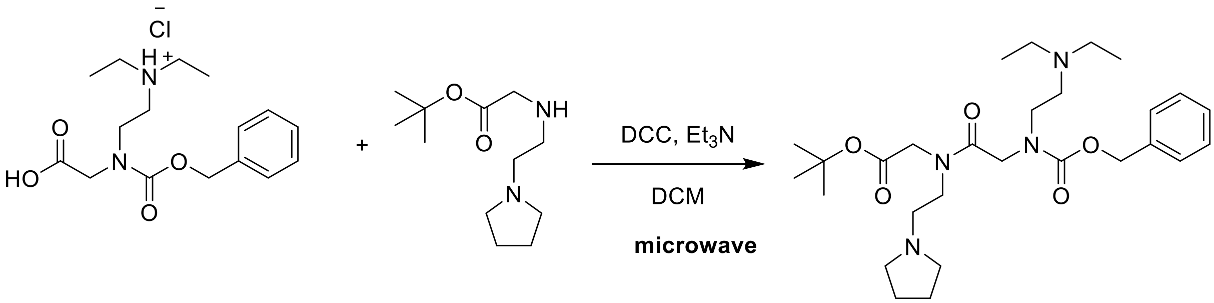

To a solution of *N*-benzyloxycarbonyl- *N*-[2-(diethylamino)ethyl]glycine hydrochloride (644 mg, 1.87 mmol) in 10.0 mL of anhydrous DCM, was added Et_3N (340 µL, 2.43 mmol) and DCC (503 mg, 2.43 mmol). The mixture was stirred 3 min at 60 ºC under microwave conditions and *tert-butyl N*-[(2-pyrrolidin-1-yl)ethyl]glycinate (470 mg, 2.06 mmol) was added. The new mixture was stirred under microwave conditions for 40 min at 60 ºC (HPLC control, RT: 10.7 min). The crude was filtered and the solid washed with DCM, the filtered was evaporated in vacuum. The residue was purified by reverse-phase chromatography using CH_3CN/H_2O as eluent (from 5% to 50% CH_3CN) to give 504.2 mg of *tert*-butyl 3-(2-pyrrolidin-1-yl)ethyl-6-benzyloxycarbonyl-9-diethyl-4-oxo-3,6,9-triazanonanoate (52% yield). HRMS (M + 1): calcd. for $C_{28}H_{46}N_4O_5$: 519.3546. Found: 519.3568.

^{1}H NMR (400 MHz, rotamers, $CDCl_3$, 298 K) δ 7.35 – 7.25 (5H, CH_{Ar}), 5.12 (s, 2H, CH_2 - Cbz), 5.09 (s, 2H, CH_2 - Cbz), 5.07 (s, 2H, CH_2 - Cbz), 4.27 (s, 2H, CH_2CO_2), 4.22 (s, 2H, CH_2CO_2), 4.06 (s, 2H, CH_2CO_2), 4.04 (s, 2H, CH_2CO_2), 4.03 (s, 2H, CH_2CO_2), 4.02 (s, 2H, CH_2CO_2), 3.97 (s, 2H, CH_2CO_2), 3.88 (s, 2H, CH_2CO_2), 3.59 – 3.20 (m, 4H, NCH_2), 2.76 – 2.38 (m, 12H, CH_2), 1.85 – 1.66 (m, 4H, CH_2 - pyrrolidine), 1.47 (s, 9H, *tert*-butyl), 1.45 (s, 9H, *tert*-butyl), 1.43 (s, 9H, *tert*-butyl), 1.03 (t, *J* = 6.6 Hz, 6H, CH_3), 1.05 (td, *J* = 7.1 and 3.4 Hz, 6H, CH_3), 0.95 (td, *J* = 7.1 and 4.8 Hz, 6H, CH_3).

^{13}C NMR (101 MHz, rotamers, $CDCl_3$, 298 K) δ 169.8 (CO), 169.7 (CO), 169.1 (CO), 169.0 (CO), 168.7 (CO), 168.4 (CO), 168.3 (CO), 168.2 (CO), 156.5 (NCO_2), 156.47, 156.4 (NCO_2), 156.3 (NCO_2), 136.6 (C_{Ar}), 136.6 (C_{Ar}), 136.5 (C_{Ar}), 128.6 (CH_{Ar}), 128.5 (CH_{Ar}), 128.2 (CH_{Ar}), 128.1 (CH_{Ar}), 128.0 (CH_{Ar}), 127.9 (CH_{Ar}), 83.0 (C), 81.9 (C), 67.8 (CH_2 - Cbz), 67.7 (CH_2 – Cbz), 67.5 (CH_2 – Cbz), 54.3 (CH_2 - pyrrolidine), 54.2 (CH_2 - pyrrolidine), 54.1 (CH_2 - pyrrolidine), 53.9 (CH_2 - pyrrolidine), 52.9 (CH_2 - pyrrolidine), 50.9 (CH_2CO), 50.6 (CH_2CO), 50.6 ($\underline{C}H_2CH_3$), 49.9 ($\underline{C}H_2CH_3$), 49.7 (CH_2CO), 49.6 (CH_2CO), 49.4 (CH_2CO), 49.3 (CH_2CO), 47.4 (CH_2), 47.2 (CH_2), 47.2 (CH_2), 46.9 (CH_2), 46.8 (CH_2), 46.3 (CH_2), 46.2 (CH_2), 45.9 (CH_2), 45.8 (CH_2), 45.7 (CH_2), 28.2 (CH_3), 28.2 (CH_3), 28.1 (CH_3), 23.6 (CH_2 - pyrrolidine), 23.6 (CH_2 - pyrrolidine), 11.1 ($CH_2\underline{C}H_3$), 10.8 ($CH_2\underline{C}H_3$), 10.4 ($CH_2\underline{C}H_3$), 10.1 ($CH_2\underline{C}H_3$).

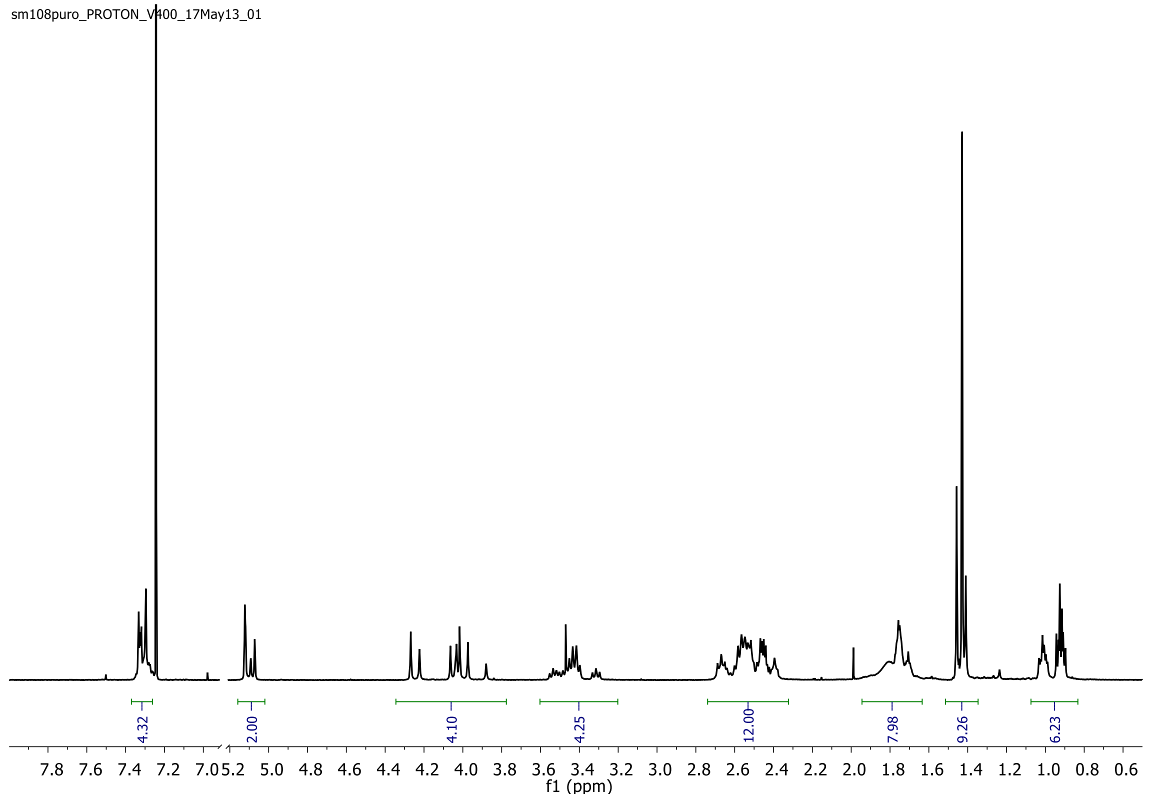

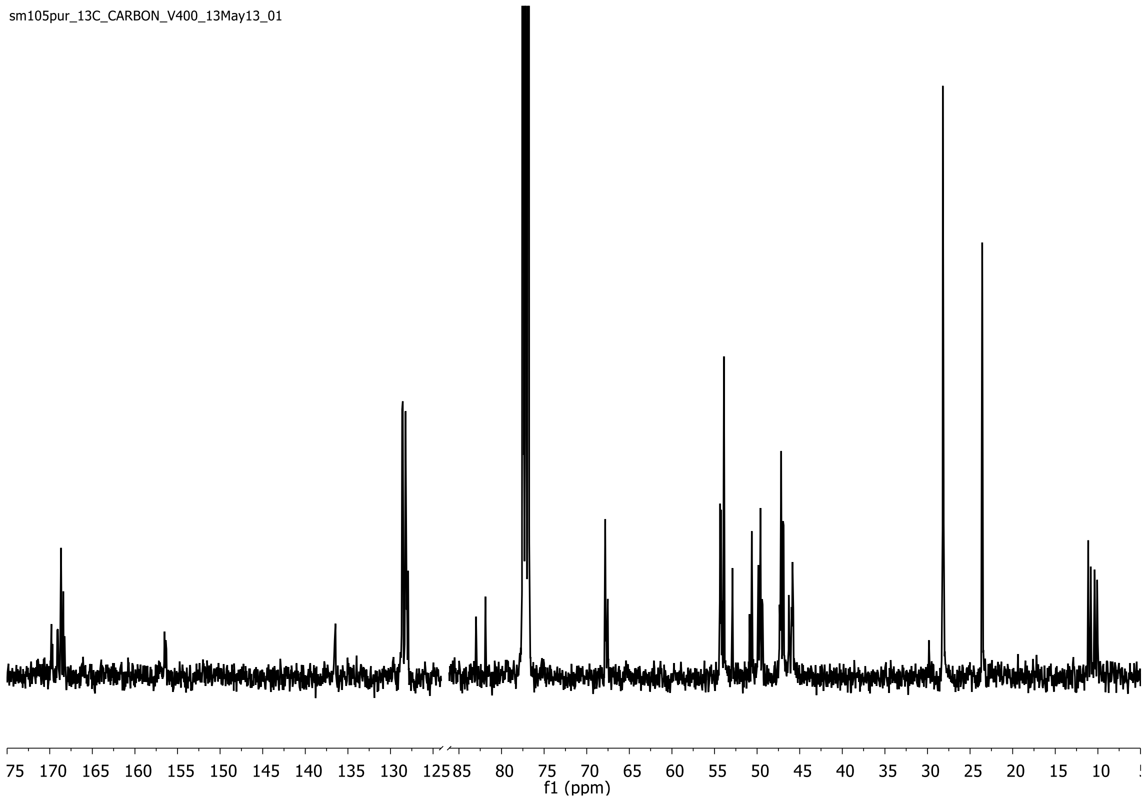

Second step:

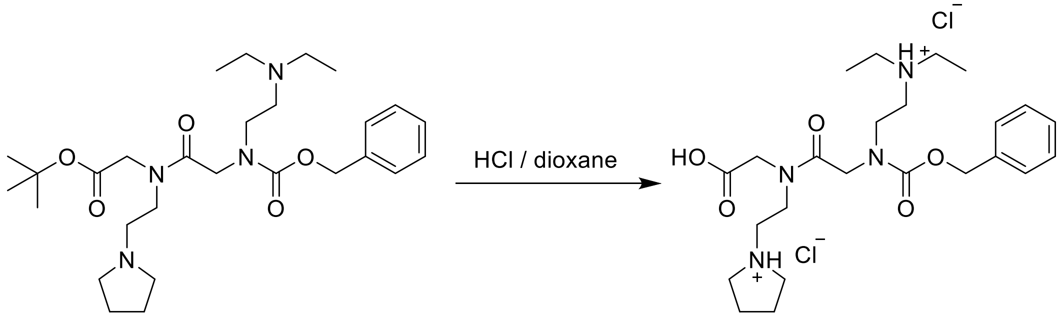

To 500 mg of *tert*-butyl 3-(2-pyrrolidin-1-yl)ethyl-6-benzyloxycarbonyl-9-diethyl-4-oxo-3,6,9-triazanonanoate (0.96 mmol) was added 2.2 mL of HCl/dioxane (4 M, 8.69 mmol), the mixture was allowed to react for 1 h at 60 ºC (HPLC control, RT: 9.0 min). Then, the solvent was removed under reduced pressure. 515.4 g of 3-(2-pyrrolidin-1-yl)ethyl-6-benzylloxycarbonyl-9-diethyl-4-oxo-3,6,9-triazanonanoic acid dihydrochloride (99 % yield) was obtained and it was used without further purification. HRMS (M + 1): calcd. for $C_{24}H_{38}N_4O_5$: 463.2920. Found: 463.2908.

^{1}H NMR (400 MHz, rotamers, D_2O, 298 K) δ 7.56 – 7.37 (5H, CH_{Ar}), 5.21 (s, 2H, CH_2 - Cbz), 5.15 (s, 2H, CH_2 - Cbz), 5.10 (s, 2H, CH_2 - Cbz), 4.40 (s, 2H, NCH_2CO), 4.38 (s, 2H, NCH_2CO), 4.33 (s, 2H, NCH_2CO), 4.25 (s, 2H, NCH_2CO), 4.24 (s, 2H, NCH_2CO), 4.21 (s, 2H, NCH_2CO), 4.20 (s, 2H, NCH_2CO), 4.09 (s, 2H, NCH_2CO), 3.91 – 2.88 (m, 16H, NCH_2), 2.16 – 1.91 (m, 4H, CH_2 - pyrrolidine), 1.27 (m, 6H, CH_3), 1.15 (t, *J* = 7.1 Hz, 6H, CH_3).

^{13}C NMR (101 MHz, rotamers, D_2O, 298 K) δ 172.7 (CO), 172.6 (CO), 172.5 (CO), 172.4 (CO), 172.3 (CO), 172.0 (CO), 166.2 (CO), 157.4 (NCO_2), 157.3 (NCO_2), 157.0 (NCO_2), 135.6 (C_{Ar}), 135.3 (C_{Ar}), 128.9 (CH_{Ar}), 128.8 (CH_{Ar}), 128.8 (CH_{Ar}), 128.7 (CH_{Ar}), 128.6 (CH_{Ar}), 127.8 (CH_{Ar}), 68.8 (CH_2 - Cbz), 68.7 (CH_2 - Cbz), 68.2 (CH_2 – Cbz), 54.8 (CH_2 - pyrrolidine), 54.6 (CH_2 - pyrrolidine), 54.6 (CH_2 - pyrrolidine), 54.5 (CH_2 - pyrrolidine), 52.2 (CH_2), 51.7 (CH_2), 51.6 (CH_2), 51.0 (CH_2), 50.9 (CH_2), 49.7 (NCH_2CO), 49.7 (NCH_2CO), 49.6 (CH_2), 49.5 (NCH_2CO), 49.3 (NCH_2CO), 49.3 (CH_2), 49.3 (NCH_2CO), 49.2 (NCH_2CO), 48.0 (CH_2), 47.6 (CH_2), 47.5 ($\underline{C}H_2CH_3$), 47.5 (CH_2), 47.4 ($\underline{C}H_2CH_3$), 47.3 (CH_2), 44.4 (CH_2), 44.2 (CH_2), 44.1 (CH_2), 43.9 (CH_2), 43.3 (CH_2), 42.2 (CH_2), 41.0 (CH_2), 22.7 (CH_2 - pyrrolidine), 22.6 (CH_2 - pyrrolidine), 22.5 (CH_2 - pyrrolidine), 22.5 (CH_2 - pyrrolidine), 8.0 (CH_3), 8.0 (CH_3), 7.9 (CH_3), 7.8 (CH_3), 7.8 (CH_3).

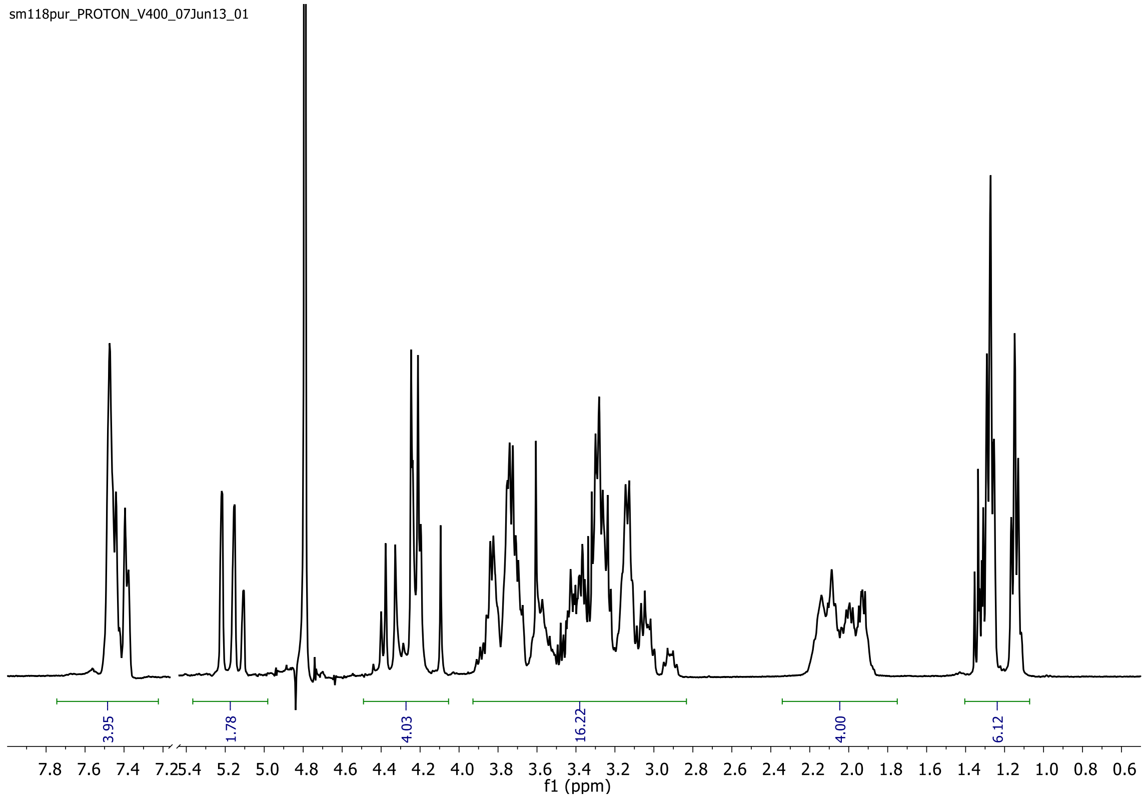

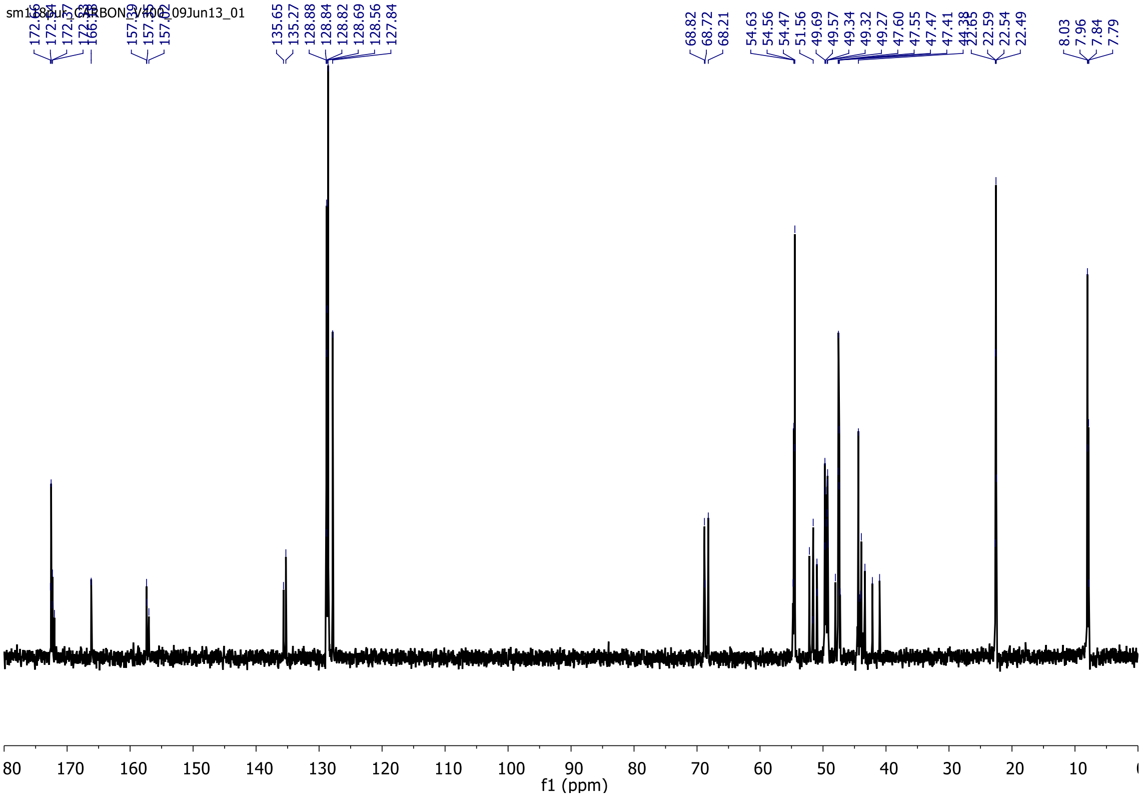
sm118pur_CARBON_V400_09Jun13_01
135.65
135.27
128.88
128.84
128.82
128.69
128.56
127.84
68.82
68.72
68.21
54.63
54.56
54.47
51.56
49.69
49.57
49.34
49.32
49.27
47.60
47.55
47.47
47.41
44.38
22.59
22.54
22.49
8.03
7.96
7.84
7.79
f1 (ppm)

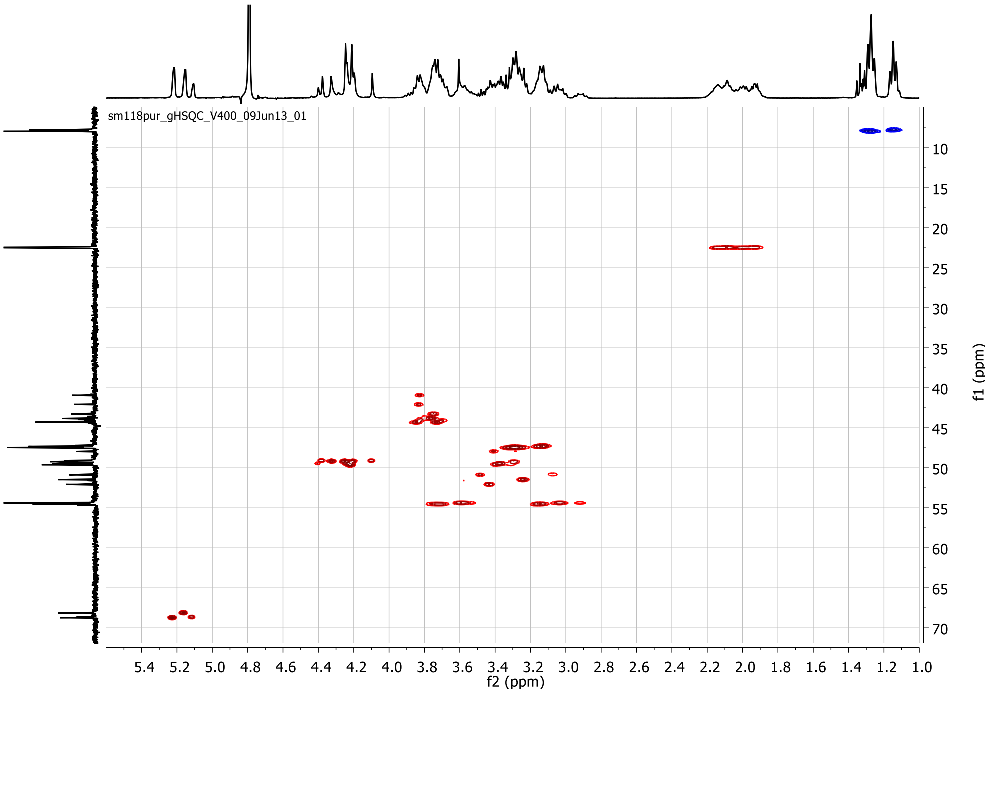
sm118pur_gHSQC_V400_09Jun13_01
f2 (ppm)
f1 (ppm)

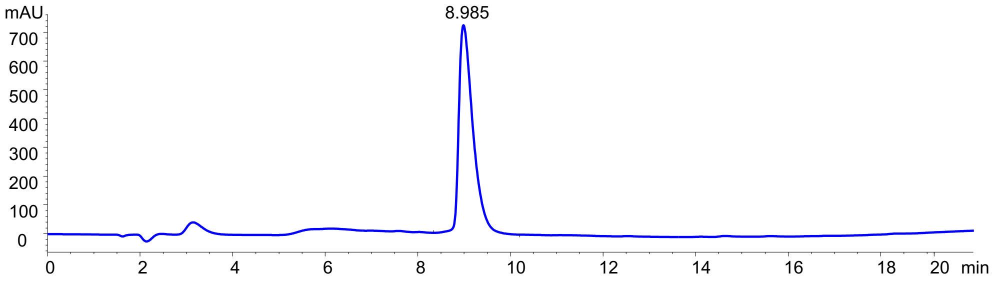

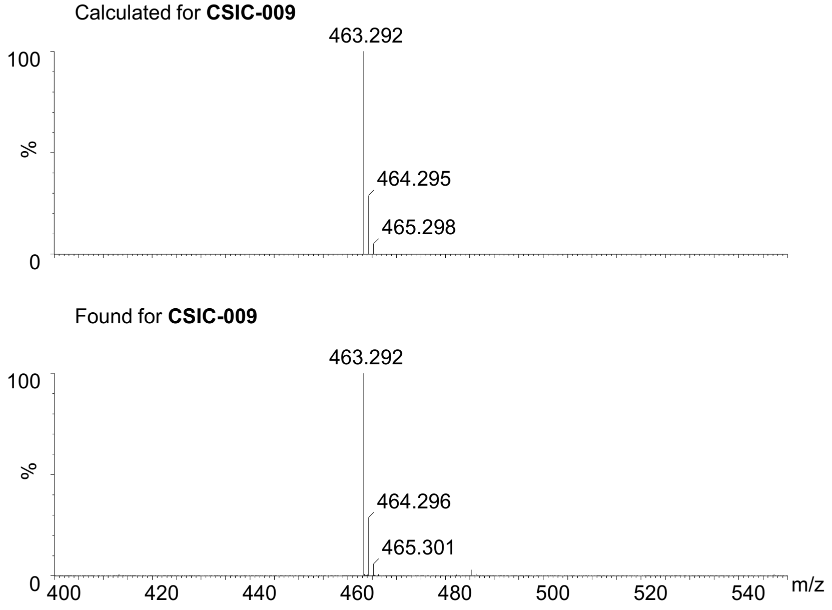

Synthesis of the trimer: Coupling of the C-terminal monomer and the dimer

Preparation of CSIC-02

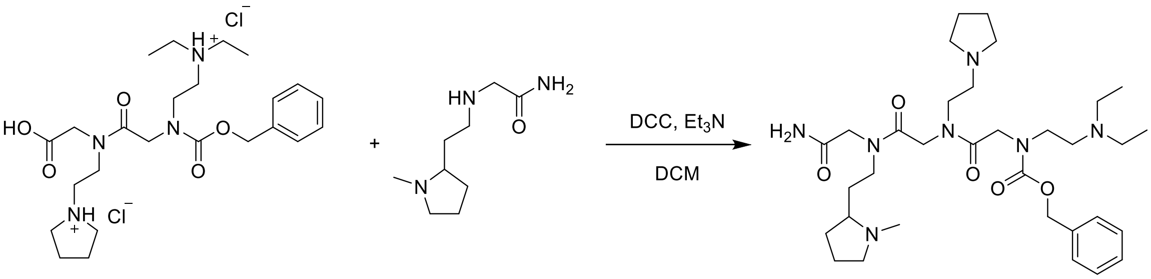

CSIC-002

To a solution of 3-(2-pyrrolidin-1-yl)ethyl-6-benzylloxycarbonyl-9-diethyl-4-oxo-3,6,9-triazanonanoic acid dihydrochloride (290 mg, 0.54 mmol) in 4.0 mL of anhydrous DCM, was added Et_3N (230 µL, 1.63 mmol) and DCC (145.7 mg, 0.71 mmol). The mixture was stirred 2 min at 45 ºC under microwave conditions and the [2-(1-methylpyrrolidin-2-yl)ethylamino]acetamide was added. The new mixture was stirred under microwave conditions for 1.5 h at 45 ºC (HPLC control, RT: 8.1 min). The crude was filtered and the solid washed with DCM,

then the filtered was evaporated in vacuum. The residue was purified by reverse-phase chromatography using CH_3CN/H_2O as eluent (from 0% to 40% CH_3CN) to give 78.4 mg of 3-[2-(1-methylpyrrolidin-2-yl)ethyl]-6-(2-pyrrolidin-1-yl)ethyl-9-benzyloxycarbonyl-12-diethyl-4,7-dioxo-3,6,9,12-tetraazadodecancarboxamide (23% yield). HRMS (M + 1): calcd. for $C_{33}H_{55}N_7O_5$: 630.4343. Found: 630.4339.

^{1}H NMR (400 MHz, rotamers, CD_3CN, 298 K) δ 7.40-7.26 (5H, CH_{Ar}), 6.58 (NH), 6.43 (NH), 6.37 (NH), 6.29 (NH), 6.25 (NH), 5.93 (NH), 5.70 (NH), 5.10 (s, 2H, CH_2 – Cbz), 5.09 (s, 2H, CH_2 – Cbz), 5.05 (s, 2H, CH_2 – Cbz), 5.04 (s, 2H, CH_2 – Cbz), 5.04 (s, 2H, CH_2 – Cbz), 4.35 – 3.83 (6H, $COCH_2$), 3.44 – 3.22 (m, 6H, NCH_2), 2.95 (m, 1H, $\underline{CH_2}NCH_3$), 2.67 – 2.41 (m, 12H, NCH_2), 2.24 – 2.21 (3H, NCH_3), 2.08 (m, 2H, 1H x CH + 1H x $\underline{CH_2}NCH_3$), 1.93 – 1.52 (m, 8H, 4H x CH_2 – pyrrolidine + 2H x $\underline{CH_2}CH_2NCH_3$ + 2H x $\underline{CH_2}CH$), 1.50 (m, 2H, $\underline{CH_2}CH$), 0.99 – 0.89 (m, 6H, CH_3).

^{13}C NMR (101 MHz, rotamers, CD_3CN, 298 K) δ 171.9 (CO), 170.2 (CO), 169.7 (CO), 169.4 (CO), 157.3 (NCO), 157.1 (NCO), 137.7 (C_{Ar}), 137.6 (C_{Ar}), 129.41 (CH_{Ar}), 129.3 (CH_{Ar}), 128.9 (CH_{Ar}), 128.8 (CH_{Ar}), 128.8 (CH_{Ar}), 128.7 (CH_{Ar}), 128.6 (CH_{Ar}), 128.5 (CH_{Ar}), 128.3 (CH_{Ar}), 128.3 (CH_{Ar}), 67.7 (CH_2 - Cbz), 67.5 (CH_2 - Cbz), 67.3 (CH_2 - Cbz), 64.8 (CH), 64.8 (CH), 64.3 (CH), 57.8 ($\underline{C}H_2NCH_3$), 55.4 (NCH_2) , 55.2 (NCH_2), 55.1 (NCH_2), 55.0 (NCH_2), 54.9 (NCH_2), 54.8 (NCH_2), 54.7 (NCH_2), 52.2 (NCH_2), 51.9 (NCH_2), 51.6 (NCH_2), 50.9 ($COCH_2$), 50.4 ($COCH_2$), 50.3 ($COCH_2$), 50.2 ($COCH_2$), 50.1 ($COCH_2$), 49.9 ($COCH_2$), 49.8 ($COCH_2$), 49.7 ($COCH_2$), 48.5 ($COCH_2$), 48.5 ($COCH_2$), 48.3 (NCH_2), 48.1 ($\underline{C}H_2CH_3$ x 2), 47.8 (NCH_2), 47.6 (NCH_2), 47.3 (NCH_2), 46.5 (NCH_2), 46.1 (NCH_2), 46.0 (NCH_2), 45.7 (NCH_2), 40.9 (NCH_3), 40.7 (NCH_3), 32.8 ($\underline{C}H_2CH$), 32.5 ($\underline{C}H_2CH$), 31.8 ($\underline{C}H_2CH$), 31.7 ($\underline{C}H_2CH$), 31.1 ($\underline{C}H_2CH$), 30.9 ($\underline{C}H_2CH$), 24.3 (CH_2 - pyrrolidine), 24.3 (CH_2 - pyrrolidine), 23.0 ($\underline{C}H_2CH_2NCH_3$), 22.8 ($\underline{C}H_2CH_2NCH_3$), 12.5 (2 x CH_3).

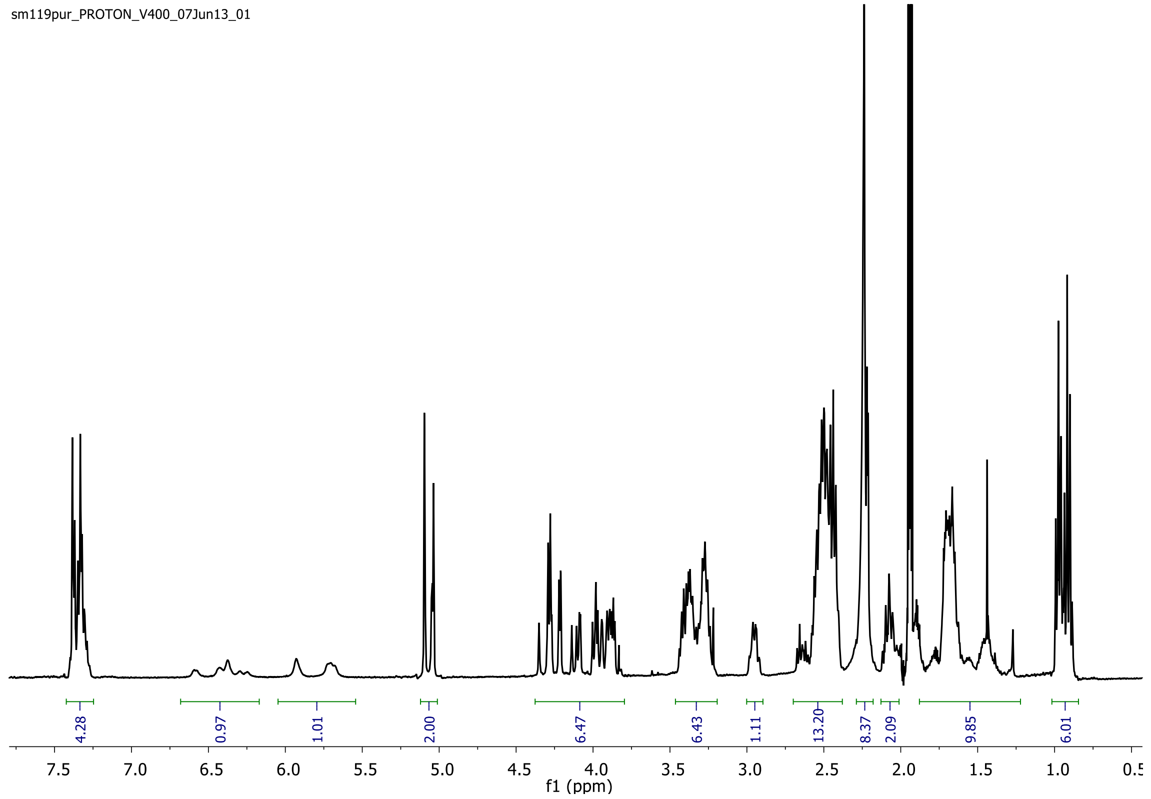

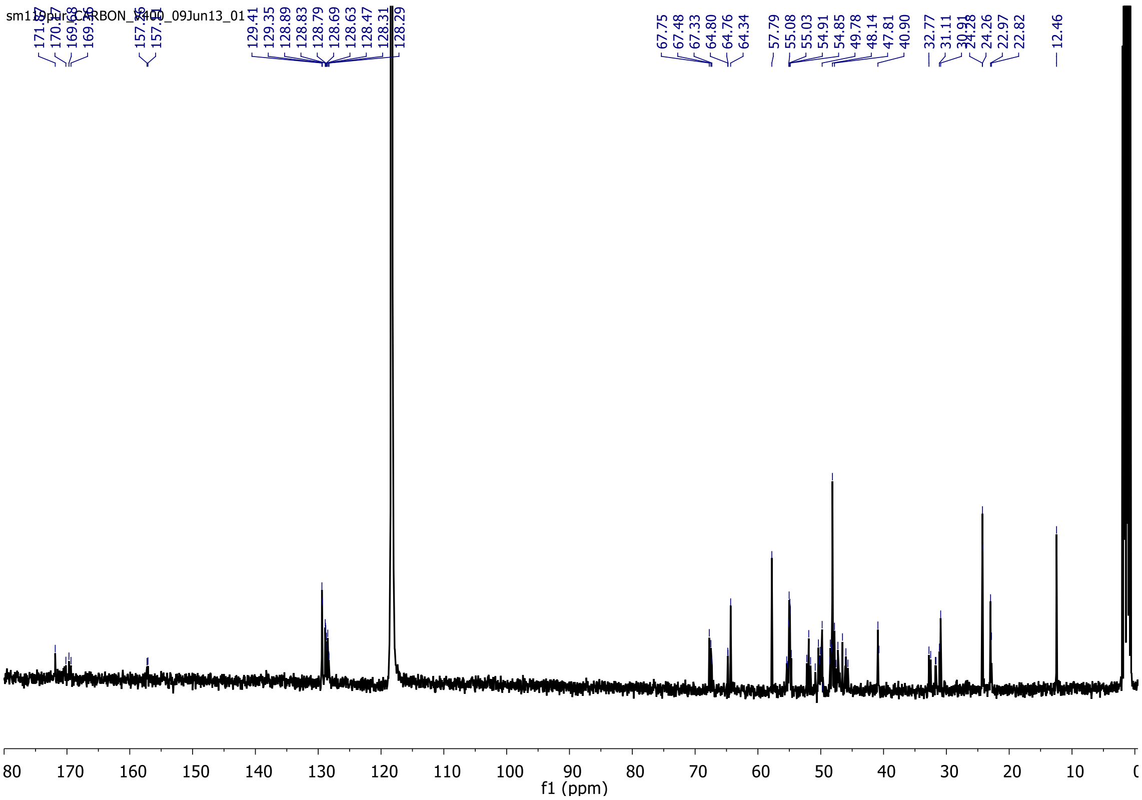
129.41
129.35
128.89
128.83
128.79
128.69
128.63
128.47
128.31
128.29
67.75
67.48
67.33
64.80
64.76
64.34
57.79
55.08
55.03
54.91
54.85
49.78
48.14
47.81
40.90
32.77
31.11
30.91
24.28
24.26
22.97
22.82
12.46
f1 (ppm)

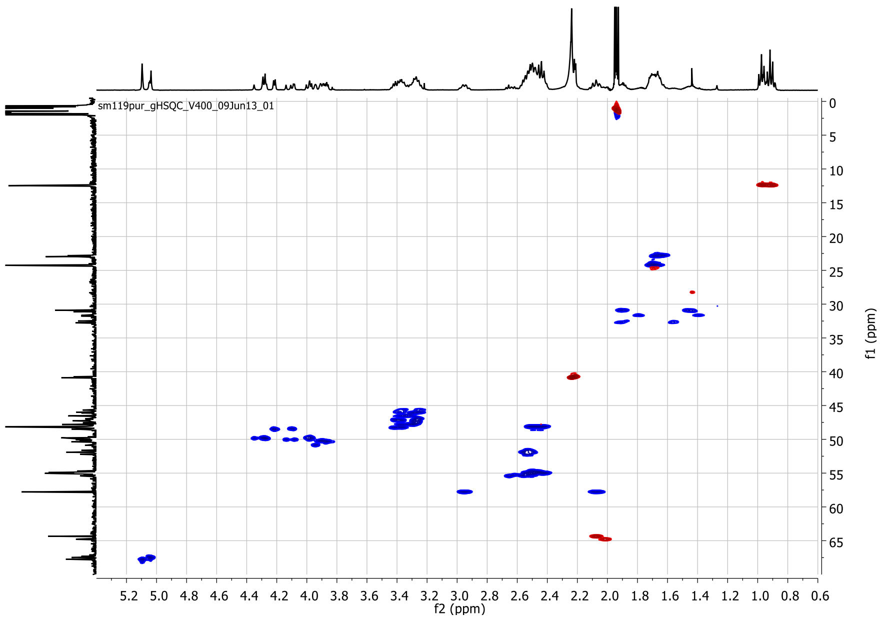
sm119pur_gHSQC_V400_09Jun13_01
f2 (ppm)
f1 (ppm)

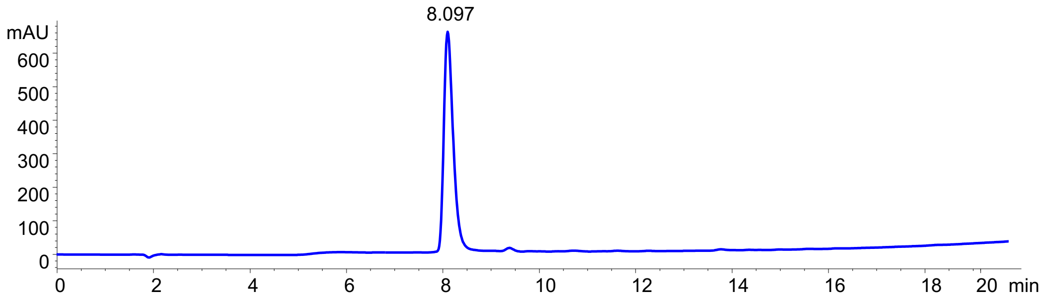

Calculated for **CSIC-002**

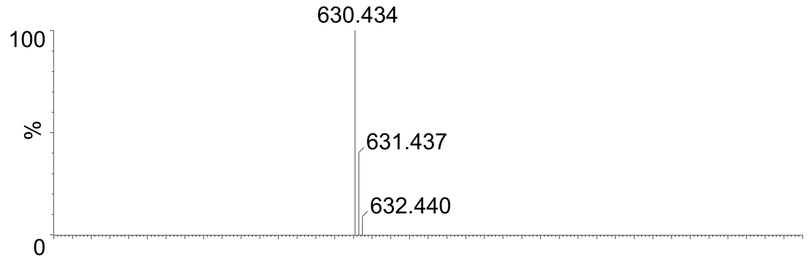

Found for **CSIC-002**

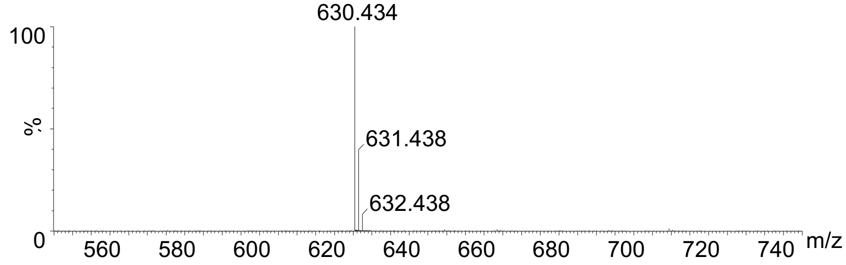

Synthesis of CSIC-03 and CSIC-04

First step: Synthesis of the trimer (CSIC-04)

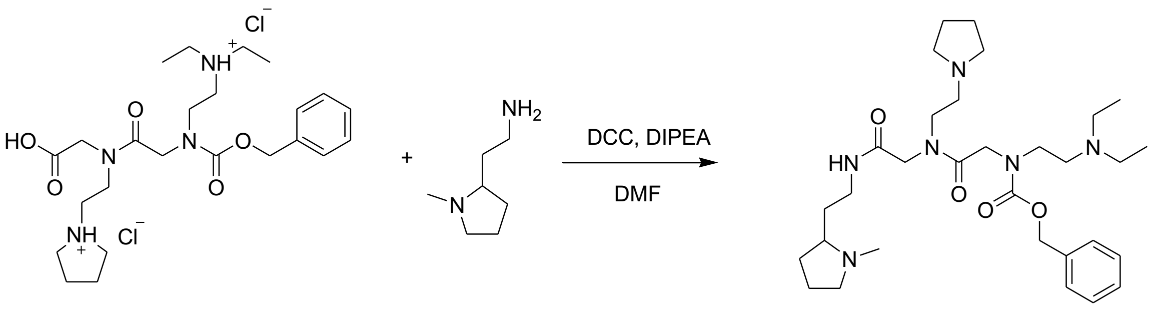

CSIC-004

To a solution of 3-(2-pyrrolidin-1-yl)ethyl-6-benzyloxycarbonyl-9-diethyl-4-oxo-3,6,9-triazanonanoic acid dihydrochloride (100 mg, 0.22 mmol) in DMF, diisopropylamine (113 μL, 0.65 mmol) and DCC (63 mg, 0.30 mmol) were added. The mixture was stirred for 30 minutes and 2-(2-aminoethyl)-1-methylpyrrolidine (31 μL, 0.22 mmol) dissolved in DMF was added. The new mixture was stirred at 50ºC for 24 hours and the solvent was evaporated. Acetonitrile was added to the crude mixture, it was filtrated and evaporated in vacuum. The residue was purified by reverse-phase chromatography using CH_3CN / H_2O as eluent (from 5% to 25%) to give 78 mg (0.14 mmol, 62%). HRMS (M + 1): calcd. for $C_{31}H_{52}N_6O_4$: 573.4128. Found: 573.4107.

^{1}H NMR (400 MHz, rotamers, D_2O): δ (ppm): 7.48 (m, 5H, H_{Ar}), 5.23 (s, CH_2-Cbz), 5.17 (s, CH_2-Cbz), 5.13 (s, CH_2-Cbz), 4.37-4.07 (m, 4H, $COCH_2$), 3.77 (m, NCH_2), 3.72 (m, NCH_2-pyrrolidine), 3.69 ($\underline{CH_2}NCH_3$), 3.42-3.25 (m, $CONH\underline{CH_2}$ x $\underline{CH_2}CH_3$), 3.14 (m, 2 x $\underline{CH_2}CH_3$, 1 x $\underline{CH_2}NCH_3$), 2.95-2.81 (m, NCH_3), 2.39 (m, 1H, $CH\underline{CH_2}$), 2.2-1.9 (m, $CH\underline{CH_2}$, $CHNCH2\underline{CH_2}$, $NCH_2\underline{CH_2}$-pyrrolidine), 1.80 (m, $CH\underline{CH_2}$), 1.28 (m, $CH_2\underline{CH_3}$), 1.16 (m, $CH_2\underline{CH_3}$)

^{13}C NMR (101 MHz, D_2O): δ (ppm): 172.5 (CO), 171.6 (CO), 170.9 (CO), 170.5 (CO), 169.4 (CO), 157.08 (COO), 135.1 (C_{Ar}), 128.1 (CH_{Ar}), 68.8 ($\underline{C}H_2Cbz$), 66.9 (CH), 55.9 ($\underline{C}H_2NCH_3$), 54.5 (NCH_2-pyrrolidine), 52.2 (NCH_2), 51.6 (NCH_2), 49.6 ($CO\underline{C}H_2N$), 47.3 ($\underline{C}H_2CH_3$), 43.7 (NCH_2), 38.9 (NCH_3), 36.3 ($CONH\underline{C}H_2$), 29.6 ($CH\underline{C}H_2$), 28.9 ($CH\underline{C}H_2$), 22.6 ($NCH_2\underline{C}H_2$-pyrrolidine), 22.5 ($NCH_2\underline{C}H_2$-pyrrolidine), 21.0 ($CHNCH_2\underline{C}H_2$), 8.0 ($CH_2\underline{C}H_3$), 7.8 ($CH_2\underline{C}H_3$).

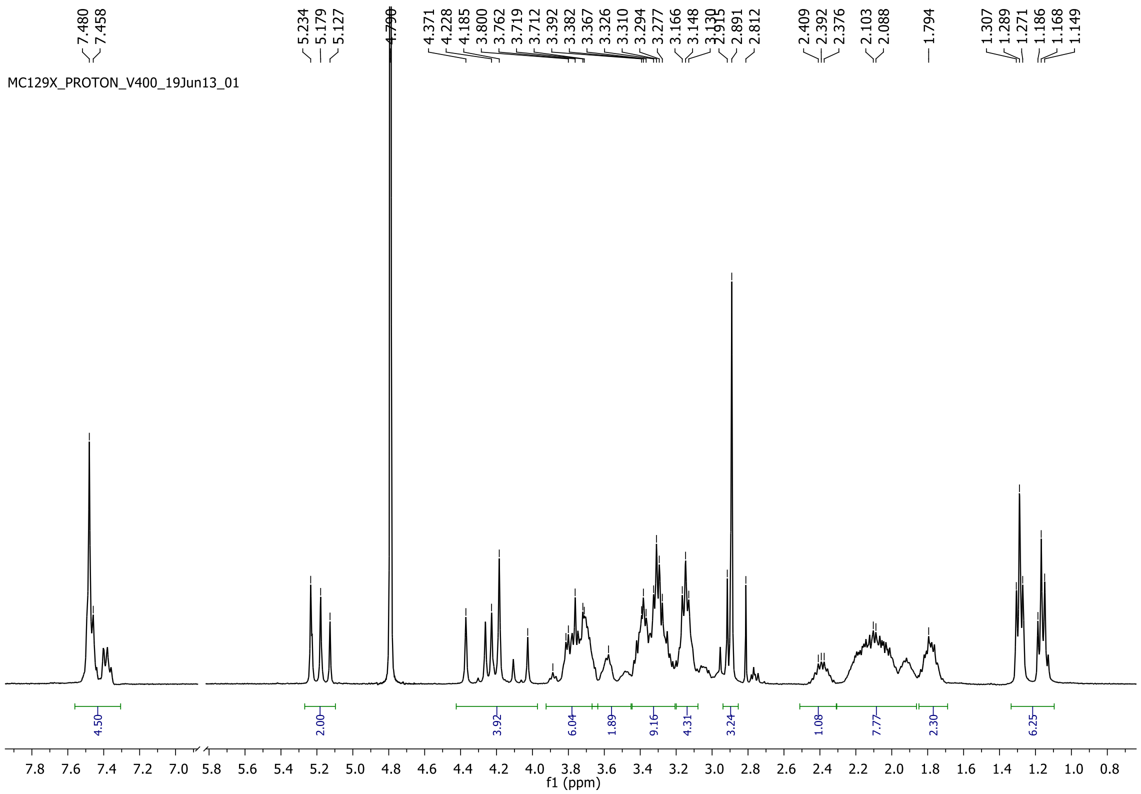
MC129X_PROTON_V400_19Jun13_01
f1 (ppm)

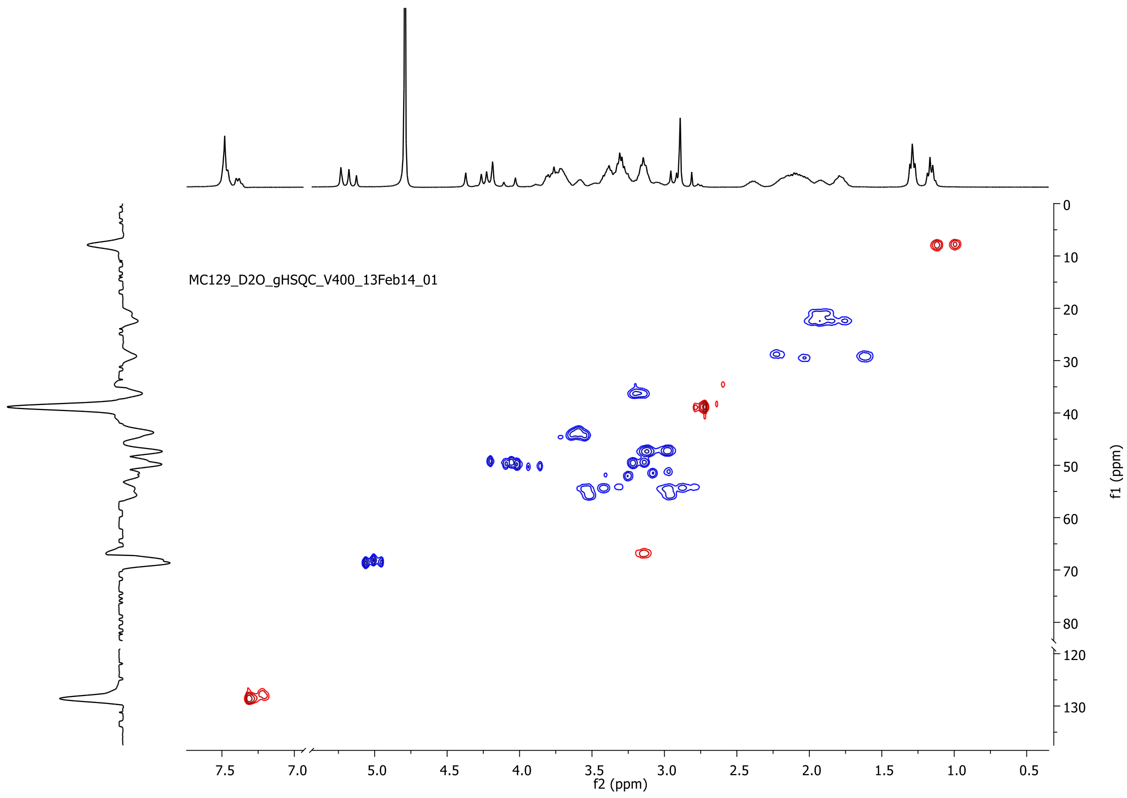
MC129_D2O_gHSQC_V400_13Feb14_01
f2 (ppm)
f1 (ppm)

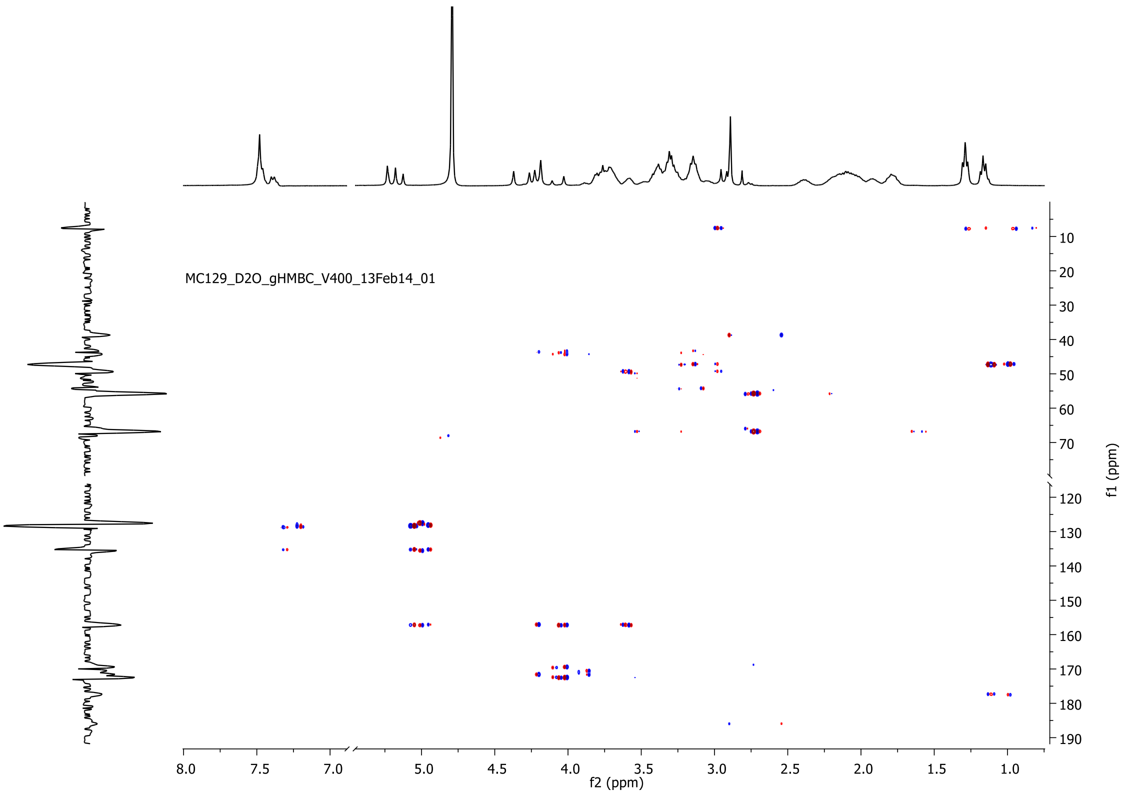

MC129_D2O_gHMBC_V400_13Feb14_01
f1 (ppm)
f2 (ppm)

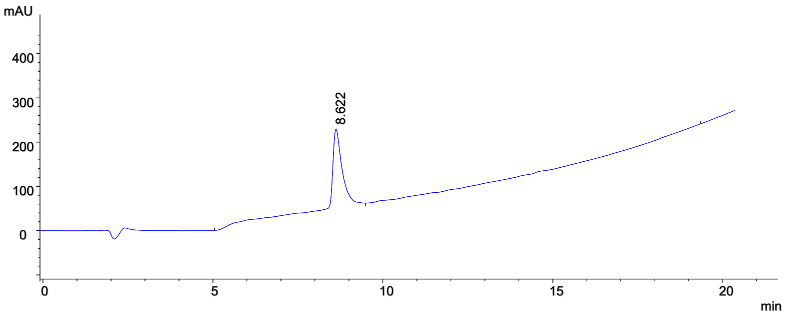

mAU
8.622
min

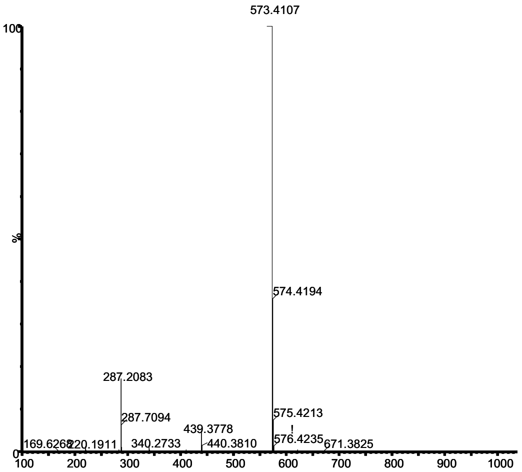

573.4107
574.4194
287.2083
287.7094
439.3778
575.4213
576.4235
169.6268
220.1911
340.2733
440.3810
671.3825
%

Second step: Preparation of CSIC-03

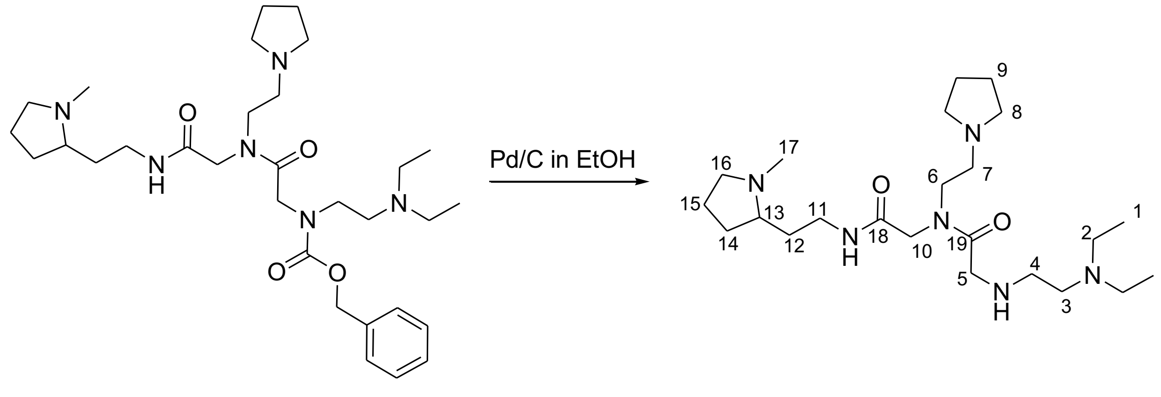

40 mg (0.07 mmol) of CSIC-004 dissolved in 2 mL of ethanol was added to a round-bottom flask containing 7 mg of Pd/C in 1 mL of EtOH. The mixture was stirred under hydrogen atmosphere at 1 atm at room temperature overnight. The product was isolated by filtering off the catalyst and washing with EtOH. The combined filtrate was evaporated under reduced pressure obtaining 15 mg (0.03 mmol, 50%) of the desired product. HRMS (M + 1): calcd. for $C_{23}H_{46}N_6O_2$: 439.3760. Found: 439.3717.

^{1}H NMR (400 MHz, 2 rotamers, 25%/75%, D$_2$O): δ (ppm): 4.21 (s, 2H, NCH_2CO), 4.11 (s, 2H, NCH_2CO), 3.91 (m, $COCH_2NH$), 3.79 (t, *J* = 6.3 Hz, 2H, NCH_2), 3.73 (m, 6H, N$\underline{CH_2}$-pyrrolidine, $COCH_2NH$), 3.69 (m, 2H, $\underline{CH_2}NCH_3$), 3.51 (m, 2H, CH_2N), 3.42 (t, *J* = 6.3 Hz, 2H, CH_2N), 3.37 (m, 2H, $NHCH_2\underline{CH_2}N$), 3.36 (m, 2H, $NH\underline{CH_2}CH_2CH$), 3.33 (m, 1H, CH), 3.28 (m, 4H, $N\underline{CH_2}CH_3$), 3.19-3.12 (m, 6H, N$\underline{CH_2}$-pyrrolidine $\underline{CH_2}NCH_3$) 3.18 (m, 2H, $NH\underline{CH_2}CH_2N$), 2.93 (m, 3H, NCH_3), 2.4 (m, 2H, $NCH\underline{CH_2}$), 2.2-2.0 (m, 6H, $CHCH_2\underline{CH_2}$, $NCH_2\underline{CH_2}$pyrrolidine, 1.8 (m, 4H, $\underline{CH_2}CH$), 1.32 (m, 6H, $CH_2\underline{CH_3}$)

^{13}C NMR (101 MHz, D$_2$O): δ (ppm): 172.0 (CO), 170.9 (CO), 169.7 (CO), 166.1 (CO), 66.9 (CH), 55.9 ($CH_3N\underline{C}H_2$), 54.5 (NCH_2-pyrrolidine), 51.9 (NCH_2), 51.7 (NCH_2), 50.0 ($CO\underline{C}H_2N$), 49.5 ($CO\underline{C}H_2N$), 49.3 ($\underline{C}H_2NCH_2CH_3$), 48.6 ($CO\underline{C}H_2NH$), 47.5 ($N\underline{C}H_2CH_3$), 43.8 ($NCH_2$), 42.1 ($NH\underline{C}H_2CH_2N$), 38.9 ($NCH_3$), 36.4 ($CONH\underline{C}H_2$), 29.5 ($CH\underline{C}H_2$), 28.9 ($CH\underline{C}H_2$), 22.5 ($NCH_2\underline{C}H_2$-pyrrolidine), 20.9 ($CHCH_2\underline{C}H_2$), 7.8 ($CH_2\underline{C}H_3$)

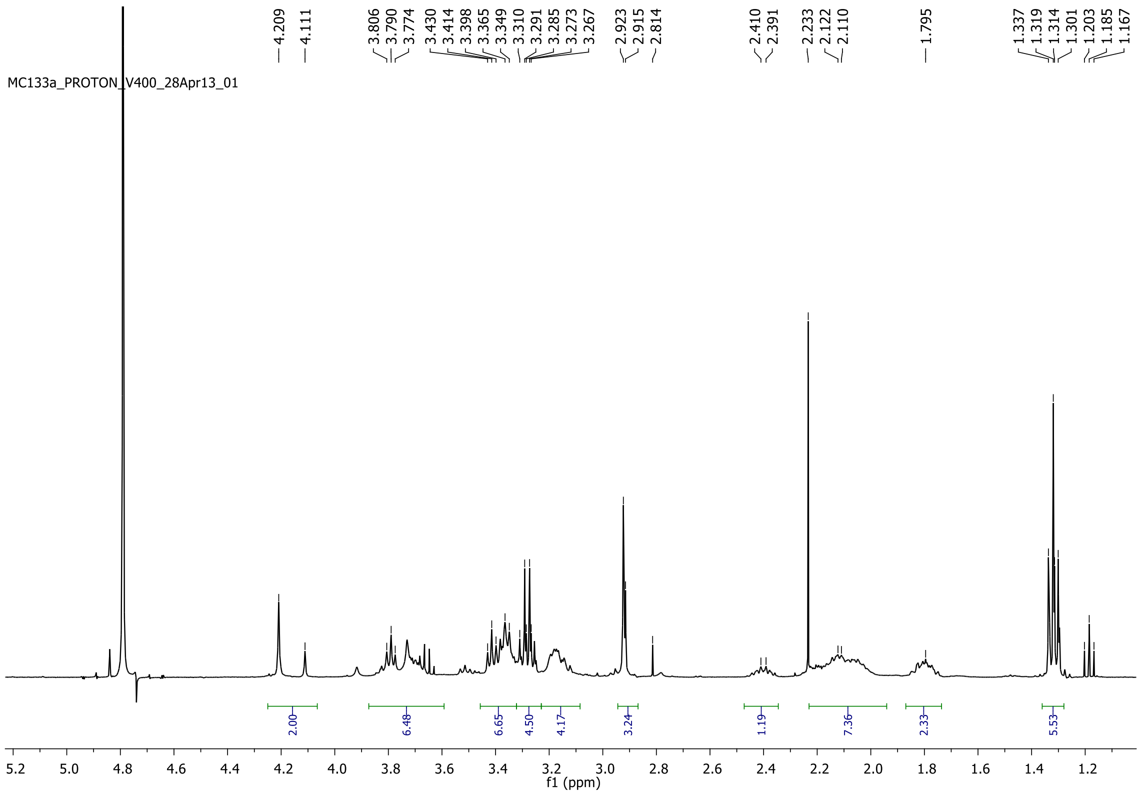
MC133a_PROTON_V400_28Apr13_01
4.209
4.111
3.806
3.790
3.774
3.430
3.414
3.398
3.365
3.349
3.310
3.291
3.285
3.273
3.267
2.923
2.915
2.814
2.410
2.391
2.233
2.122
2.110
1.795
1.337
1.319
1.314
1.301
1.203
1.185
1.167
2.00
6.48
6.65
4.50
4.17
3.24
1.19
7.36
2.33
5.53
f1 (ppm)

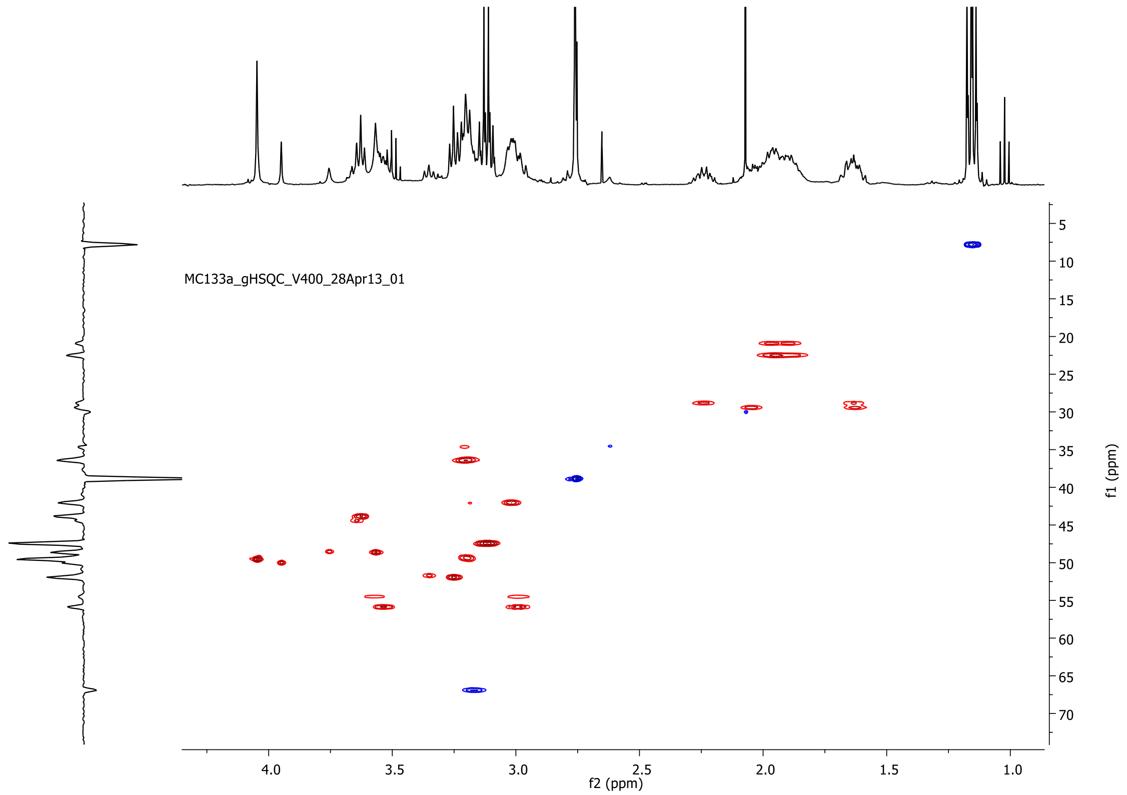
MC133a_gHSQC_V400_28Apr13_01
f1 (ppm)
f2 (ppm)

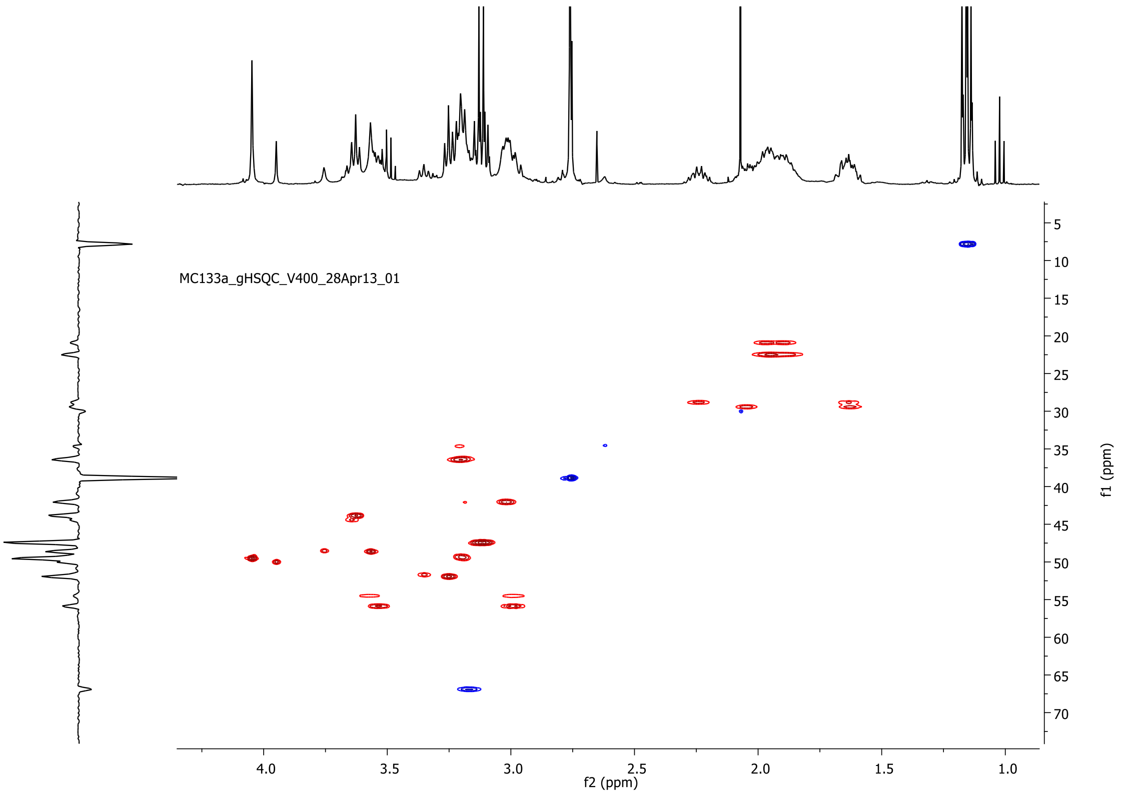
MC133a_gHSQC_V400_28Apr13_01
f1 (ppm)
f2 (ppm)
5
10
15
20
25
30
35
40
45
50
55
60
65
70
4.0
3.5
3.0
2.5
2.0
1.5
1.0

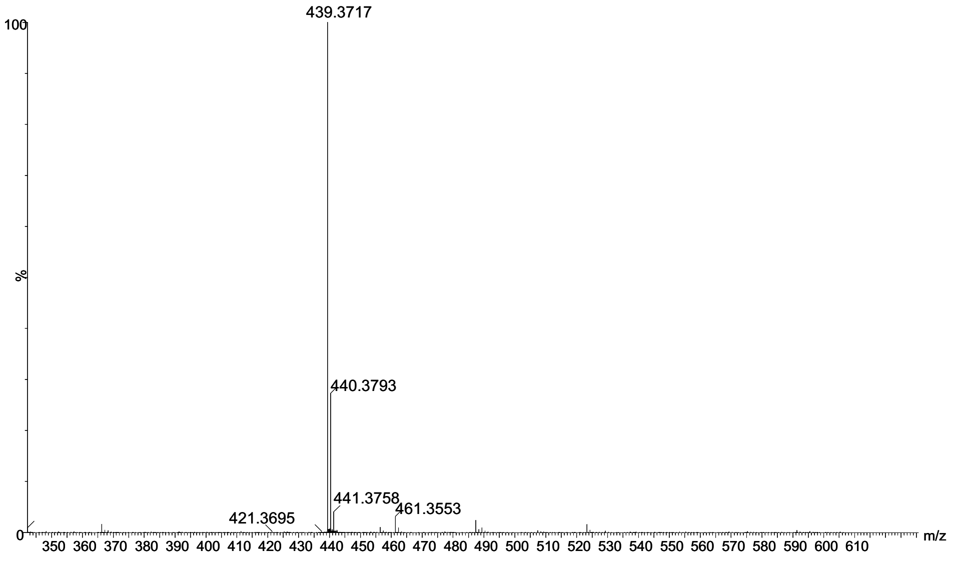
439.3717
100
%
0
440.3793
441.3758
461.3553
421.3695
m/z
350 360 370 380 390 400 410 420 430 440 450 460 470 480 490 500 510 520 530 540 550 560 570 580 590 600 610
